# Tunable Excimer Circularly Polarized Luminescence in Isohexide Derivatives from Renewable Resources

**DOI:** 10.1002/chem.202104226

**Published:** 2022-02-15

**Authors:** Valerio Zullo, Anna Iuliano, Gennaro Pescitelli, Francesco Zinna

**Affiliations:** ^1^ Dipartimento di Chimica e Chimica Industriale University of Pisa Via Moruzzi 13 Pisa 56124 Italy

**Keywords:** circularly polarized luminescence, excimer, isomannide, pyrene, TD-DFT

## Abstract

Organic compounds showing circularly polarized luminescence (CPL) are at the forefront of novel applications and technologies. Here we show the synthesis and chiroptical properties of pyrene and perylene derivatives of inexpensive chiral scaffolds: isomannide and isosorbide. Low‐intensity ECD spectra were obtained, suggesting the absence of chromophore interaction in the ground state, except in the case of isomannide bis‐perylenecarboxylate, whose ECD spectrum showed a positive exciton couplet. All isomannide derivatives, with the only exception of the one containing a pyrenecarboxylate and a perylenecarboxylate, exhibited excimer CPL spectra, whereas isosorbide derivatives did not show any CPL. Isomannide derivatives bearing two pyrenecarboxylate or two pyrenylacetate groups showed positive CPL emission with dissymmetry factors up to 10^−2^, which depends on the conformational freedom of the appended units. The CPL sign, Stokes shift and order of magnitude of dissymmetry factor were reproduced by excited‐state calculations on a representative compound. Interestingly, the mixed derivative containing pyrenic units with different spacing from the isomannide scaffold showed an oppositely signed excimer band with respect to the homo‐substituted derivatives.

## Graphical abstract

**A simple and economical protocol for obtaining new CPL‐emitting materials** starting from natural chiral scaffold was developed. By choosing the scaffold, the aromatic moiety and the spacer, fine tuning of CPL and especially inversion of the preferential circular polarization of the emitted light was obtained.

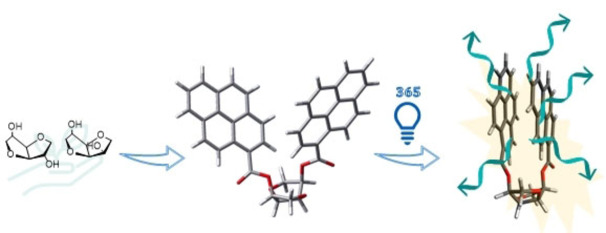

## Introduction

Circularly polarized luminescence (CPL), defined as the emission of circularly polarized light with a predominant handedness, is currently attracting increasing attention. Different applications regarding CPL emitting materials have been proposed and developed,[^1^, ^2^, ^3^, ^4^, ^5^, ^6^] such as CP‐OLEDs,[^7^, ^8^, ^9^] spintronic‐based devices,^[10]^ biological probes,[^11^, ^12^] optical information storage and processing,[^13^, ^14^] CPL lasers,[^15^, ^16^] CPL‐based chiroptical switches,^[17]^ and light‐emission systems for asymmetric photosynthesis.^[18]^ There is therefore a growing interest in developing new organic molecules able to efficiently emit CPL. Moreover, to expand the applicability of such systems, it may be important to design and prepare molecules which can be easily tuned in terms of CPL sign and intensity and emission wavelength through chemical synthesis.

In general, the CPL activity may be quantified through the luminescence dissymmetry factor (*g_lum_* =2(I_L_‐I_R_)/(I_L_+I_R_), I_L_ and I_R_ being the left and right circularly polarized component of the emission), and additionally by the so‐called CPL brightness (B_CPL_=ϵ_λ_ ⋅ Φ ⋅ |g_lum_|/2). This quantity, beside g_lum_, considers emission quantum yield (Φ) and extinction coefficient (ϵ_λ_),^[19]^ which are crucial factors in any application of CPL‐active emitting materials.

Among CPL‐active organic emitters, chiral systems allowing for intramolecular excimer formation achieve the highest B_CPL_ values.^[19]^ In particular, interesting results can be obtained with perylene and mainly pyrene‐based chiral systems. As the electronically excited dimer, giving rise to excimer emission, exists only in the excited state, the associated g_lum_ is not related to its absorption counterpart (g_abs_). This is in contrast with what observed with most organic molecules where g_lum_ correlates with the g_abs_ of the most red‐shifted Cotton effect.^[20]^ Indeed, in most examples of pyrene‐based chiral compounds, g_lum_ is one or two orders of magnitude higher than it would be expected from their CD spectrum.^[21]^

In order to fully exploit the advantage brought about by excimer emission in the context of CPL, carefully selected chiral scaffolds bearing the fluorophores are essential. Such scaffolds should maintain the fluorophore units in close proximity to foster the intramolecular excimer formation and provide the stereochemically ordered arrangement necessary for efficient excimer CPL. A few examples of significant excimer CPL were obtained by using as the chiral scaffold oligopeptides[^22^, ^23^, ^24^, ^25^, ^26^] or nucleic acids,^[27]^ binaphtyls and derivatives,[^28^, ^29^, ^30^, ^31^, ^32^, ^33^] dioxolanes‐,^[34]^ tetrathiazole‐,^[35]^ dibenzofuran‐,^[36]^ cyclophane‐,^[37]^ and phenylene‐ethynylene‐based compounds,^[38]^ quinoline oligoamide foldamers^[39]^ and polyether‐like macrcocycles.^[40]^

**Figure 1:**
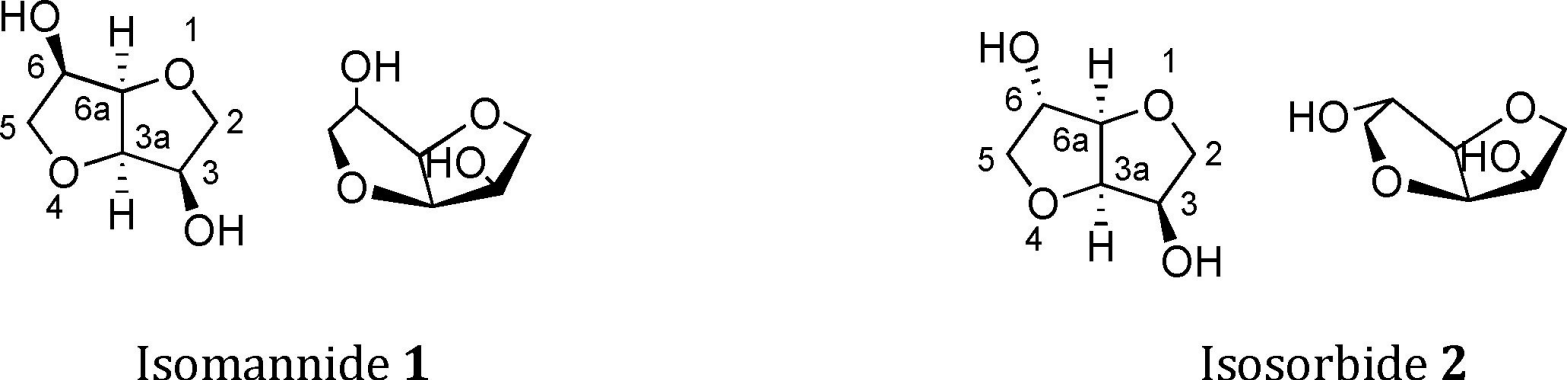
Isomannide (1) and isosorbide (2).

From a synthetic point of view, such chiral scaffolds should have suitable functional groups to be derivatized with the chosen emitting units. Moreover, it would be advantageous to employ chiral scaffolds which are inexpensive, readily accessible, and straightforwardly derivatizable. Starting from these considerations we reasoned that, in the search of new CPL‐emitting excimers, great advantages could be taken starting from isohexides as the central chiral scaffolds. Isohexides, namely isomannide (1) and isosorbide (2), also known as (3R,3aR,6R,6aR)‐hexahydrofuro[3,2‐b]furan‐3,6‐diol and (3R,3aR,6S,6aR)‐hexahydrofuro[3,2‐b]furan‐3,6‐diol (Figure 1), are important by‐products of the starch industry, arising from dehydration of D‐mannitol and D‐sorbitol.^[41]^

**Figure 2:**
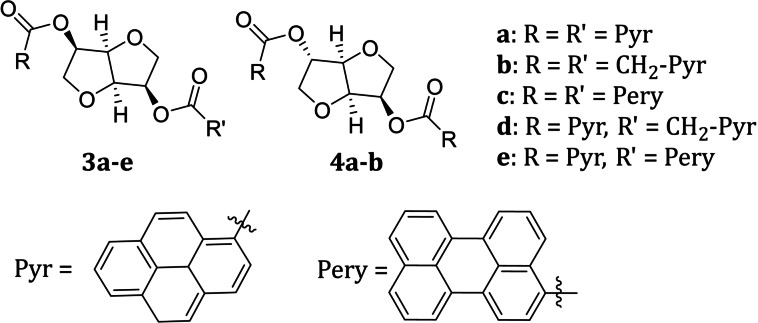
Structure of the CPL emitting isohexide derivatives.

These commercially available starting materials provide an easy and inexpensive access to optically pure functionalized compounds. Indeed, through a simple derivatization of the two hydroxyl groups, the characteristic chiral cavity of their scaffold (Figure 1) can be functionalized, thus leading to new compounds, whose properties depend not only on the nature of the introduced moieties, but also on the different stereochemistry of the native hydroxyl groups. Thanks to these characteristics, isohexides were successfully employed as starting materials in the preparation of chiral ligands,^[42]^ organocatalysts^[43]^ and chiral ionic liquids.[^44^, ^45^] In particular, starting from isomannide, bidentate ligands[^42^, ^46^, ^47^, ^48^] and ionic‐liquid molecular tweezers[^45^, ^49^] were obtained by virtue of the *endo* arrangement of the hydroxyl groups, which allows the appended units to be sufficiently close to each other. However, as we recently demonstrated,^[49]^ the interaction of two appended moieties was even observed in some isosorbide derivatives, due to their particular spatial arrangement. This evidence highlighted the chance to exploit the isohexide chiral scaffold in the preparation of new emitting molecules displaying intramolecular excimer CPL (Figure 2). To this goal, derivatives 3a–b and 4a–b, possessing pyrene as the emitting moiety, were synthesized, in order to investigate the effect of the different stereochemical arrangement and conformational freedom on the emission and CPL properties. We also synthesized derivative 3c containing two perylene fluorophores to assess the influence of the emitting moiety on excimer emission. Mixed derivatives 3d‐e, built on the isomannide structure, were also investigated.

## Results and Discussion

### Synthesis

**Scheme 1:**
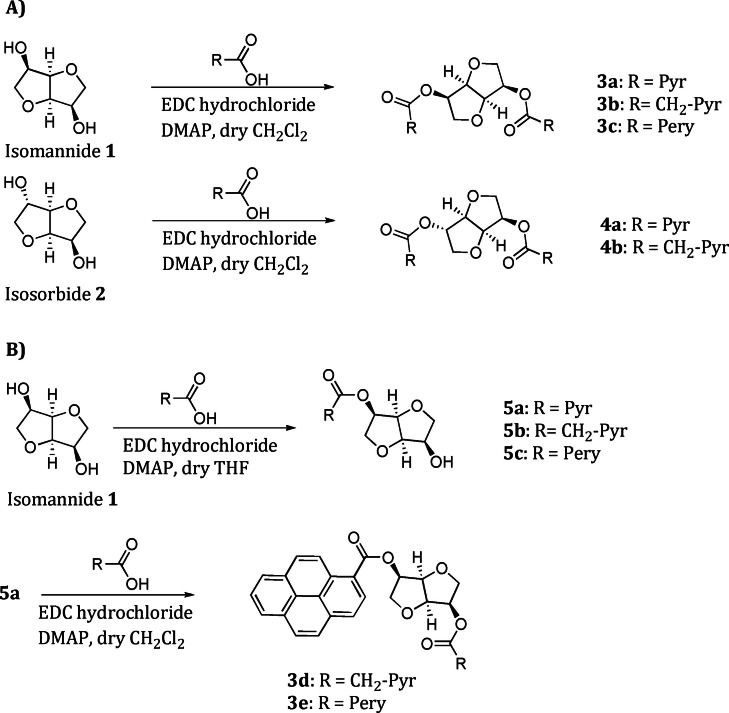
Syntesis of the CPL emitting isohexide derivatives.

The preparation of isohexide derivatives bearing two identical appended moieties was straightforwardly realized by reacting isomannide 1 (or isosorbide 2) with a slight excess of the appropriate carboxylic acid in the presence of ethyldimethylamino carbodiimide (EDC) hydrochloride and dymethylaminopyridine (DMAP) for 24 h, under standard reaction conditions (Scheme 1, A). The diesters 3a–b and 4a–b were obtained in good yields (ranging from 66 to 81 %), after recrystallization of the crude products. The preparation and isolation of the perylenic derivative 3c was more problematic, due to its higher steric hindrance and its low solubility in a great number of organic solvents.

The mixed derivatives 3d‐e were prepared in two steps (Scheme 1, B). The mono‐derivatization of isomannide was obtained by reacting 1‐pyrenecarboxylic acid with a threefold excess of 1, in order to avoid the formation of the disubstituted by‐product. Under these reaction conditions compound 5a was obtained in 41 % yield, after chromatographic purification. In a similar manner, monoester derivatives 5b–c were obtained (see Supporting Information).

The reaction of 5a with an excess of 1‐pyrenylacetic acid or 3‐perylenecarboxylic acid, under the reaction conditions used for the preparation of the other diesters, afforded derivatives 3d and 3e, respectively. In this case too, the yield of the reaction was affected by the size of the derivatizing acid, being lower (39 %) in the case of derivative 3 e, where the bulkier 3‐perylenecarboxylate moiety was introduced. All the derivatives were fully characterized (see Supporting Information).

### Spectroscopic properties

**Figure 3:**
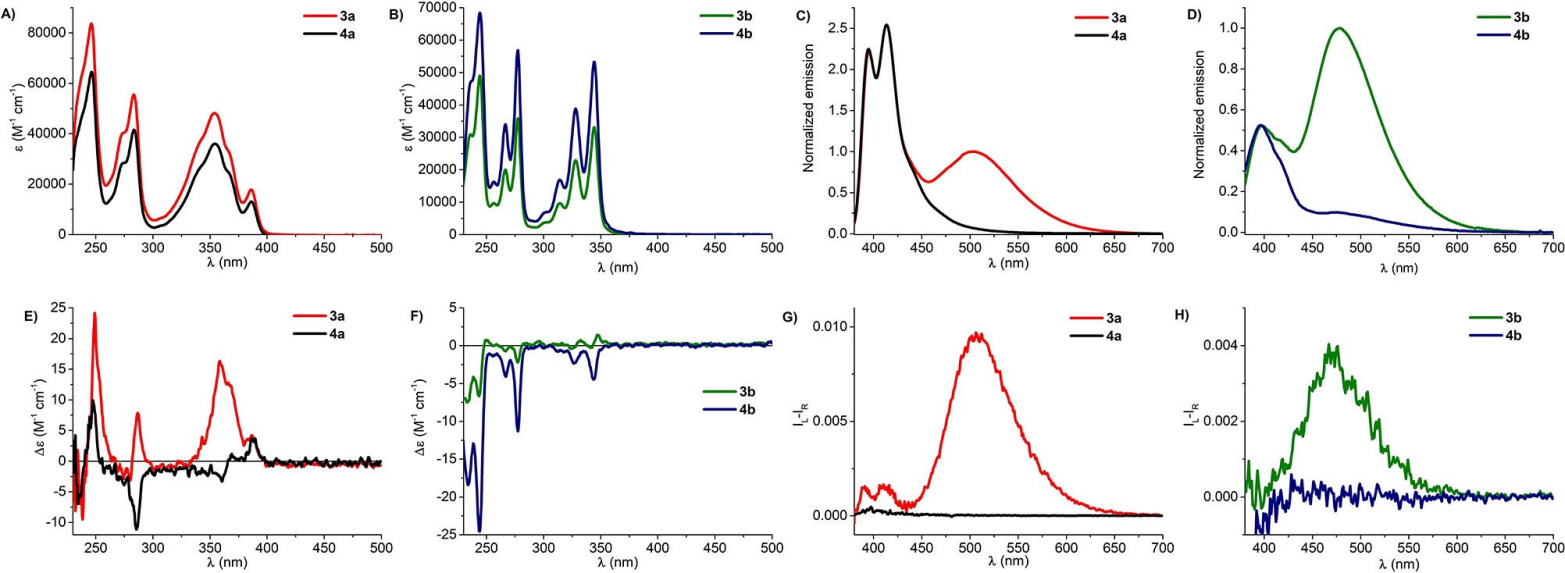
UV‐Vis (**A**, **B**), ECD (**E**, **F**), fluorescence (**C**, **D**) and CPL (**G**, **H**) spectra of compounds 3a (red line; 1.7 ⋅ 10^−5^ M), 4a (black line; 1.4 ⋅ 10^−5^ M), 3b (dark green line; 1.7 ⋅ 10^−5^ M) and 4b (dark blue line; 1.6 ⋅ 10^−5^ M) recorded in CH_2_Cl_2_ as the solvent (excitation wavelength 365 nm).

UV‐Vis, ECD, fluorescence and CPL spectra of diester derivatives 3, 4 and reference monoesters 5 were recorded in CH_2_Cl_2_ as the solvent with a concentration of ∼10^−5^ M (see Supporting Information). Each compound showed different absorption bands in UV‐Vis spectra between 230 and 500 nm, with a molar extinction coefficient ϵ spanning from 10^3^ to 10^4^ M^−1^ cm^−1^, according to the nature of the major chromophore (pyrene or perylene). Compounds 3b, 4b and 5b showed the typical absorption pattern of pyrene,[^50^, ^51^] featuring the 2B_3u_, 1B_2u_ and 2B_2u_ transitions (^1^L_a_, ^1^B_b_ and ^1^B_a_ in Platt's nomenclature) from long to short wavelengths, each with pronounced vibronic structure. Compounds 3a, 4a (Figure 3A) and 5a (Figure S26) showed a different absorption profile, with a faint and altered vibronic pattern and an extra band at 386 nm. This latter is not due to intramolecular interaction between the pyrene rings in the diesters,^[52]^ as it appears also in the monoester 5a; it can be assigned to 1B_3u_ transition (^1^L_b_ in Platt's nomenclature) which is forbidden in pyrene but acquires oscillator strength upon substitution with the ester moiety at C‐1 (see Supporting Information, Table S7 and Figures S45–S46).^[53]^ Interestingly enough, the same band is also visible for the “mixed” compounds 3d and 3e (Figure S24, S25). The perylene derivatives 3c and 5c are characterized by broad absorption bands devoid of vibronic patterns, different from the parent perylene.[^51^, ^54^] This is also due to the substitution with the ester moiety.

The ECD spectra of compounds 3‐5 showed the presence of several bands (Cotton effects), each in correspondence with an absorption band. Generally speaking, all ECD spectra are quite weak, with absolute Δϵ values below 10–20 M^−1^ cm^−1^ and corresponding dissymmetry factors *g*_abs_ below 3 ⋅ 10^−4^. The ECD spectra of compounds 3a, 4a, 3b and 4b are reported in Figure 3 (all other spectra can be found in the Supporting Information).

A striking evidence from the ECD spectra of diesters 3 and 4 is the absence of exciton‐like features, with the only exception of perylene‐derivative 3c.[^55^, ^56^, ^57^, ^58^] Together with the observed low intensities, this observation suggests that in the ground state the two aromatic moieties of the diesters lie far apart and/or adopt a floppy reciprocal arrangement. This evidence was also confirmed by ^1^H NMR spectra which do not show significant chemical shift differences for pyrene signals moving from monoester 5a to 3a (Figure S34), confirming the lack of pyrene‐pyrene stacking interactions in the ground state. Geometry optimizations with DFT (density functional theory) method and ECD calculations with TD‐DFT (time dependent DFT) method were run on 3a, as representative of the series (see Supporting Information), using Gaussian’16 software.^[59]^ Several functionals were employed both in the geometry optimizations and in ECD calculations (B3LYP, B3LYP‐D3, BH&HLYP, BH&HLYP‐D2). The calculations did not reproduce the experimental ECD spectra (Figure S40). The possible reason for this outcome is twofold. First, the lowest‐energy conformers showed a preference for structures with strongly interacting pyrene rings, by π‐π or C−H‐π interaction (Table S1 and Figures S38–S39), while NMR data suggest a more heterogeneous conformational situation. Rather than a failure of the DFT method in predicting the correct ground‐state geometry of 3a, we ascribe this result to an insufficient conformational search procedure; more exhaustive conformational sampling, based for instance on molecular dynamics, may need to be employed for a better treatment of 3a and related systems, which is beyond the scope of the present paper. We noticed that when the most stable conformers with π‐stacked pyrene rings were employed for ECD calculations, they consistently led to strong exciton‐split ECD spectra with *g*_abs_ values exceeding 10^−3^. This offers an indirect proof that such stacked conformations are not dominant in the conditions of measurements of ECD spectra. Another possible source of error in the present ECD calculations is the well‐known difficulty of TD‐DFT in predicting the order of electronic transitions of pyrene, although the correct order should be recovered by employing CAM‐B3LYP.[^51^, ^60^, ^61^] To summarize, the evidence from absorption, ECD and NMR spectra suggests no stacking interaction between the *cis*‐arranged aromatic rings of isomannide pyrene diesters in the ground state.

The emission of pyrene‐containing derivatives strongly depends on the stereochemistry of the isohexide scaffold. In the case of functionalized isosorbide scaffold, where the fluorophores adopt an *anti* geometry, no significant interaction can occur between the aromatic moieties. Accordingly, the isosorbide derivative 4a, bearing two pyrenecarboxylate moieties, displays only an emission band associated to monomeric pyrene (λ=395 and 413 nm). On the other hand, isomannide provides a scaffold in which the aromatic substituents can assume a co‐facial conformation. Thus, the corresponding isomannide derivative 3a shows the presence of a broad and structureless emission band at 502 nm (apparent Stokes shift 116 nm) due to the typical pyrene‐pyrene intramolecular excimer (Figure 3, C).^[21]^ The same trend is observed with derivatives 3b and 4b, bearing two pyrenylacetate moieties on the isomannide and isosorbide scaffold respectively. In this case, fluorescence spectra show the emission bands of the monomer pyrene and a broad emission band attributable to excimer emission (λ=477 nm) for 3b (Figure 3, D), while again compound 4b does not display significant excimer emission. As expected, CPL measurements reveal intense bands associated with excimer emission.^[21]^ Both 3a and 3b were characterized by a positive excimer CPL band with maximum at 500 nm and 475 nm, respectively (Figure 3, G and Figure 3, H). In the case of 3a, the luminescence dissymmetry factor was 2.4 times higher than for 3b (*g_lum_* =9.6 ⋅ 10^−3^ for 3a and 4.0 ⋅ 10^−3^ for 3b). This difference could be ascribed to the presence in 3b of an additional methylene spacer between the pyrene fluorophore and the central chiral scaffold, which increases the rotational freedom of the emitting moieties, as well as the distance between themselves, thus possibly leading to a looser intramolecular interaction.

**Figure 4:**
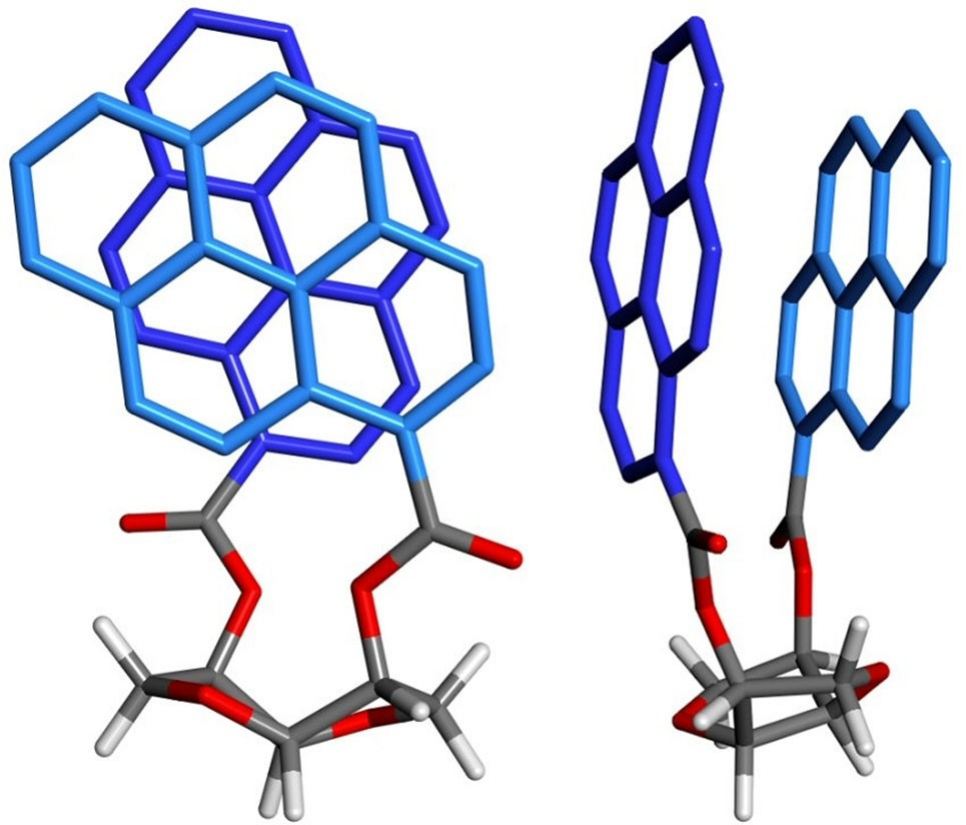
Two viewpoints of the first excited‐state geometry of compound 3a optimized at BH&HLYP/def2‐SVP/CPCM level. The pyrene rings are depicted with different blue hues and their hydrogen atoms are removed for clarity.

Generally speaking, CPL spectra of organic compounds are composed of a single band which parallels the lowest‐lying ECD band both in sign and *g*‐factor.^[20]^ Exceptions to this common rule are observed when the structure of the 1^st^ excited state differs from that of the ground state, as it has been evidenced by some theoretical studies.[^62^, ^63^] In the current case, the excimer naturally differs significantly from the non‐stacked ground state. This is reflected not only in the tenfold or larger *g_lum_* with respect to *g*_abs_, but also in the large observed Stokes shift. With the aim to reproduce the excimer CPL spectrum of 3a, excited state calculations^[64]^ were run with time‐dependent density functional theory (TD‐DFT) using different functionals (B3LYP, B3LYP‐D3, BH&HLYP and BH&HLYP‐D2)[^65^, ^66^] with def2‐SVP basis set in vacuo, using Orca 5.0 software[^67^, ^68^, ^69^] (see Supporting Information). Almost all methods (with the exception of B3LYP) converged toward a small set of excimer‐like structures with short ring‐to‐ring distances (<3.5 Å, Table S2 and Figure S41). One conformation, in particular, was consistently found as by far the most stable one with the other three functionals. Re‐optimization at BH&HLYP/def2‐SVP level including the CPCM implicit solvent model for dichloromethane yielded the excited‐state structure shown in Figure 4, which features a ring‐to‐ring distance of 3.3 Å. Emission/CPL calculations were then run on this structure with various functionals (B3LYP, BH&HLYP and CAM‐B3LYP), two basis sets (def2‐SVP and def2‐TZVP) and CPCM for dichloromethane. The two functionals BH&HLYP and CAM‐B3LYP led, with both basis sets, to similar results (Table S4): the calculated Stokes shifts were 107–113 nm and the calculated *g_lum_* spanned from +3 to +4 ⋅ 10^−2^. In consideration of the already discussed issues related to TD‐DFT calculations on pyrene, we were satisfied to observe a good agreement with the corresponding experimental data. As the excimer structure displayed in Figure 4 leads to calculated data in line with emission and CPL spectra of 3a, it may be taken as a good model of the intramolecular excimer. The nature of the excimer as a combination of ^1^L_b_ states was verified by molecular orbital analysis (Figure S42). Since the excimer state is well‐separated from any other excited state, the common pitfalls associated with pyrene excited‐state ordering by TD‐DFT[^51^, ^60^, ^61^] seem to be relieved.

**Figure 5:**
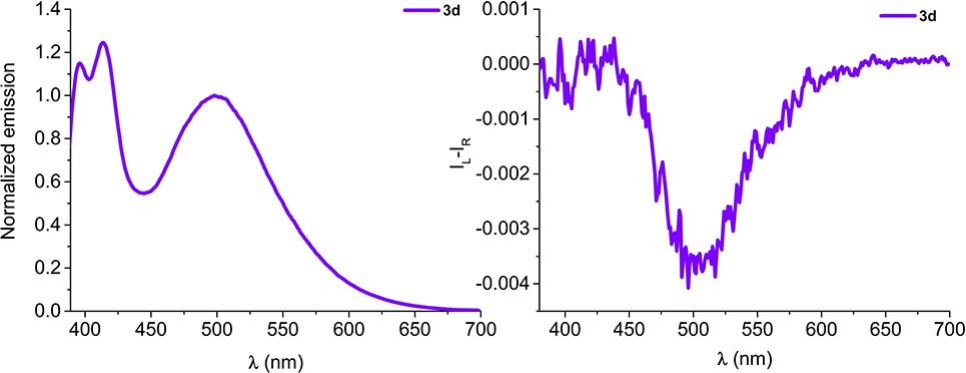
Fluorescence spectra (left side) and CPL spectra (right side) of compound 3 d (1.5 ⋅ 10^−5^ M in CH_2_Cl_2_, excitation wavelength 365 nm).

The fluorescence spectrum of the mixed diester 3 d, containing both pyrenecarboxylate and pyrenylacetate moieties on the isomannide scaffold, still showed the characteristic excimer emission band at 498 nm (Figure 5, left). Much to our surprise, the CPL band allied with excimer emission (Figure 5, right) displayed an opposite sign with respect to homo‐substituted derivatives 3a and 3b. This unexpected behaviour is due to the different conformational space accessible to the hetero‐substituted 3d compound, which leads to an opposite chirality excimer state.^[70]^

The *g_lum_* for 3d is similar in absolute value to the one obtained for 3b (*g_lum_* =−4.0 ⋅ 10^−3^). Even in this case, no interaction between the two chromophores could be observed in the ground state, neither in ECD nor in ^1^H NMR spectra (see Figure S24). Probably due to the increased conformational complexity, the computational procedure employed for 3a failed to capture an adequate excimer structure for 3d (see Supporting Information).

**Figure 6:**
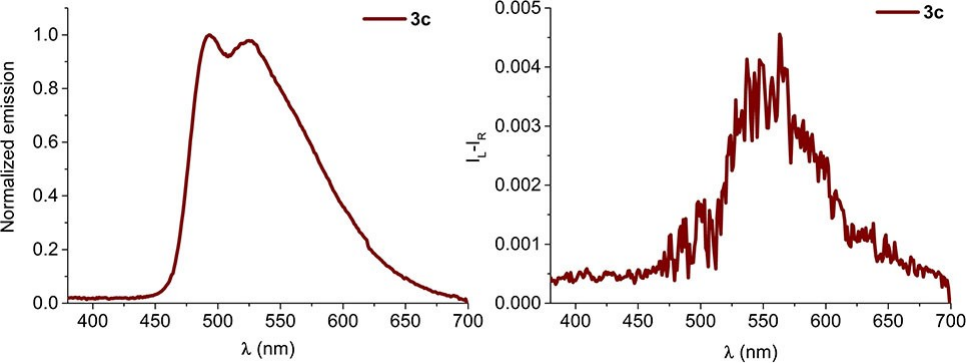
Fluorescence spectra (left side) and CPL spectra (right side) of compound 3c (1.6 ⋅ 10^−5^ M in CH_2_Cl_2_, 1 cm path length, excitation wavelength 365 nm).

Beside pyrene, perylene moieties and their derivatives can give rise to excimer emission, and associated CPL in suitable chiral environments.^[21]^ Indeed, moving to compound 3c, containing two 3‐perylenecarboxylate units mounted on isomannide scaffold, the excimer emission was still present. In particular, it could be identified in the fluorescence spectrum, where a large and tailed band at 525 nm is observed (Figure 6, left). This band partially overlaps with the emission of non‐interacting perylene units, as it could be verified taking into account the fluorescence spectra of the corresponding monoester 5c (Figure S32). The excimer emission is associated to a positive CPL band, with a *g_lum_* =2.8 ⋅ 10^−3^, 3.4 times smaller and red‐shifted with respect the one recorded for the pyrene analogous 3a. It is worth mentioning that for derivative 3c, a stacking interaction between the two perylene units in the ground state could be assumed. Indeed this derivative showed an exciton couplet in ECD spectrum centred at 440 nm (Figure S23) and even a significant shift of aromatic proton signals was observed in its ^1^H NMR spectrum, with respect to monoester 5c (Figure S37).

Finally, the mixed derivative 3e, containing both 1‐pyrene carboxylate and 3‐perylene carboxylate units mounted on isomannide scaffold, was studied. Upon excitation at 365 nm, emission and CPL spectra showed only the presence of perylene emission between 400 and 700 nm (λ_max_=493 and 516 nm). This is due to the presence of Forster resonance energy transfer (FRET) effect from pyrene to perylene.^[70]^ On the other hand, no significant CPL signal (Figure S29) associated to the formation of an excited‐state dimer (exciplex, in this case) was observed (Figure S33).^[70]^

In general, thanks to the presence of excimer emission, all the CPL emitting compounds displayed a g_lum_ factor higher than the g_abs_ values measured for red‐shifted transitions. In the case of pyrene‐derivatives, the g_abs_ is in the order of 10^−4^ for both the forbidden S_0_→S_1_ (where observed) and S_0_→S_2_ transition, that is 20–50 times lower than g_lum_. This is in line with what generally observed in the case of CPL associated to pyrene intramolecular excimers.[^19^, ^21^] It is noteworthy that a substantial conformational rearrangement is observed when passing from the ground state S_0_ to the first (emissive) excited state S_1_. In the ground state, the pyrene moieties in the diesters 3 a,b,d lie far apart, and non‐stacked geometries are favoured retaining large flexibility. In the first excited state, intramolecular excimers are prevalently (if not exclusively) obtained, with π‐stacked aromatic rings.

## Conclusion

In conclusion, we have demonstrated that inexpensive compounds, coming from renewable resources, isomannide and isosorbide, can be successfully derivatized with pyrene and perylene moieties. These compounds show interesting (chiro)optical properties. In particular, their CPL can be tuned by choosing the scaffold, the aromatic moiety and the spacer. The possibility to tune CPL emission, and especially to invert the preferential circular polarization of the emitted light, makes isomannide‐pyrene derivatives 3 a–b and 3 d very interesting for further applications, since such derivatives can be easily obtained starting from the same inexpensive chiral scaffold.

The straightforward synthetic approach, requiring one or two synthetic steps, resulted in a simple and economical protocol for obtaining new CPL emitting materials, starting from widely available chiral scaffolds. TD‐DFT calculations were able to reproduce, for the representative pyrene derivative 3 a, the main quantities of CPL spectra, namely the sign, Stokes shift and the order of magnitude of the dissymmetry factor.

## Experimental Section

Experimental procedures, computational details and NMR, UV‐Vis, ECD, fluorescence, CPL spectra are reported in the Supporting Information.

## Conflict of interest

The authors declare no conflict of interest.

1

## Supporting information

As a service to our authors and readers, this journal provides supporting information supplied by the authors. Such materials are peer reviewed and may be re‐organized for online delivery, but are not copy‐edited or typeset. Technical support issues arising from supporting information (other than missing files) should be addressed to the authors.

Supporting Information

## Data Availability

The data that support the findings of this study are available in the supplementary material of this article.

## References

[1] K. Ma , W. Chen , T. Jiao , X. Jin , Y. Sang , D. Yang , J. Zhou , M. Liu , P. Duan , Chem. Sci. 2019, 10, 6821–6827. 31391904 10.1039/c9sc01577a PMC6657416

[2] X. Bai , Y. Sun , Y. Jiang , G. Zhao , J. Jiang , C. Yuan , M. Liu , Angew. Chem. Int. Ed. 2021, 60, 3745–3751. 10.1002/anie.202013550 33170551

[3] H. Nian , L. Cheng , L. Wang , H. Zhang , P. Wang , Y. Li , L. Cao , Angew. Chem. Int. Ed. 2021, 15354–15358. 10.1002/anie.202105593 34111314

[4] X. Bai , Y. Jiang , G. Zhao , J. Jiang , C. Yuan , M. Liu , Soft Matter 2021, 17, 4328–4334. 33908598 10.1039/d1sm00262g

[5] B. Adelizzi , P. Chidchob , N. Tanaka , B. A. G. Lamers , S. C. J. Meskers , S. Ogi , A. R. A. Palmans , S. Yamaguchi , E. W. Meijer , J. Am. Chem. Soc. 2020, 142, 16681–16689. 32880167 10.1021/jacs.0c06921 PMC7530894

[6] M. Louis , R. Sethy , J. Kumar , S. Katao , R. Guillot , T. Nakashima , C. Allain , T. Kawai , R. Métivier , Chem. Sci. 2019, 10, 843–847. 30774879 10.1039/c8sc04026e PMC6345345

[7] M. Schadt , Annu. Rev. Mater. Sci. 1997, 27, 305–379.

[8] Y. Yang , R. C. Da Costa , D. M. Smilgies , A. J. Campbell , M. J. Fuchter , Adv. Mater. 2013, 25, 2624–2628. 23554220 10.1002/adma.201204961 PMC3659407

[9] D. W. Zhang , M. Li , C. F. Chen , Chem. Soc. Rev. 2020, 49, 1331–1343. 31999286 10.1039/c9cs00680j

[10] R. Farshchi , M. Ramsteiner , J. Herfort , A. Tahraoui , H. T. Grahn , Appl. Phys. Lett. 2011, 98, 162508.

[11] J. Yuasa , T. Ohno , H. Tsumatori , R. Shiba , H. Kamikubo , M. Kataoka , Y. Hasegawa , T. Kawai , Chem. Commun. 2013, 49, 4604–4606. 10.1039/c3cc40331a 23525084

[12] R. Hassey , E. J. Swain , N. I. Hammer , D. Venkataraman , M. D. Barnes , Science 2006, 314, 1437–1439. 17082419 10.1126/science.1134231

[13] J. F. Sherson , H. Krauter , R. K. Olsson , B. Julsgaard , K. Hammerer , I. Cirac , E. S. Polzik , Nature 2006, 443, 557–560. 17024089 10.1038/nature05136

[14] Y. Yang , R. C. Da Costa , M. J. Fuchter , A. J. Campbell , Nat. Photonics 2013, 7, 634–638.

[15] S. Furumi , Chem. Rec. 2010, 10, 394–408. 20954194 10.1002/tcr.201000013

[16] J. Jiménez , L. Cerdán , F. Moreno , B. L. Maroto , I. García-Moreno , J. L. Lunkley , G. Muller , S. De La Moya , J. Phys. Chem. C 2017, 121, 5287–5292. 10.1021/acs.jpcc.7b00654 PMC5630177 28993793

[17] J. L. Ma , Q. Peng , C. H. Zhao , Chem. Eur. J. 2019, 25, 15441–15454. 31550061 10.1002/chem.201903252

[18] R. J. Cave , Science 2009, 323, 1435–1436. 19286541 10.1126/science.1169338

[19] L. Arrico , L. Di Bari , F. Zinna , Chem. Eur. J. 2021, 27, 2920–2934. 32725832 10.1002/chem.202002791

[20] H. Tanaka , Y. Inoue , T. Mori , ChemPhotoChem 2018, 2, 386–402.

[21] F. Zinna , E. Brun , A. Homberg , J. Lacour in Circularly Polarizied Luminescence of Isolated Small Organic Molecules (Ed.: T. Mori ), 2020, pp. 273–292.

[22] Y. Mimura , S. Kitamura , M. Shizuma , M. Kitamatsu , M. Fujiki , Y. Imai , ChemistrySelect 2017, 2, 7759–7764.

[23] Y. Mimura , T. Nishikawa , R. Fuchino , S. Nakai , N. Tajima , M. Kitamatsu , M. Fujiki , Y. Imai , Org. Biomol. Chem. 2017, 15, 4548–4553. 28426086 10.1039/c7ob00503b

[24] Y. Mimura , S. Kitamura , M. Shizuma , M. Kitamatsu , Y. Imai , Org. Biomol. Chem. 2018, 16, 6895–6901. 30140836 10.1039/c8ob01869c

[25] T. Nishikawa , S. Kitamura , M. Kitamatsu , M. Fujiki , Y. Imai , ChemistrySelect 2016, 1, 831–835.

[26] Y. Mimura , Y. Motomura , M. Kitamatsu , Y. Imai , Tetrahedron Lett. 2020, 61, 152238.

[27] H. Kashida , K. Nishikawa , Y. Ito , K. Murayama , I. Hayashi , T. Kakuta , T. Ogoshi , H. Asanuma , Chem. Eur. J. 2021, 8510, 1–5. 10.1002/chem.202102333 34472671

[28] K. Nakabayashi , S. Kitamura , N. Suzuki , S. Guo , M. Fujiki , Y. Imai , Eur. J. Org. Chem. 2016, 2016, 64–69.

[29] D. Kaji , S. Ikeda , K. Takamura , N. Tajima , M. Shizuma , T. Mori , M. Miyasaka , Y. Imai , Chem. Lett. 2019, 48, 874–876.

[30] K. Takaishi , R. Takehana , T. Ema , Chem. Commun. 2018, 54, 1449–1452. 10.1039/c7cc09187g 29354827

[31] K. Takaishi , K. Iwachido , T. Ema , J. Am. Chem. Soc. 2020, 142, 1774–1779. 31909994 10.1021/jacs.9b13184

[32] D. Zheng , S. Guo , L. Zheng , Q. Xu , Y. Wang , H. Jiang , Chem. Commun. 2021, 57, 12016–12019. 10.1039/d1cc05163f 34713879

[33] K. Takaishi , S. Murakami , K. Iwachido , T. Ema , Chem. Sci. 2021, 12, 14570–14576. 34881009 10.1039/d1sc04403f PMC8580037

[34] T. Amako , K. Nakabayashi , N. Suzuki , S. Guo , N. A. A. Rahim , T. Harada , M. Fujiki , Y. Imai , Chem. Commun. 2015, 51, 8237–8240. 10.1039/c5cc01465d 25820177

[35] Y. Hashimoto , T. Nakashima , D. Shimizu , T. Kawai , Chem. Commun. 2016, 52, 5171–5174. 10.1039/c6cc01277a 26996611

[36] S. Ito , K. Ikeda , S. Nakanishi , Y. Imai , M. Asami , Chem. Commun. 2017, 53, 6323–6326. 10.1039/c7cc01351e 28497157

[37] N. Hara , M. Shizuma , T. Harada , Y. Imai , RSC Adv. 2020, 10, 11335–11338. 35495310 10.1039/d0ra01552k PMC9050457

[38] P. Reiné , J. Justicia , S. P. Morcillo , S. Abbate , B. Vaz , M. Ribagorda , Á. Orte , L. Álvarez De Cienfuegos , G. Longhi , A. G. Campaña , et al., J. Org. Chem. 2018, 83, 4455–4463. 29577727 10.1021/acs.joc.8b00162 PMC6145600

[39] D. Zheng , S. Guo , L. Zheng , Q. Xu , Y. Wang , H. Jiang , Chem. Commun. 2021, 57, 12016–12019. 10.1039/d1cc05163f 34713879

[40] A. Homberg , E. Brun , F. Zinna , S. Pascal , M. Górecki , L. Monnier , C. Besnard , G. Pescitelli , L. Di Bari , J. Lacour , Chem. Sci. 2018, 9, 7043–7052. 30310624 10.1039/c8sc02935k PMC6137439

[41] U. Koert , e-EROS Encycl. Reagents Org. 2012, 1–5.

[42] M. Kadraoui , T. Maunoury , Z. Derriche , S. Guillarme , C. Saluzzo , Eur. J. Org. Chem. 2015, 2015, 441–457.

[43] L. Y. Chen , S. Guillarme , C. Saluzzo , Arkivoc 2013, 2013, 227–244.

[44] A. Marra , C. Chiappe , A. Mele , Chimia 2011, 65, 76–80. 21469450 10.2533/chimia.2011.76

[45] V. Zullo , A. Iuliano , L. Guazzelli , Molecules 2021, 26, 2052. 33916695 10.3390/molecules26072052 PMC8038380

[46] D. G. Blackmond , T. Rosner , T. Neugebauer , M. T. Reetz , Angew. Chem. Int. Ed. 1999, 38, 2196–2199. 10.1002/(sici)1521-3773(19990802)38:15<2196::aid-anie2196>3.0.co;2-9 10425479

[47] M. T. Reetz , G. Mehler , Angew. Chem. Int. Ed. 2000, 39, 3889–3890. 10.1002/1521-3773(20001103)39:21<3889::AID-ANIE3889>3.0.CO;2-T 29711714

[48] M. T. Reetz , T. Neugebauer , Angew. Chem. Int. Ed. 1999, 38, 179–181.

[49] V. Zullo , M. Górecki , L. Guazzelli , A. Mezzetta , G. Pescitelli , A. Iuliano , J. Mol. Liq. 2021, 322, 114528.

[50] H. H. Jaffe, M. Orchin, *Theory and Applications of Ultraviolet Spectroscopy*, Wiley, New York, **1962**.

[51] I. S. K. Kerkines , I. D. Petsalakis , G. Theodorakopoulos , W. Klopper , J. Chem. Phys. 2009, 131, 224315. 20001044 10.1063/1.3271347

[52] O. a. Khakhel , J. Appl. Spectrosc. 2001, 68, 280–286.

[53] C. Armbruster , M. Knapp , K. Rechthaler , R. Schamschule , A. B. J. Parusel , G. Köhler , W. Wehrmann , J. Photochem. Photobiol. A 1999, 125, 29–38.

[54] Z. Mahmood , J. Zhao , J. Org. Chem. 2016, 81, 587–594. 26694534 10.1021/acs.joc.5b02415

[55] K. Tsubaki , H. Tanaka , K. Takaishi , M. Miura , H. Morikawa , T. Furuta , K. Tanaka , K. Fuji , T. Sasamori , N. Tokitoh , et al., J. Org. Chem. 2006, 71, 6579–6587. 16901146 10.1021/jo060974v

[56] I. Trkulja , R. Häner , J. Am. Chem. Soc. 2007, 129, 7982–7989. 17542587 10.1021/ja0715501

[57] H. Langhals , A. Hofer , S. Bernhard , J. S. Siegel , P. Mayer , J. Org. Chem. 2011, 76, 990–992. 21229974 10.1021/jo102254a

[58] J. Kumar , T. Nakashima , H. Tsumatori , M. Mori , M. Naito , T. Kawai , Chem. Eur. J. 2013, 19, 14090–14097. 24026812 10.1002/chem.201302146

[59] M. J. Frisch, G. W. Trucks, H. B. Schlegel, G. E. Scuseria, M. A. Robb, J. R. Cheeseman, G. Scalmani, V. Barone, G. A. Petersson, H. Nakatsuji, et al., **2016**.

[60] M. Parac , S. Grimme , Chem. Phys. 2003, 292, 11–21. 10.1002/cphc.200390047 12674603

[61] A. G. Crawford , A. D. Dwyer , Z. Liu , A. Steffen , A. Beeby , L.-O. Pålsson , D. J. Tozer , T. B. Marder , J. Am. Chem. Soc. 2011, 133, 13349–13362. 21751803 10.1021/ja2006862

[62] M. Pecul , K. Ruud , Phys. Chem. Chem. Phys. 2011, 13, 643–650. 21031208 10.1039/c0cp01149e

[63] G. Longhi , E. Castiglioni , S. Abbate , F. Lebon , D. A. Lightner , Chirality 2013, 25, 589–599. 23840012 10.1002/chir.22176

[64] G. Longhi , E. Castiglioni , J. Koshoubu , G. Mazzeo , S. Abbate , Chirality 2016, 28, 696–707. 27670249 10.1002/chir.22647

[65] R. Huenerbein , S. Grimme , Chem. Phys. 2008, 343, 362–371.

[66] M. Kołaski , C. R. Arunkumar , K. S. Kim , J. Chem. Theory Comput. 2013, 9, 847–856. 26589075 10.1021/ct300350m

[67] C. Benkhauser-Schunk , B. Wezisla , K. Urbahn , U. Kiehne , J. Daniels , G. Schnakenburg , F. Neese , A. Lützen , ChemPlusChem 2012, 77, 396–403.

[68] F. Neese , WIREs Comput. Mol. Sci. 2018, 8, e1327.

[69] F. Neese , F. Wennmohs , U. Becker , C. Riplinger , J. Chem. Phys. 2020, 152, 224108. 32534543 10.1063/5.0004608

[70] K. Takaishi , S. Murakami , K. Iwachido , T. Ema , Chem. Sci. 2021, 12, 14570–14576. 34881009 10.1039/d1sc04403f PMC8580037

