# Supplementary material for: Tunable Excimer Circularly Polarized Luminescence in Isohexide Derivatives from Renewable Resources
Source: Chemistry. 2022 Feb 15;28(15):e202104226. doi: 10.1002/chem.202104226 (PMC9303411; doi:10.1002/chem.202104226)
Supplement: Supplementary file 1 — Supporting Information [file CHEM-28-0-s001.pdf]

Chemistry–A European Journal

Supporting Information

Tunable Excimer Circularly Polarized Luminescence in Isohexide Derivatives from Renewable Resources

Valerio Zullo,* Anna Iuliano, Gennaro Pescitelli, and Francesco Zinna*

Table of Contents

1 General I

2 Synthesis of derivatives 3-7 II

2.1 Representative esterification procedure II

2.2 (3R,3aR,6R,6aR)-hexahydrofuro[3,2-b]furan-3,6-di-1-(pyrenyl)carboxylate, 3a II

2.3 (3R,3aR,6S,6aR)-hexahydrofuro[3,2-b]furan-3,6-di-(1-pyrenyl)carboxylate, 4a IV

2.4 (3R,3aR,6R,6aR)-hexahydrofuro[3,2-b]furan-3,6-di- (1-pyrenyl)acetate, 3b VI

2.5 (3R,3aR,6S,6aR)-hexahydrofuro[3,2-b]furan-3,6-di-(1-pyrenyl)acetate, 4b VIII

2.6 (3R,3aR,6R,6aR)-hexahydrofuro[3,2-b]furan-3,6-di-(3-perylenyl)carboxylate, 3c X

2.7 (3R,3aR,6R,6aR)-hexahydrofuro[3,2-b]furan-3-(1-pyrenyl)carboxylate-6-ol, 5a XII

2.8 (3R,3aR,6R,6aR)-hexahydrofuro[3,2-b]furan-3-(1-pyrenyl)acetate-6-ol, 5b XIV

2.9 (3R,3aR,6R,6aR)-hexahydrofuro[3,2-b]furan-3-(3-perylenyl)carboxylate-6-ol, 5c XVI

2.10 (3R,3aR,6R,6aR)-hexahydrofuro[3,2-b]furan-3-(1-pyrenyl)carboxylate-6-(1-pyrenyl)acetate, 3d XVIII

2.11 (3R,3aR,6R,6aR)-hexahydrofuro[3,2-b]furan-3-(1-pyrenyl)carboxylate-6-(3-perylenyl)carboxylate, 3e XX

2.12 3-acetylperylene, 6[2] XXII

2.13 3-perylencarboxylic acid, 7[3] XXIII

3 UV-Vis and ECD spectra of compounds 3, 4 and 5. XXV

3.1 (3R,3aR,6R,6aR)-hexahydrofuro[3,2-b]furan-3,6-di-(3-perylenyl)carboxylate, 3c XXV

3.2 (3R,3aR,6R,6aR)-hexahydrofuro[3,2-b]furan-3-(1-pyrenyl)carboxylate-6-(1-pyrenyl)acetate, 3d XXV

3.3 (3R,3aR,6R,6aR)-hexahydrofuro[3,2-b]furan-3-(1-pyrenyl)carboxylate-6-(3-perylenyl)carboxylate, 3e XXVI

3.4 (3R,3aR,6R,6aR)-hexahydrofuro[3,2-b]furan-3-(1-pyrenyl)carboxylate-6-ol, 5a XXVI

3.5 (3R,3aR,6R,6aR)-hexahydrofuro[3,2-b]furan-3-(1-pyrenyl)acetate-6-ol, 5b XXVII

3.6 (3R,3aR,6R,6aR)-hexahydrofuro[3,2-b]furan-3-(3-perylenyl)carboxylate-6-ol, 5c XXVII

4 Circularly polarized luminescence (CPL) spectra of compounds 3e and 5. XXVIII

4.1 (3R,3aR,6R,6aR)-hexahydrofuro[3,2-b]furan-3-(1-pyrenyl)carboxylate-6-(3-perylenyl)carboxylate, 3e XXVIII

4.2 (3R,3aR,6R,6aR)-hexahydrofuro[3,2-b]furan-3-(1-pyrenyl)carboxylate-6-ol, 5a XXVIII

4.3 (3R,3aR,6R,6aR)-hexahydrofuro[3,2-b]furan-3-(1-pyrenyl)acetate-6-ol, 5b XXIX

4.4 (3R,3aR,6R,6aR)-hexahydrofuro[3,2-b]furan-3-(3-perylenyl)carboxylate-6-ol, 5c XXIX

4.5 Superimposition of fluorescence spectra XXX

4.6 ^{1}H NMR comparison between monoester 5 and diesters 3 XXXI

5 Computational details XXXV

5.1 Compound 3a XXXV

5.2 Compound 3d XLIII

5.3 Chromophoric models XLVII

6 References XLIX

1 General

All the reactions involving sensitive compounds were carried out under dry Ar, in flame-dried glassware. If not noted otherwise, reactants and reagents were commercially available and used as received from Alfa Aesar, Fluorochem, TCI-Chemicals and Sigma-Aldrich. Dichloromethane and tetrahydrofuran were dried and degassed by MB-SPS solvent purification system. Analytical TLC was performed on precoated silica gel ALUGRAM Xtra G/UV254 plates. Purifications were performed by Biotage® Isolera chromatograph employing Silica gel cartridges. Melting points were measured on a Reichert Thermovar Type 300429 Microscope.^{1}H NMR spectra were recorded in $CDCl_3$ or in DMSO-d_6 on a Bruker 400MHz NMR spectrometer or on a JEOL ECZ 400S spectrometer. The following abbreviations are used: s=singlet, d=doublet, dd=double doublet, dt=double triplet, t=triplet, tdt=triple double triplet, q=quartet, m=multiplet. ^{13}C NMR spectra were recorded at 101 MHz. ^{1}H and ^{13}C NMR chemical shifts (ppm) are referred to TMS as external standard. Elemental analyses were obtained using an Elementar Vario MICRO cube equipment. ECD/UV spectra were recorded using a J-715 spectrometer (Jasco, Tokyo, Japan) at room temperature in spectroscopic grade solvents. Solutions with suitable concentrations (details are given in the legend of each figure) were measured in quartz cells with a path length 1 cm. All spectra were recorded using a scanning speed of 100 nm/min, a step size of 0.2 nm, a bandwidth of 1 nm, a response time of 0.5 s, and an accumulation of 8 scans. The spectra were background-corrected using the respective solvent spectra recorded under the same conditions.CPL/Fluorescence spectra were recorded with a home-built apparatus[1] by exciting the samples with a 365 nm LED source and 90° detection geometry. To exclude artefacts due to linear anisotropy, the samples were excited with light linearly polarized on the detection plane.

2 Synthesis of derivatives 3-7

2.1 Representative esterification procedure

Under an Ar atmosphere, EDC·HCl and DMAP were added at 0 °C to a dispersion of the alcohol and carboxylic acid in dry solvent (CH_2Cl_2 or THF). The mixture was stirred at room temperature and the reaction was monitored by TLC analysis ($CHCl_3$:MeOH 9:1 or CH_2Cl_2:Acetone 9:1). The reaction was stopped after 24 hours. The crude was processed as described in the following sections.

2.2 (3R,3aR,6R,6aR)-hexahydrofuro[3,2-b]furan-3,6-di-1-(pyrenyl)carboxylate, 3a

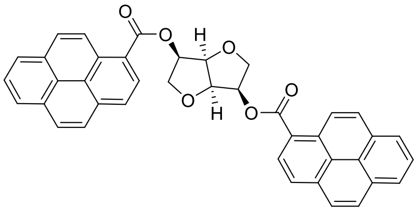

Isomannide (**1**, 58.7 mg, 0.40 mmol), EDC·HCl (190.7 mg, 0.99 mmol), DMAP (27.0 mg, 0.22 mmol), dry CH_2Cl_2 (3 mL), 1-pyrenecarboxylic acid (226.2 mg, 0.92 mmol), TLC ($CHCl_3$:MeOH 9:1). The product, crystallized as a light yellow solid, was filtered to give 100.6mg (0.17 mmol, 42%) of pure product. The filtrate was diluted with CH_2Cl_2 (15mL), washed with H_2O (15 mL) and dried over anhydrous Na_2SO_4. The solvent was removed under reduced pressure and the crude was purified by recrystallization from CH_2Cl_2/MeOH, to give the pure product as a light-yellow solid (93.8 mg, 0.16mmol; 39%). The total yield of the pure product was 81%.

m.p. 179-181°C

^{1}H NMR (401 MHz, Chloroform-*d*) δ 9.31 (d, *J* = 9.5 Hz, 2H), 8.71 (d, *J* = 8.1 Hz, 2H), 8.30 – 8.21 (m, 5H), 8.15 (dd, *J* = 13.6, 8.5 Hz, 5H), 8.09 – 8.02 (m, 4H), 5.68 – 5.56 (m, 2H), 5.19 – 5.07 (m, 2H), 4.40 – 4.20 (m, 4H).

^{13}C NMR (101 MHz, Chloroform-*d*) δ 167.5, 134.7, 131.5, 131.1, 130.5, 129.9, 129.8, 128.8, 127.3, 126.5, 126.5, 126.4, 125.0, 124.9, 124.3, 122.8, 81.2, 74.6, 71.3.

Elemental analysis Calcd for $C_{40}H_{26}O_6$: C, 79.72; H, 4.35; O, 15.93; Found: C, 79.43; H, 4.51.

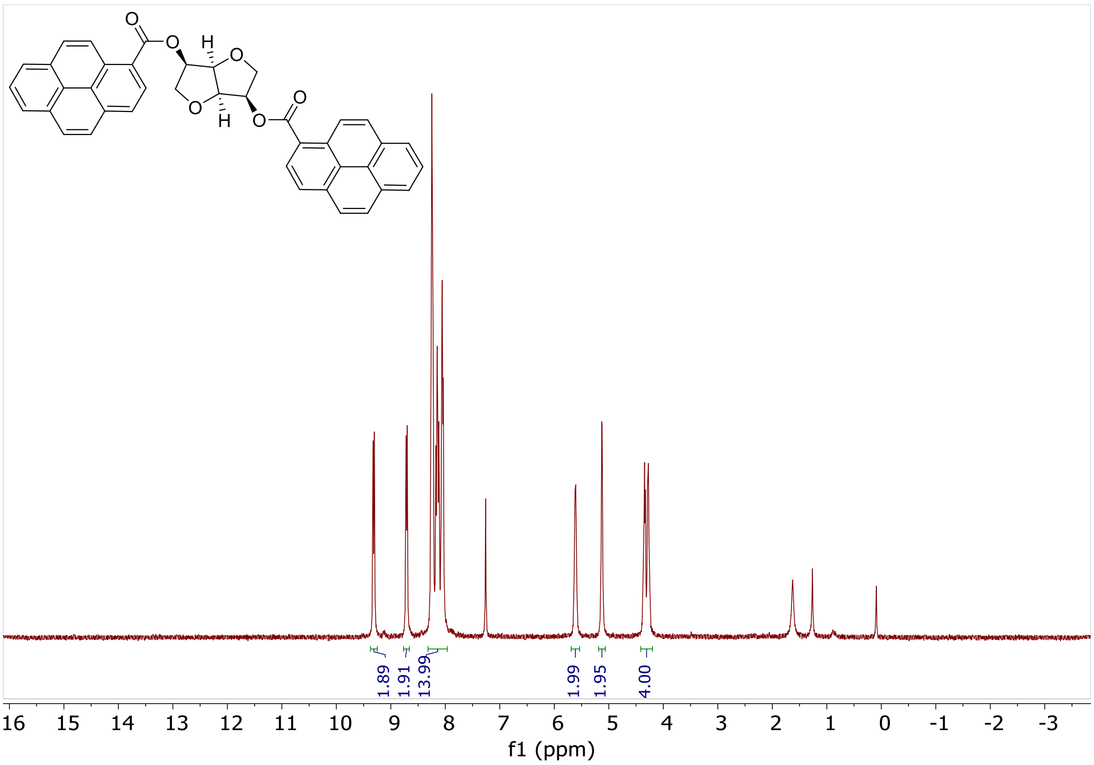

Figure S1. ^{1}H NMR (401 MHz, Chloroform-*d*) spectrum of compound **3a**

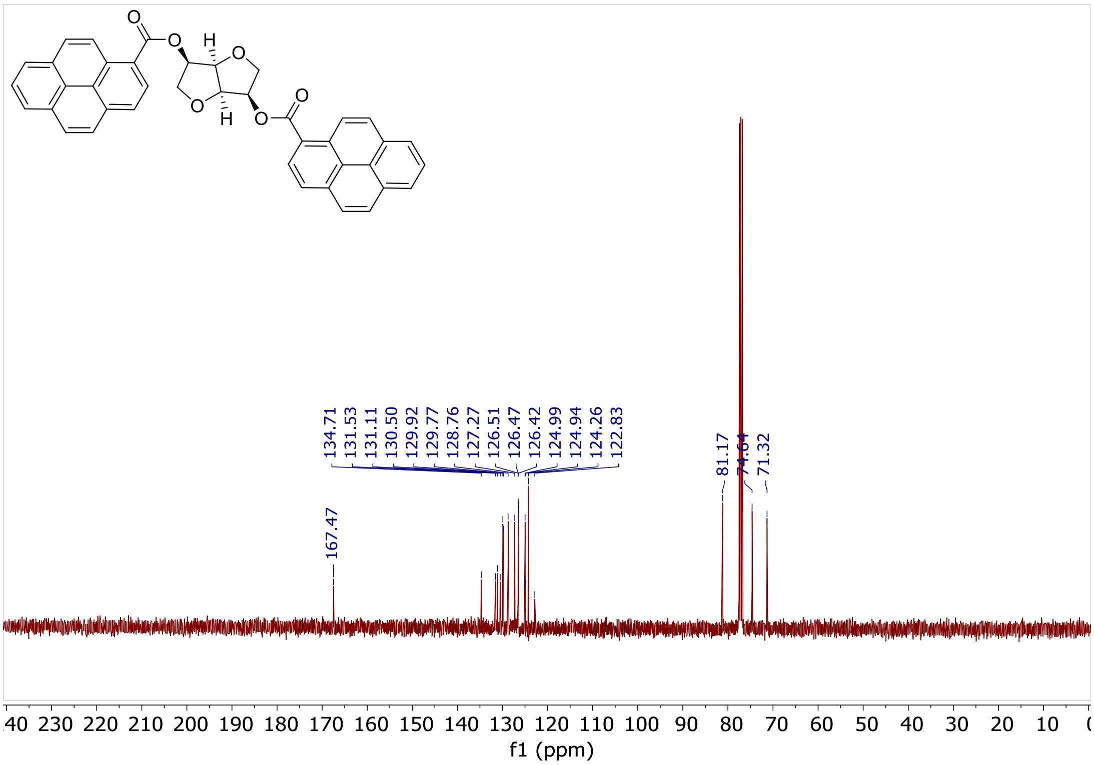

Figure S2. ^{13}C NMR (101 MHz, Chloroform-*d*) spectrum of compound **3a**

2.3 (3R,3aR,6S,6aR)-hexahydrofuro[3,2-b]furan-3,6-di-(1-pyrenyl)carboxylate, 4a

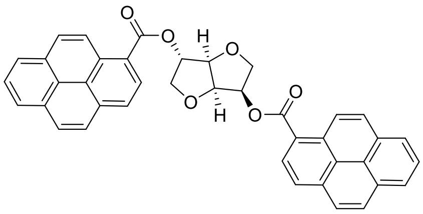

Isosorbide (**2**, 65.7 mg, 0.45 mmol), EDC·HCl (220.5 mg, 1.2 mmol), DMAP (27.5 mg, 0.23 mmol), dry CH_2Cl_2 (3 mL), 1-pyrenecarboxylic acid (257.0 mg, 1.04 mmol), TLC ($CHCl_3$:MeOH 9:1). The crude product, crystallized as a yellow solid, was filtered to give 136 mg of product. The filtrate was diluted with CH_2Cl_2 (15mL), washed with H_2O (15 mL) and dried over anhydrous Na_2SO_4. The solvent was removed under reduced pressure to give 178 mg of crude product as a yellow glassy solid. The solids were reunited and purified by recrystallization from CH_2Cl_2/MeOH, to give the pure product as a yellow solid (178.4 mg, 0.30 mmol; 66%).

m.p. 104-106°C

^{1}H NMR (401 MHz, Chloroform-*d*) δ 9.34 (d, *J* = 9.4 Hz, 1H), 9.26 (d, *J* = 9.4 Hz, 1H), 8.73 (d, *J* = 8.1 Hz, 1H), 8.63 (d, *J* = 8.2 Hz, 1H), 8.35 – 7.98 (m, 14H), 5.77 – 5.72 (m, 1H), 5.68 (q, *J* = 5.5 Hz, 1H), 5.31 (t, *J* = 5.1 Hz, 1H), 4.93 (d, *J* = 4.7 Hz, 1H), 4.35 (d, *J* = 2.4 Hz, 2H), 4.32 – 4.21 (m, 2H).

^{13}C NMR (101 MHz, Chloroform-*d*) δ 167.4, 167.0, 134.8, 134.8, 131.6, 131.1, 131.1, 130.5, 130.5, 130.1, 130.0, 129.8, 128.7, 128.7, 127.3, 127.3, 126.6, 126.5, 126.5, 125.0, 124.8, 124.3, 124.2, 122.5, 86.6, 81.6, 79.0, 75.0, 73.9, 71.1.

Elemental analysis Calcd for $C_{40}H_{26}O_6$: C, 79.72; H, 4.35; O, 15.93; Found: C, 79.52; H, 4.40.

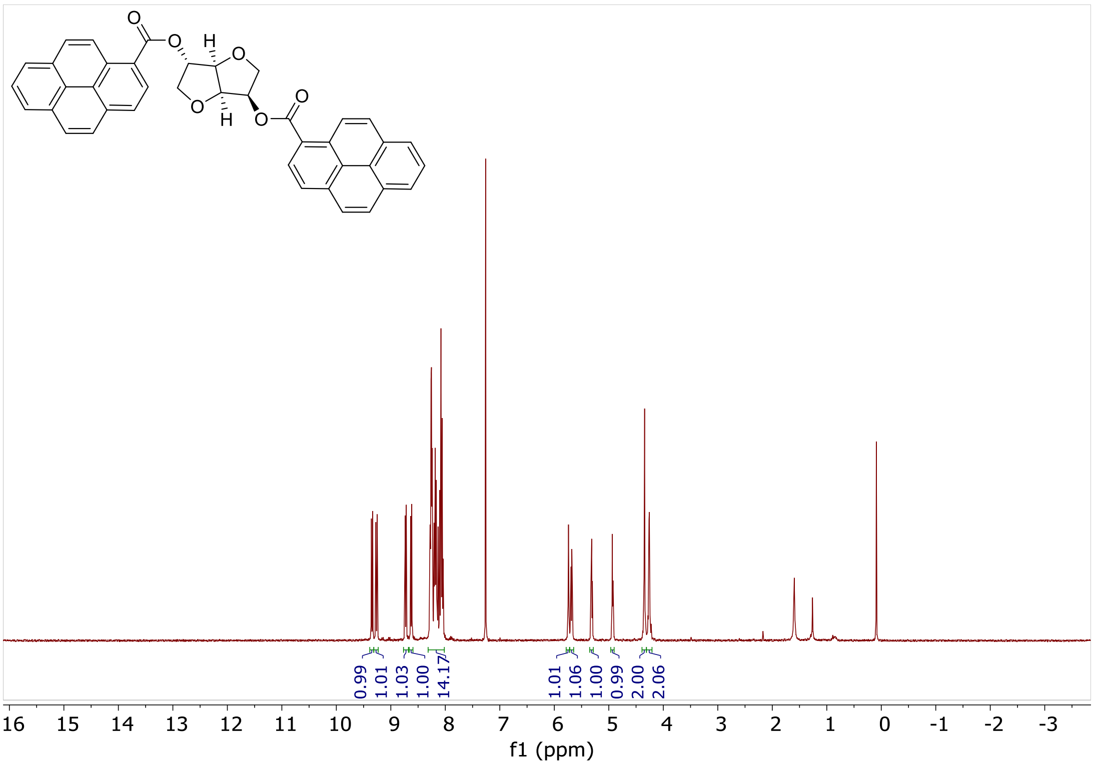

Figure S3. ^{1}H NMR (401 MHz, Chloroform-*d*) spectrum of compound **4a**

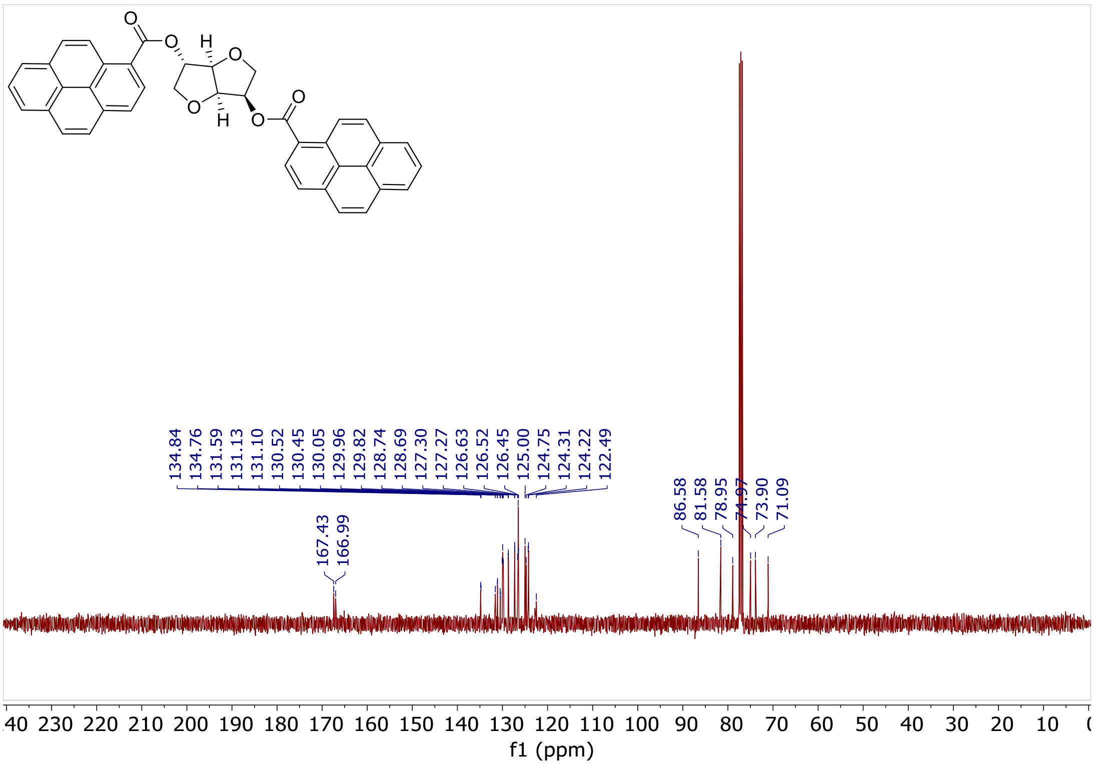

Figure S4. ^{13}C NMR (101 MHz, Chloroform-*d*) spectrum of compound **4a**

2.4 (3R,3aR,6R,6aR)-hexahydrofuro[3,2-b]furan-3,6-di- (1-pyrenyl)acetate, 3b

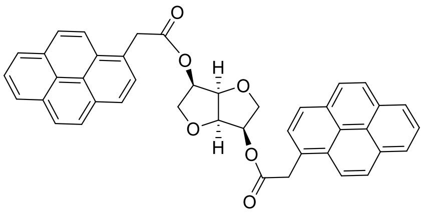

Isomannide (**1**, 55.0 mg, 0.38 mmol), EDC·HCl (194.6 mg, 0.90 mmol), DMAP (21.7 mg, 0.18 mmol), dry CH_2Cl_2 (3 mL), 1-pyrenylacetic acid (235.1 mg, 0.90 mmol), TLC ($CHCl_3$:MeOH 9:1). The reaction mixture was diluted with CH_2Cl_2 (30 mL), washed with H_2O (3x10 mL) and dried over anhydrous Na_2SO_4. The solvent was removed under reduced pressure and the crude was purified by recrystallization from CH_2Cl_2/MeOH, to give the pure product as a light-yellow solid (172.1 mg, 0.27 mmol; 72%).

m.p. 167-169°C

^{1}H NMR (401 MHz, Chloroform-*d*) δ 8.25 – 8.15 (m, 6H), 8.14 – 8.09 (m, 4H), 8.09 – 7.98 (m, 6H), 7.91 (d, *J* = 7.8 Hz, 2H), 5.02 (tdt, *J* = 5.6, 3.8, 1.6 Hz, 2H), 4.67 – 4.61 (m, 2H), 4.35 (s, 4H), 3.77 (dd, *J* = 9.5, 6.5 Hz, 2H), 3.56 (dd, *J* = 9.5, 6.8 Hz, 2H).

^{13}C NMR (101 MHz, Chloroform-*d*) δ 171.0, 131.4, 131.0, 130.9, 129.6, 128.5, 128.0, 127.8, 127.5, 127.5, 126.2, 125.4, 125.3, 125.2, 125.0, 124.9, 123.4, 80.3, 74.2, 70.5, 39.1.

Elemental analysis Calcd for $C_{42}H_{30}O_6$: C, 79.98; H, 4.79; O, 15.22; Found: C, 79.86; H, 4.93.

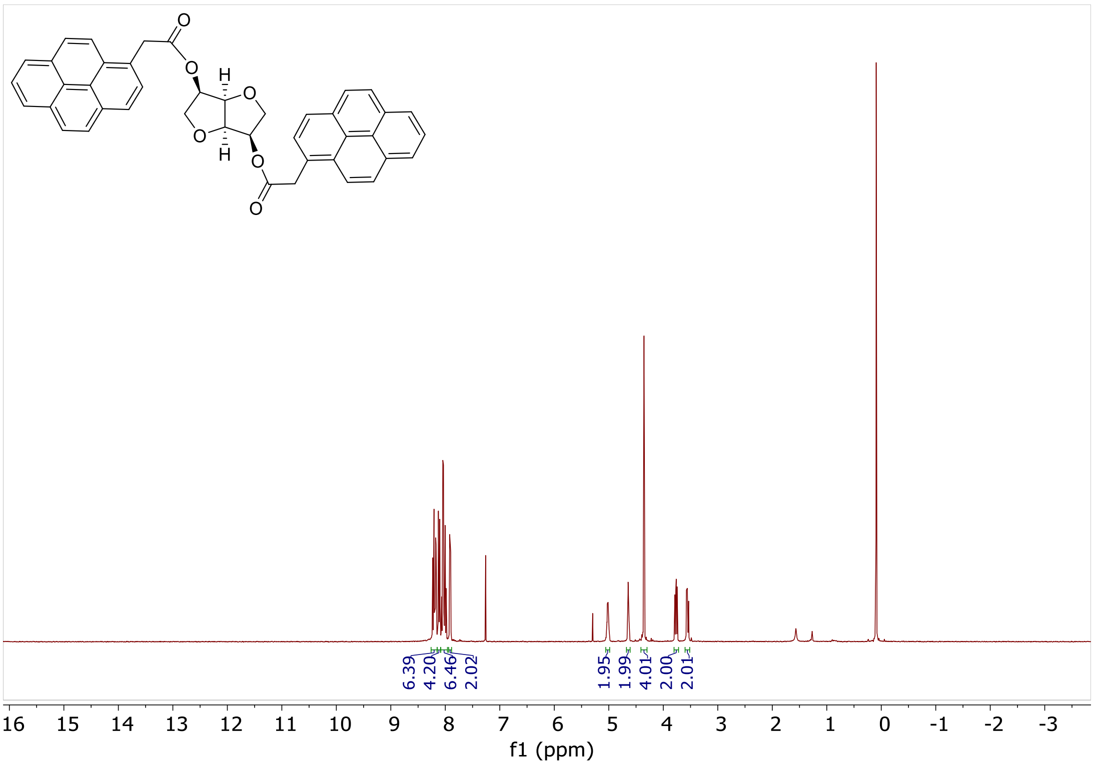

Figure S5. ^{1}H NMR (401 MHz, Chloroform-*d*) spectrum of compound **3b**.

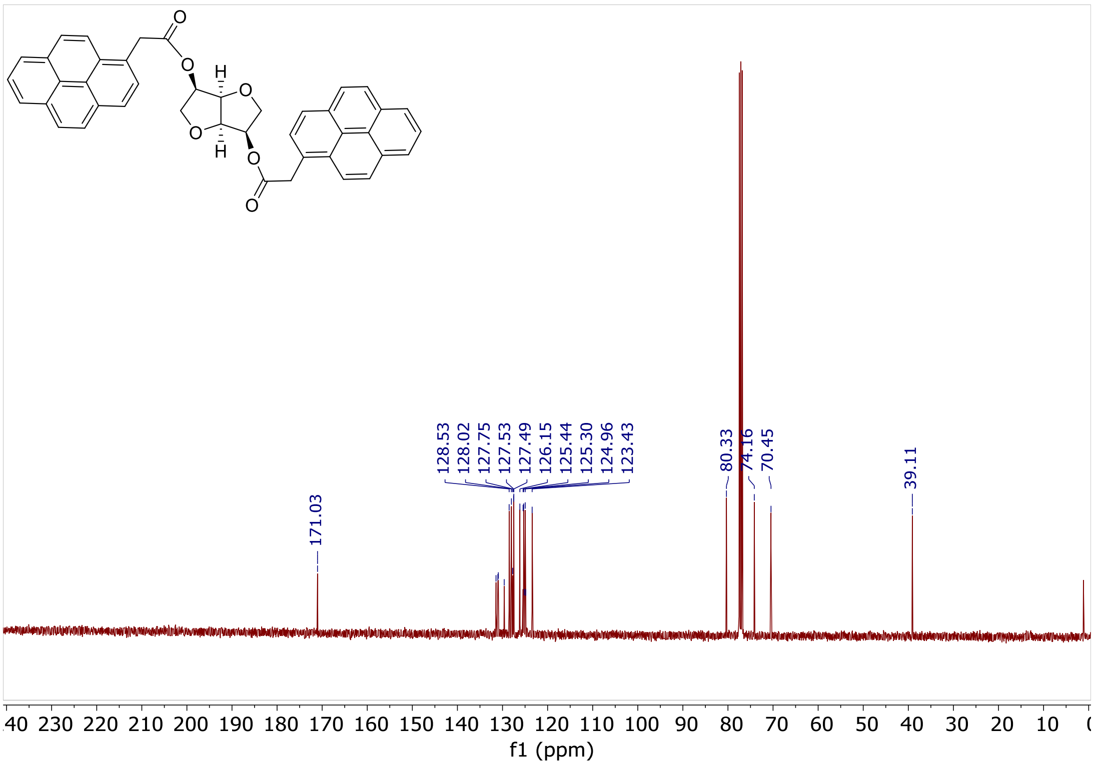

Figure S6. ^{13}C NMR (101 MHz, Chloroform-*d*) spectrum of compound **3b**

2.5 (3R,3aR,6S,6aR)-hexahydrofuro[3,2-b]furan-3,6-di-(1-pyrenyl)acetate, 4b

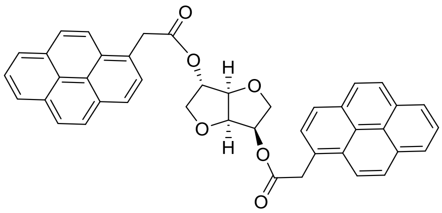

Isosorbide (**2**, 54.6 mg, 0.37 mmol), EDC·HCl (182.2 mg, 0.95 mmol), DMAP (21.7 mg, 0.18 mmol), dry CH_2Cl_2 (3 mL), 1-pyrenylacetic acid (230.1 mg, 0.88 mmol), TLC ($CHCl_3$:MeOH 9:1). The reaction mixture was diluted with CH_2Cl_2 (30 mL), washed with H_2O (3x10 mL) and dried over anhydrous Na_2SO_4. The solvent was removed under reduced pressure and the crude was purified by recrystallization from CH_2Cl_2/MeOH, to give the pure product as a light-yellow solid (154.5 mg, 0.24 mmol; 66%).

m.p. 127-130°C

^{1}H NMR (401 MHz, Chloroform-*d*) δ 8.25 (d, *J* = 9.3 Hz, 1H), 8.22 – 8.16 (m, 5H), 8.12 (dd, *J* = 8.5, 5.8 Hz, 4H), 8.09 – 7.98 (m, 6H), 7.94 (d, *J* = 7.8 Hz, 1H), 7.88 (d, *J* = 7.8 Hz, 1H), 5.18 – 5.07 (m, 2H), 4.75 (t, *J* = 5.2 Hz, 1H), 4.39 (s, 2H), 4.30 (s, 2H), 4.27 (d, *J* = 4.8 Hz, 1H), 3.83 – 3.72 (m, 2H), 3.68 (d, *J* = 10.7 Hz, 1H), 3.60 (dd, *J* = 10.7, 3.4 Hz, 1H).

^{13}C NMR (101 MHz, Chloroform-*d*) δ 171.0, 170.7, 131.4, 131.1, 131.0, 130.9, 130.9, 129.6, 129.5, 128.6, 128.4, 128.2, 128.1, 127.7, 127.6, 127.5, 127.5, 126.2, 126.1, 125.5, 125.4, 125.4, 125.3, 125.2, 125.1, 125.0, 125.0, 124.8, 123.4, 123.1, 86.0, 80.9, 78.5, 74.5, 73.1, 70.8, 39.4, 39.3.

Elemental analysis Calcd for $C_{42}H_{30}O_6$: C, 79.98; H, 4.79; O, 15.22; Found: C, 79.87; H, 4.96.

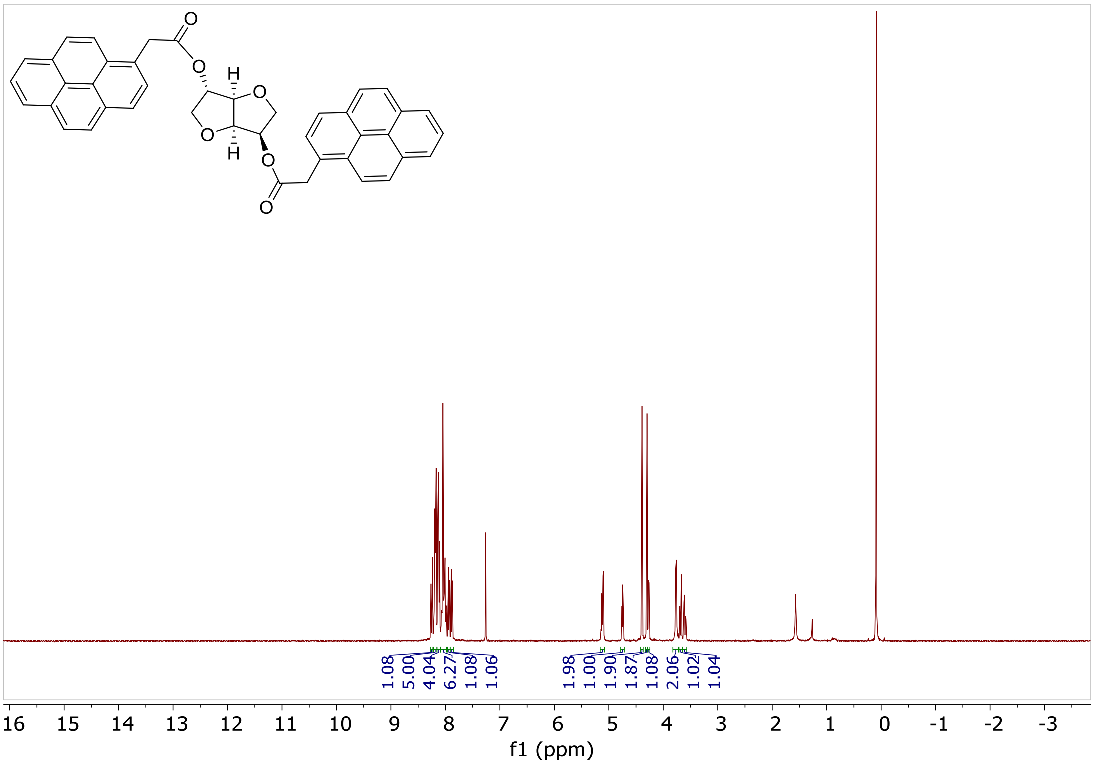

Figure S7. ^{1}H NMR (401 MHz, Chloroform-*d*) spectrum of compound **4b**

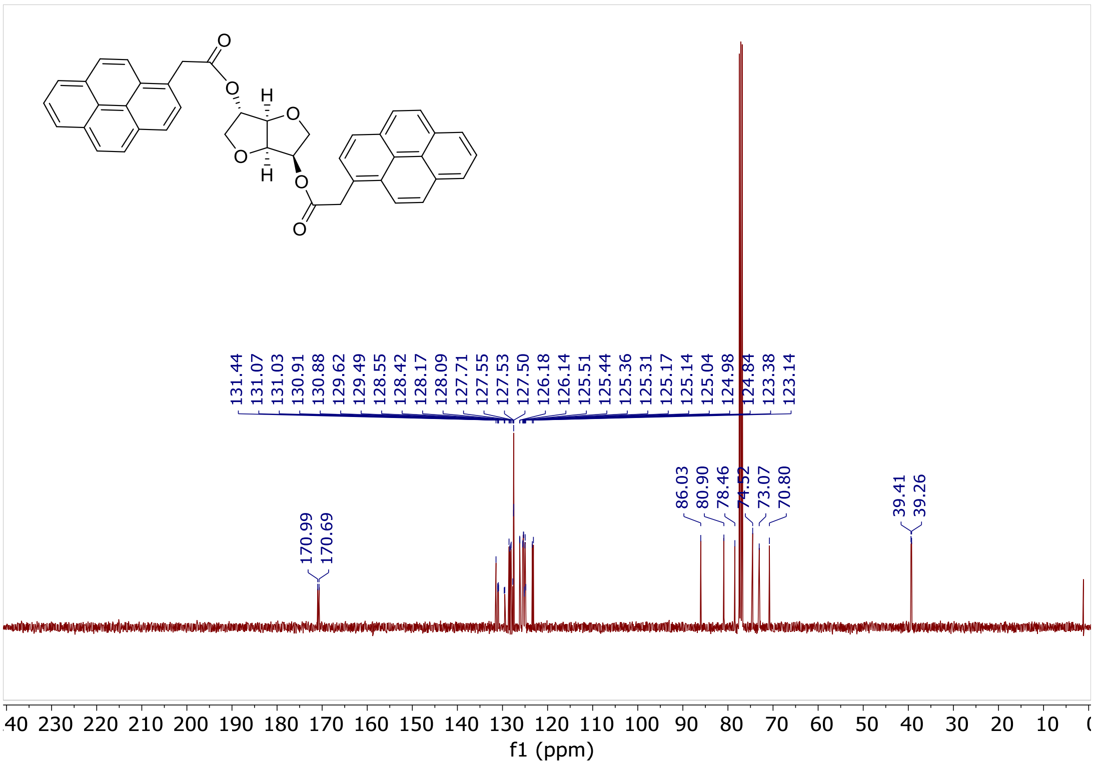

Figure S8. ^{13}C NMR (101 MHz, Chloroform-*d*) spectrum of compound **4b**

2.6 (3R,3aR,6R,6aR)-hexahydrofuro[3,2-b]furan-3,6-di-(3-perylenyl)carboxylate, 3c

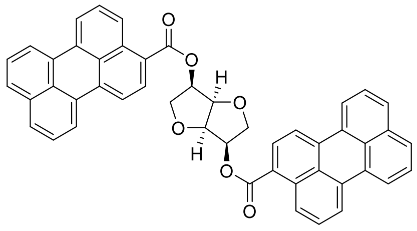

Isomannide (**1**, 27.9 mg, 0.19 mmol), EDC·HCl (114.9 mg, 0.60 mmol), DMAP (17.8 mg, 0.15 mmol), dry CH_2Cl_2 (2 mL), 3-perylencarboxylic acid (**7**, 143.3 mg, 0.48 mmol), TLC (CH_2Cl_2:Acetone 9:1). The reaction was stopped after 21 hours. The reaction mixture was diluted with $CHCl_3$ (30mL), washed with H_2O (3x10 mL) and dried over anhydrous Na_2SO_4. The solvent was removed under reduced pressure and the crude was purified by Biotage® Isolera chromatograph ($CHCl_3$:Acetone) to give the pure product as an orange solid (38.7 mg, 0.005 mmol, 28%).

m.p. 269-271°C

^{1}H NMR (401 MHz, DMSO-d_6) δ 8.73 (d, J = 8.6 Hz, 2H), 8.53 – 8.27 (m, 8H), 8.16 (d, J = 8.0 Hz, 2H), 7.87 (dd, J = 14.6, 8.1 Hz, 4H), 7.70 (t, J = 8.1 Hz, 2H), 7.56 (dt, J = 20.7, 7.8 Hz, 4H), 5.53 – 5.38 (m, 2H), 5.04 – 4.90 (m, 2H), 4.21 (dd, J = 9.7, 5.7 Hz, 2H), 4.10 (dd, J = 9.7, 5.4 Hz, 2H).

^{13}C NMR (101 MHz, DMSO-d_6) δ 166.0, 135.2, 134.0, 132.2, 130.8, 130.8, 130.1, 129.6, 128.5, 128.3, 127.4, 127.2, 127.0, 125.4, 125.1, 122.6, 121.8, 121.2, 119.6, 80.7, 74.0, 71.3.

Elemental analysis Calcd for $C_{49}H_{34}O_6$: C, 81.88; H, 4.77; O, 13.35; Found: C, 81.72; H, 4.85.

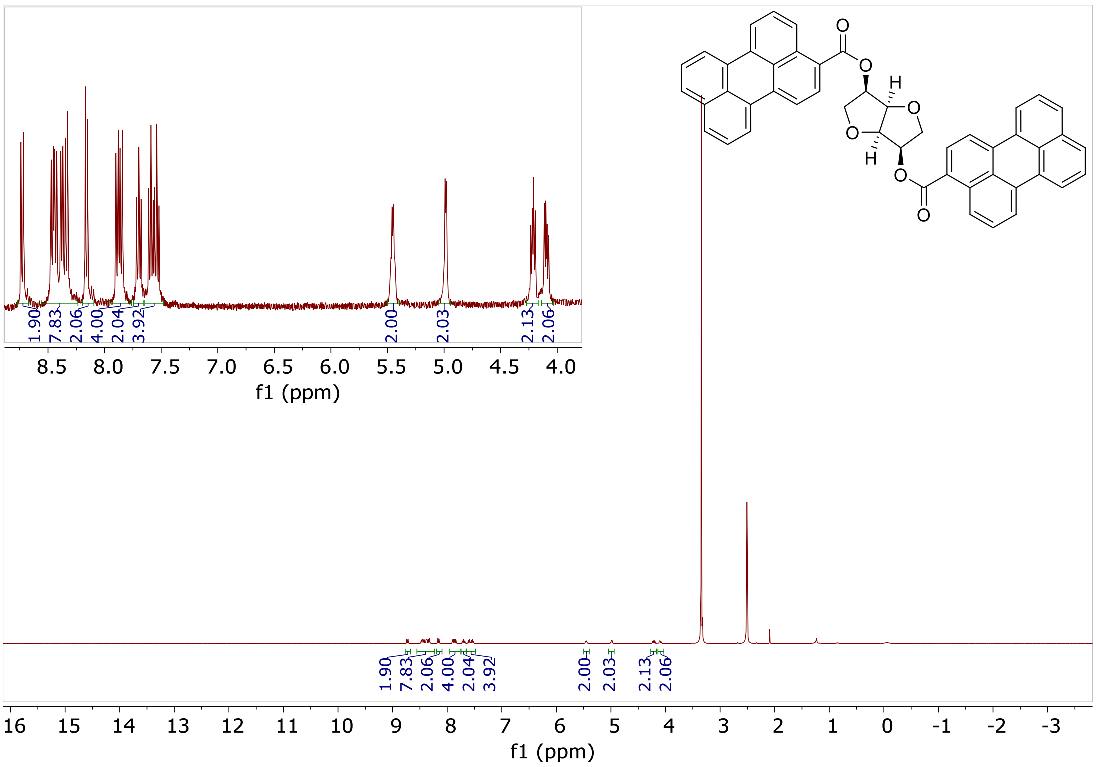

Figure S9. ^{1}H NMR (401 MHz, DMSO-d_6) spectrum of compound **3c**

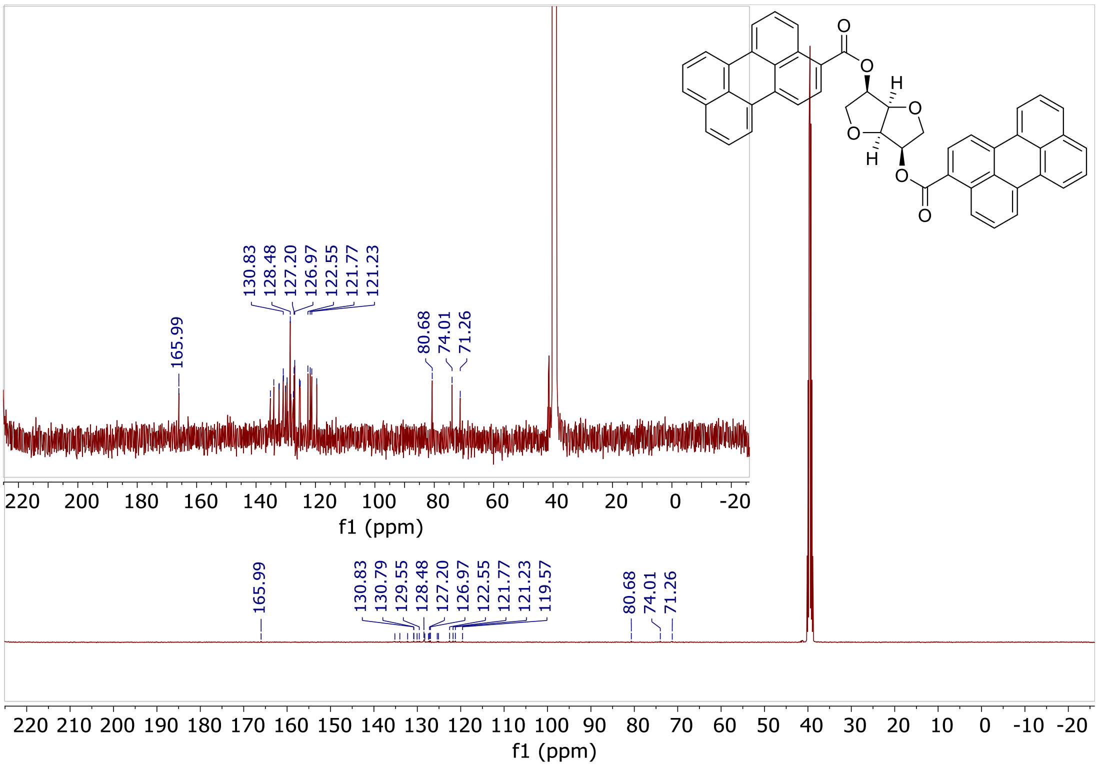

Figure S10. ^{13}C NMR (101 MHz, DMSO-d_6) spectrum of compound **3c**

2.7 (3R,3aR,6R,6aR)-hexahydrofuro[3,2-b]furan-3-(1-pyrenyl)carboxylate-6-ol, 5a

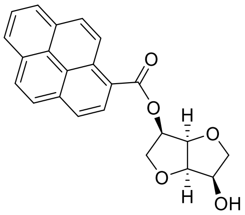

To a solution of isomannide (**1**, 218.4 mg, 1.49 mmol), EDC·HCl (132.4 mg, 0.69 mmol) and DMAP (18.4 mg, 0.15 mmol) in dry THF (20 mL), 1-pyrenecarboxylic acid (124.3 mg, 0.50 mmol) was added and the reaction mixture was stirred at RT for 24 hours, monitoring by TLC (CH_2Cl_2:Acetone 9:1). The solvent was removed under reduced pressure, the crude was dissolved in CH_2Cl_2 (20 mL) and the organic phase was washed with water (3x10 mL) and dried over anhydrous Na_2SO_4. The solvent was removed under the reduced pressure and the crude was purified by Biotage® Isolera chromatograph (CH_2Cl_2:Acetone) to give the pure product **5a** as a yellow solid (77.6 mg, 0.21 mmol, 41%).

m.p. 153-155°C

^{1}H NMR (401 MHz, Chloroform-*d*) δ 9.29 (d, *J* = 9.4 Hz, 1H), 8.70 (d, *J* = 8.2 Hz, 1H), 8.34 – 8.23 (m, 3H), 8.19 (dd, *J* = 8.5, 5.3 Hz, 2H), 8.15 – 7.99 (m, 2H), 5.66 – 5.54 (m, 1H), 4.98 (t, *J* = 5.2 Hz, 1H), 4.63 (t, *J* = 5.3 Hz, 1H), 4.52 – 4.26 (m, 2H), 4.17 (dd, *J* = 9.5, 6.4 Hz, 1H), 4.04 (dd, *J* = 9.2, 6.2 Hz, 1H), 3.72 (dd, *J* = 9.2, 7.1 Hz, 1H), 2.69 (d, *J* = 8.3 Hz, 1H).

^{13}C NMR (101 MHz, Chloroform-*d*) δ 167.4, 134.8, 131.6, 131.2, 130.6, 130.0, 129.8, 128.8, 127.3, 126.6, 126.5, 126.5, 125.0, 125.0, 124.3, 122.8, 82.0, 81.0, 74.9, 74.2, 72.5, 71.4.

Elemental analysis Calcd for $C_{23}H_{18}O_5$: C, 73.79; H, 4.85; O, 21.37; Found: C, 73.68; H, 4.99.

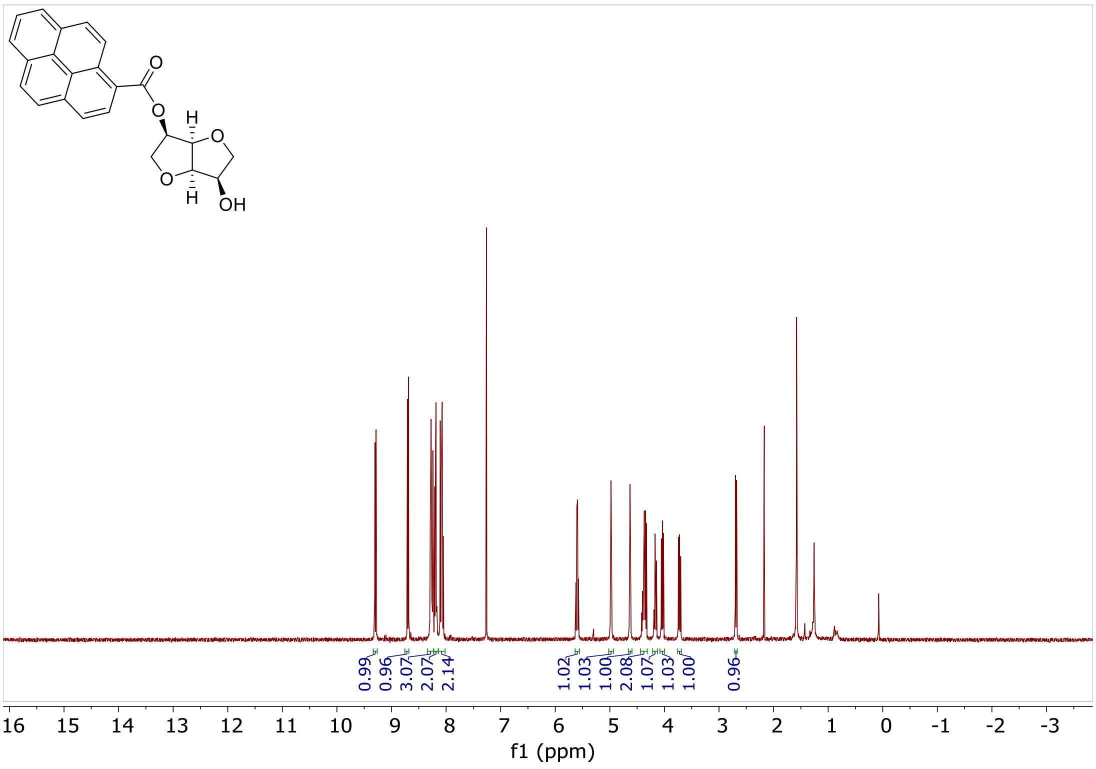

Figure S11. ^{1}H NMR (401 MHz, Chloroform-*d*) spectrum of compound **5a**.

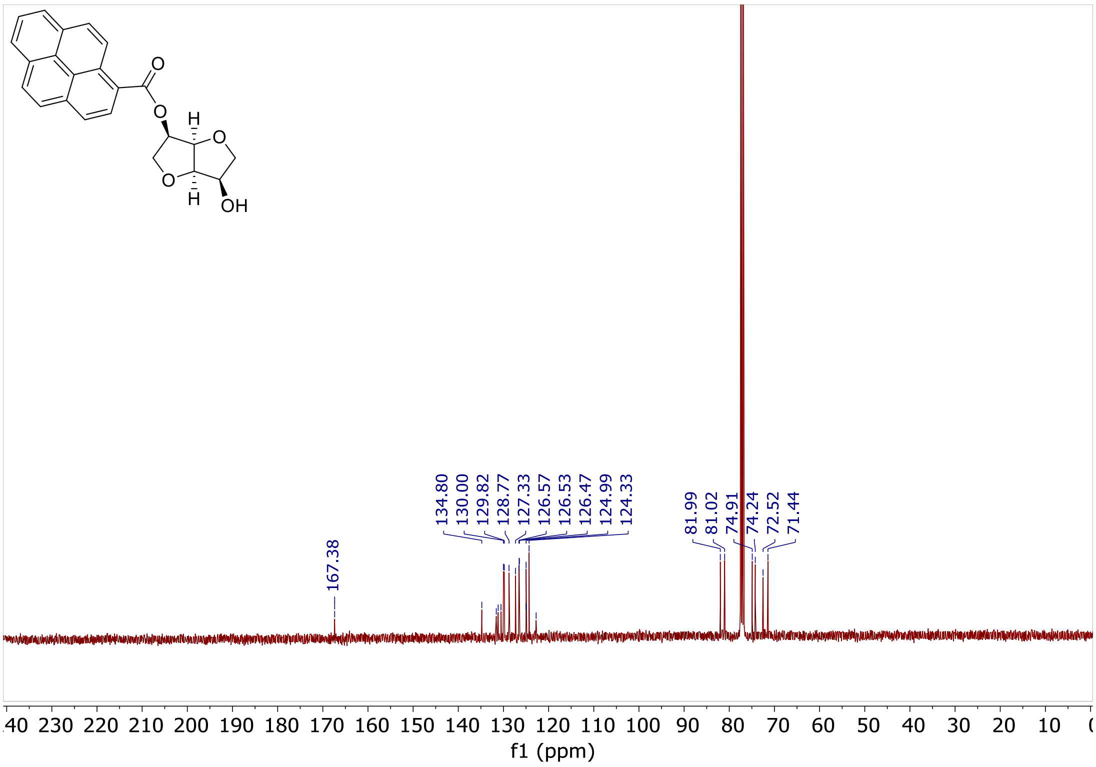

Figure S12. ^{13}C NMR (101 MHz, Chloroform-*d*) spectrum of compound **5a.**

2.8 (3R,3aR,6R,6aR)-hexahydrofuro[3,2-b]furan-3-(1-pyrenyl)acetate-6-ol, 5b

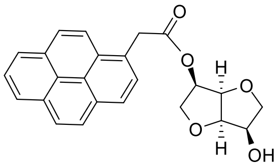

To a solution of isomannide (**1**, 242.6 mg, 1.66 mmol), EDC·HCl (123.7 mg, 0.65 mmol) and DMAP (16.7 mg, 0.14 mmol) in dry THF (20 mL), 1-pyrenylacetic acid (127.1 mg, 0.49 mmol) was added and the reaction mixture was stirred at RT for 18 hours, monitoring by TLC (CH_2Cl_2:Acetone 9:1). The solvent was removed under reduced pressure, the crude was dissolved in CH_2Cl_2 (30 mL) and the organic phase was washed with water (3x10 mL) and dried over anhydrous Na_2SO_4. The solvent was removed under the reduced pressure and the crude was purified by Biotage® Isolera chromatograph (CH_2Cl_2:Acetone) to give the pure product **5b** as a yellow solid (105.3 mg, 0.27 mmol 55%).

m.p. 147-149°C

^{1}H NMR (400 MHz, Chloroform-*d*) δ 8.27 (d, *J* = 9.3 Hz, 1H), 8.21 – 8.18 (m, 2H), 8.16 (s, 1H), 8.14 (d, *J* = 2.1 Hz, 1H), 8.06 (d, *J* = 1.3 Hz, 2H), 8.04 – 7.99 (m, 1H), 7.96 (d, *J* = 7.8 Hz, 1H), 5.18 (q, *J* = 5.8 Hz, 1H), 4.70 (t, *J* = 5.3 Hz, 1H), 4.43 (s, 2H), 4.40 (t, *J* = 5.3 Hz, 1H), 4.19 (ddd, *J* = 7.4, 6.3, 5.5 Hz, 1H), 4.02 (dd, *J* = 9.7, 6.1 Hz, 1H), 3.84 (dd, *J* = 9.7, 5.7 Hz, 1H), 3.75 (dd, *J* = 9.2, 6.4 Hz, 1H), 3.30 (dd, *J* = 9.2, 7.4 Hz, 1H), 2.08 (bs, 1H).

^{13}C NMR (101 MHz, Chloroform-*d*) δ 171.0, 131.4, 131.0, 130.9, 129.6, 128.6, 127.7, 127.5, 126.1, 125.4, 125.3, 125.1, 125.0, 124.8, 123.4, 81.6, 80.5, 74.7, 73.6, 72.2, 71.2, 39.2.

Elemental analysis Calcd for $C_{24}H_{20}O_5$: C, 74.21; H, 5.19; O, 20.60; Found: C, 74.11; H, 5.36.

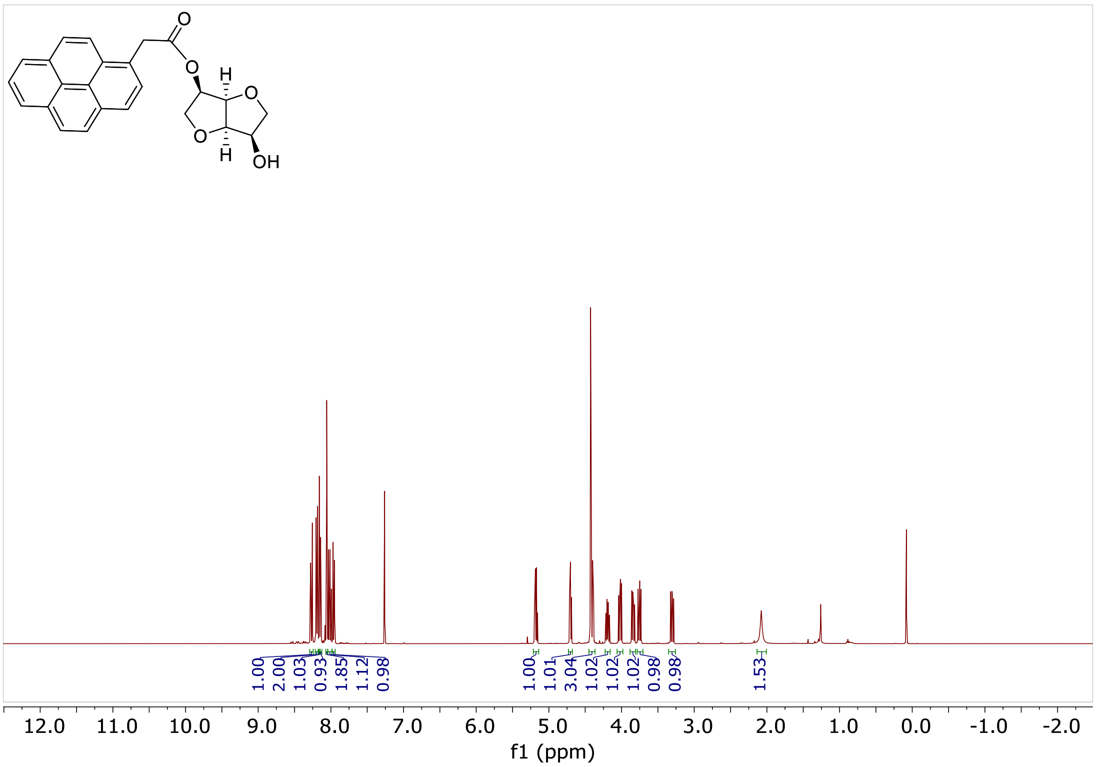

Figure S13. ¹H NMR (401 MHz, Chloroform-*d*) spectrum of compound **5b.**

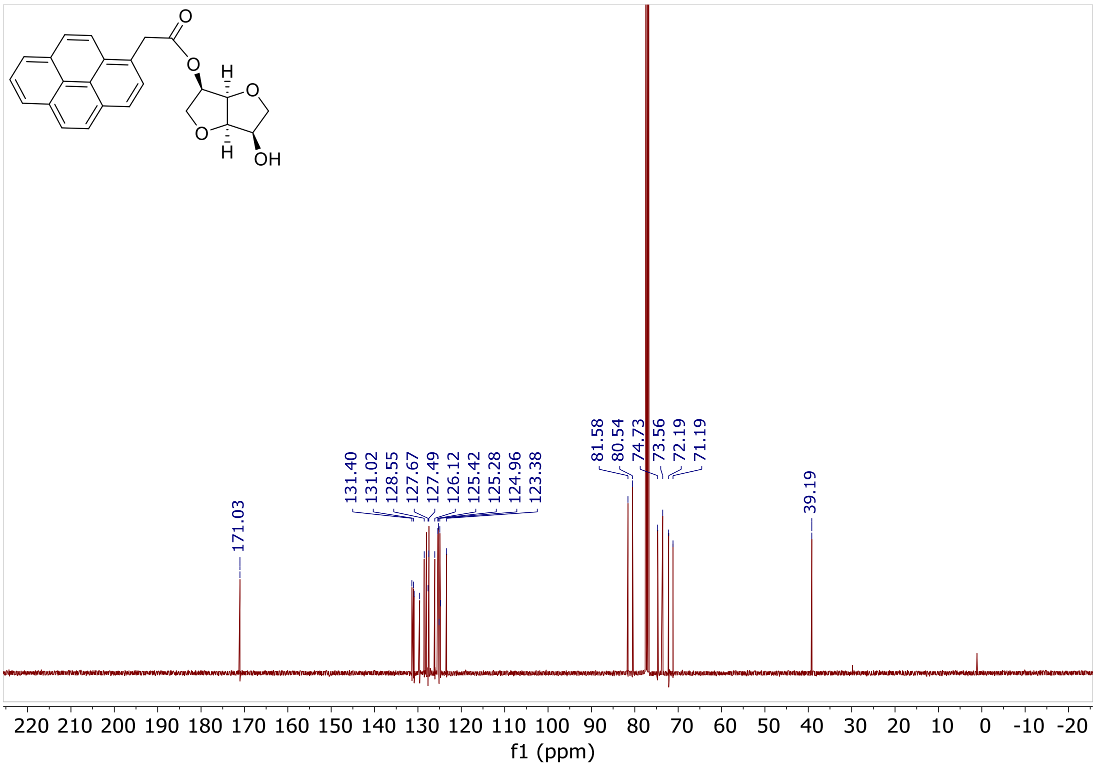

Figure S14. ¹³C NMR (101 MHz, Chloroform-*d*) spectrum of compound **5b.**

2.9 (3R,3aR,6R,6aR)-hexahydrofuro[3,2-b]furan-3-(3-perylenyl)carboxylate-6-ol, 5c

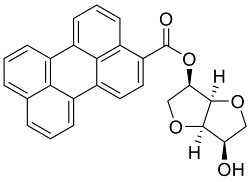

To a solution of isomannide (**1**, 135.8 mg, 0.93 mmol), EDC·HCl (76.7 mg, 0.40 mmol) and DMAP (9.0 mg, 0.07 mmol) in dry THF (12 mL), 3-perylencarboxylic acid (**7**, 88.8 mg, 0.30 mmol) was added and the reaction mixture was stirred at RT for 21 hours, monitoring by TLC (CH_2Cl_2:Acetone 9:1). The solvent was removed under reduced pressure, the crude was dissolved in CH_2Cl_2 (20 mL) and the organic phase was washed with water (3x10 mL) and dried over anhydrous Na_2SO_4. The solvent was removed under the reduced pressure and the crude was purified by Biotage® Isolera chromatograph (CH_2Cl_2:Acetone) to give the pure product **5** as a yellow solid (34.8 mg, 0.08 mmol, 27%).

m.p. 212-215°C

^{1}H NMR (400 MHz, Chloroform-*d*) δ 8.87 (dd, *J* = 8.6, 0.9 Hz, 1H), 8.31 – 8.22 (m, 4H), 8.19 (d, *J* = 8.1 Hz, 1H), 7.80 – 7.75 (m, 1H), 7.75 – 7.69 (m, 1H), 7.62 (dd, *J* = 8.6, 7.5 Hz, 1H), 7.52 (ddd, *J* = 8.1, 7.5, 2.3 Hz, 2H), 5.54 – 5.45 (m, 1H), 4.92 (t, *J* = 5.2 Hz, 1H), 4.60 (t, *J* = 5.3 Hz, 1H), 4.37 (ddd, *J* = 6.9, 6.2, 5.6 Hz, 1H), 4.30 (dd, *J* = 9.6, 6.3 Hz, 1H), 4.10 (dd, *J* = 9.5, 6.4 Hz, 1H), 4.03 (dd, *J* = 9.2, 6.2 Hz, 1H), 3.70 (dd, *J* = 9.2, 7.0 Hz, 1H).

^{13}C NMR (101 MHz, Chloroform-*d*) δ 166.7, 136.4, 134.6, 133.3, 131.4, 131.1, 130.3, 129.5, 129.3, 128.4, 128.4, 128.3, 127.0, 126.8, 125.7, 125.1, 122.2, 121.3, 120.8, 119.1, 81.9, 81.0, 74.7, 74.2, 72.5, 71.4.

Elemental analysis Calcd for $C_{28}H_{24}O_5$: C, 76.35; H, 5.49; O, 18.16; Found: C, 76.27; H, 5.71.

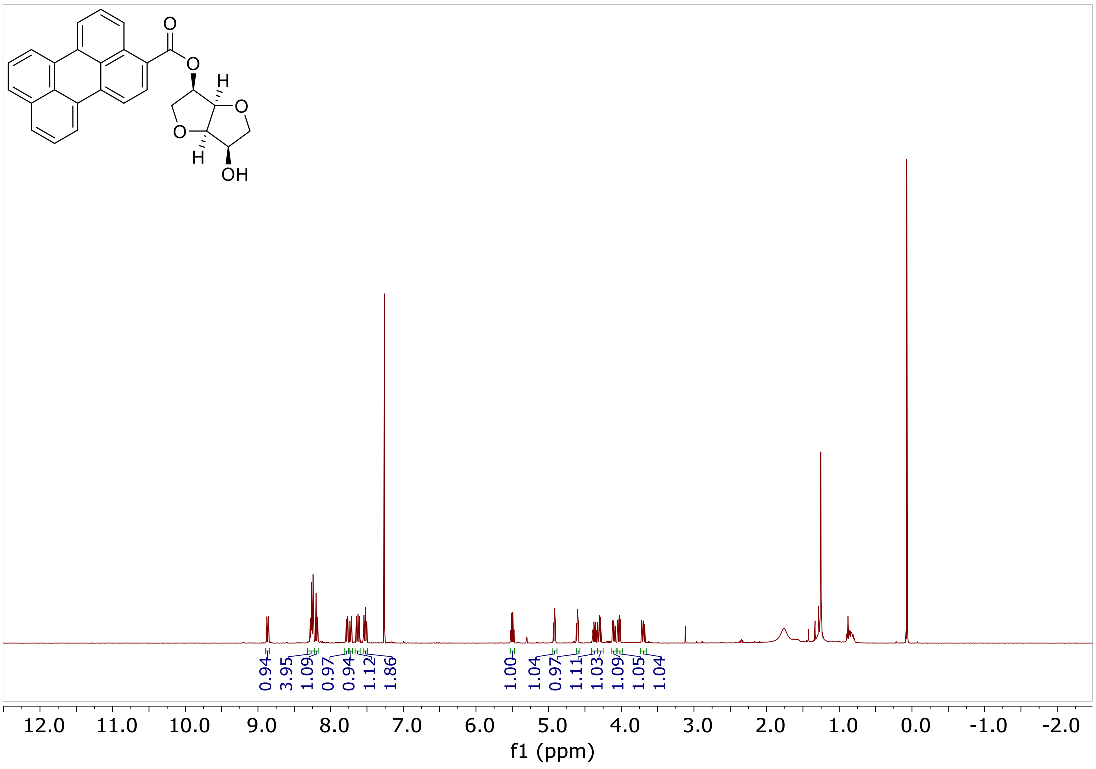

Figure S15. ^{1}H NMR (401 MHz, Chloroform-*d*) spectrum of compound **5c.**

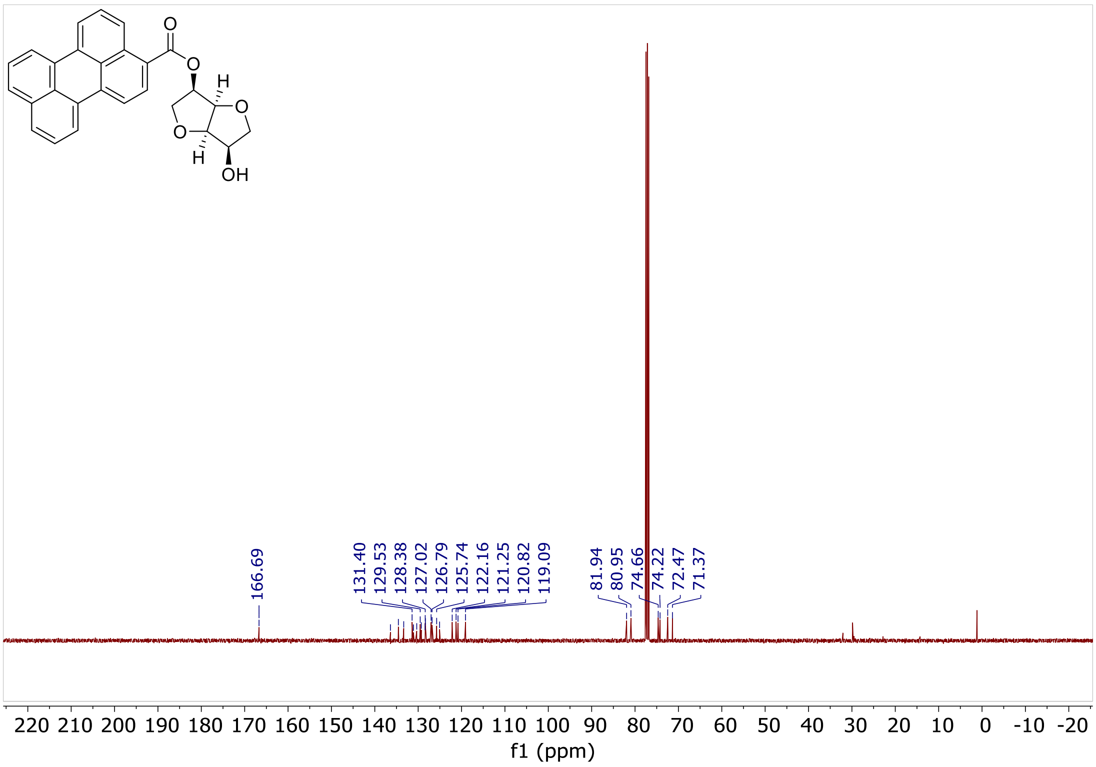

Figure S16. ^{13}C NMR (101 MHz, Chloroform-*d*) spectrum of compound **5c.**

2.10 (3R,3aR,6R,6aR)-hexahydrofuro[3,2-b]furan-3-(1-pyrenyl)carboxylate-6-(1-pyrenyl)acetate, 3d

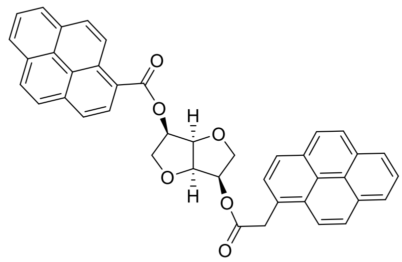

Compound **5** (41.7 mg, 0.11 mmol), EDC·HCl (29.5 mg, 0.15 mmol), DMAP (18.2 mg, 0.15 mmol), were dissolved in dry CH2Cl2 (2 mL) and 1-pyrenylacetic acid (36.4 mg, 0.14 mmol) was added. The reaction mixture was stirred at RT, monitoring by TLC (CH_2Cl_2:Acetone 9:1). After 24 h, the reaction mixture was diluted with CH_2Cl_2 (20mL), washed with H_2O (3x10 mL) and dried over anhydrous Na_2SO_4. The solvent was removed under reduced pressure and the crude was purified by recrystallization from CH_2Cl_2/MeOH, to give the pure product as a light yellow solid (50.8 mg, 0.08 mmol, 75%).

m.p. 156-158°C

^{1}H NMR (401 MHz, Chloroform-*d*) δ 9.25 (d, *J* = 9.4 Hz, 1H), 8.60 (d, *J* = 8.1 Hz, 1H), 8.34 – 7.90 (m, 16H), 5.43 (q, *J* = 6.4 Hz, 1H), 5.21 (q, *J* = 6.1 Hz, 1H), 4.88 (dt, *J* = 24.3, 5.3 Hz, 2H), 4.46 (s, 2H), 4.12 – 4.00 (m, 2H), 3.97 – 3.83 (m, 2H).

^{13}C NMR (101 MHz, Chloroform-*d*) δ 171.2, 167.3, 134.7, 131.5, 131.4, 131.1, 131.1, 130.9, 130.5, 129.9, 129.8, 129.7, 128.7, 128.6, 128.1, 127.8, 127.5, 127.5, 127.3, 126.5, 126.5, 126.4, 126.1, 125.4, 125.3, 125.2, 125.0, 125.0, 124.8, 124.3, 124.3, 123.5, 122.7, 80.8, 80.7, 74.5, 74.3, 71.0, 70.7, 39.2.

Elemental analysis Calcd for $C_{41}H_{28}O_8$: C, 79.86; H, 4.58; O, 15.57; Found: C, 79.73; H, 4.65.

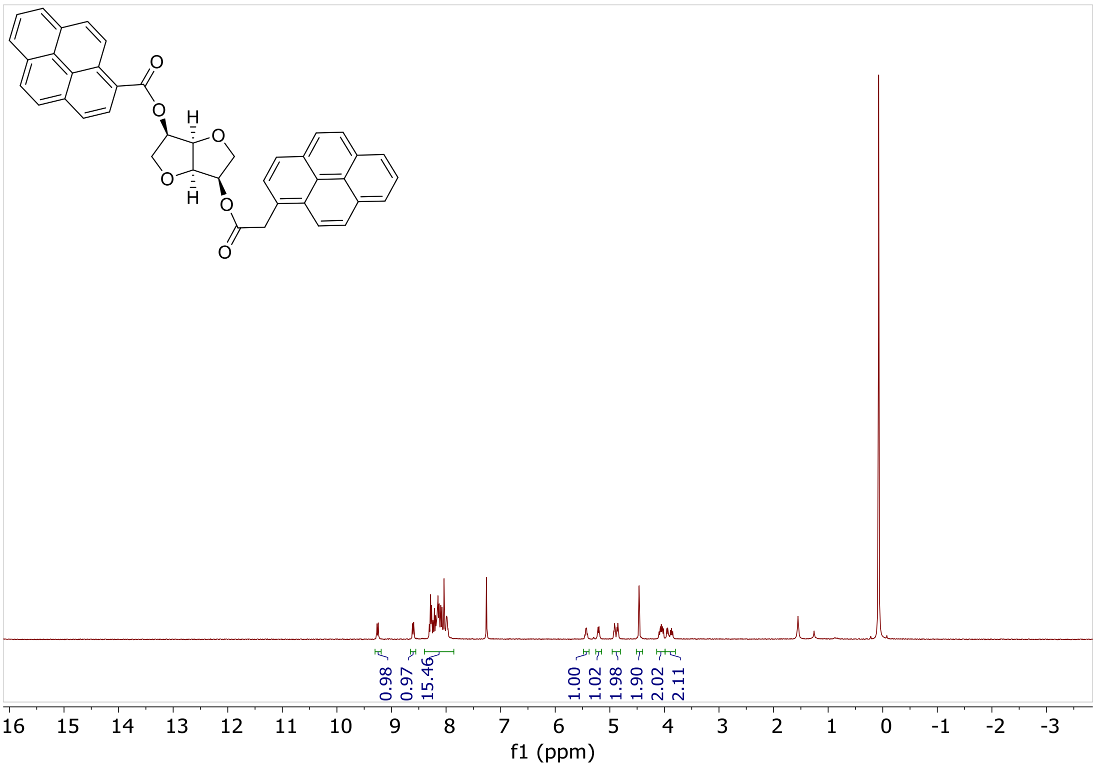

Figure S17. ^{1}H NMR (401 MHz, Chloroform-*d*) spectrum of compound **3d.**

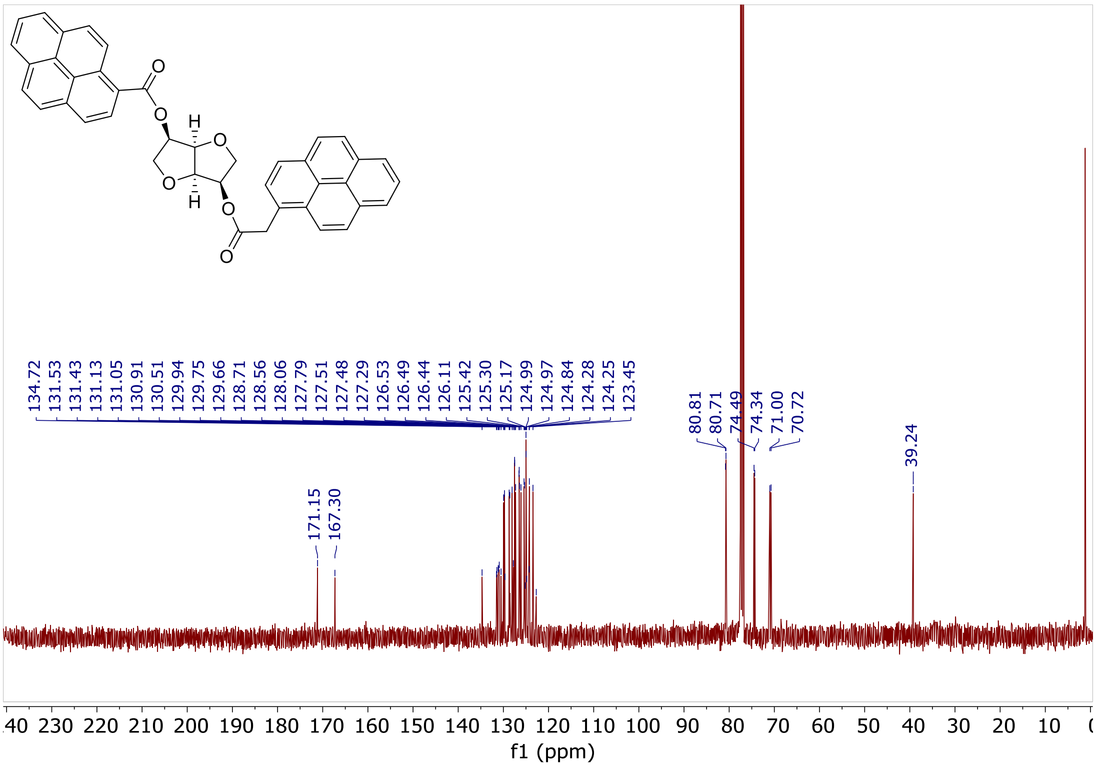

Figure S18. ^{13}C NMR (101 MHz, Chloroform-*d*) spectrum of compound **3d.**

2.11 (3R,3aR,6R,6aR)-hexahydrofuro[3,2-b]furan-3-(1-pyrenyl)carboxylate-6-(3-perylenyl)carboxylate, 3e

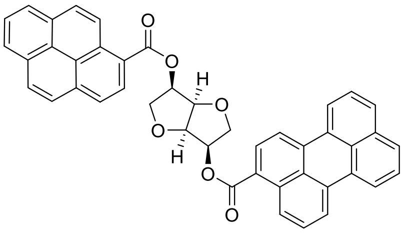

Compound **5** (58.6 mg, 0.16 mmol), EDC·HCl (50.6 mg, 0.26 mmol), DMAP (30.7 mg, 0.25 mmol), were dissolved in dry CH_2Cl_2 (3 mL) and 3-perylencarboxylic acid (78.4 mg, 0.26 mmol) was added. The reaction mixture was stirred for 24h, monitoring by TLC (CH_2Cl_2:Acetone 9:1). After 24 h, the reaction mixture was diluted with CH_2Cl_2 (20mL), washed with H_2O (3x10 mL) and dried over anhydrous Na_2SO_4. The solvent was removed under reduced pressure and the crude was purified by Biotage® Isolera chromatograph (CH_2Cl_2:Acetone) to give the pure product as an orange glassy solid (40.3 mg, 0.06 mmol, 39%).

m.p. 201-207°C

^{1}H NMR (401 MHz, Chloroform-*d*) δ 9.31 (d, *J* = 9.5 Hz, 1H), 8.89 (d, *J* = 8.6 Hz, 1H), 8.71 (d, *J* = 8.1 Hz, 1H), 8.32 – 8.19 (m, 6H), 8.20 – 8.11 (m, 3H), 8.11 – 8.01 (m, 3H), 7.73 (dd, *J* = 17.2, 8.1 Hz, 2H), 7.66 – 7.56 (m, 1H), 7.55 – 7.45 (m, 2H), 5.60 (q, *J* = 6.1 Hz, 1H), 5.52 (q, *J* = 6.1 Hz, 1H), 5.16 – 5.02 (m, 2H), 4.43 – 4.14 (m, 4H).

^{13}C NMR (101 MHz, Chloroform-*d*) δ 167.5, 166.7, 136.2, 134.7, 134.5, 133.2, 131.5, 131.3, 131.1, 131.0, 130.5, 130.2, 129.9, 129.8, 129.4, 129.1, 128.8, 128.3, 128.3, 128.2, 127.3, 126.9, 126.7, 126.5, 126.5, 126.4, 125.7, 125.1, 125.0, 124.9, 124.3, 122.9, 122.0, 121.1, 120.7, 118.9, 81.2, 81.2, 74.6, 74.4, 71.5, 71.4.

Elemental analysis Calcd for $C_{44}H_{28}O_8$: C, 80.97; H, 4.32; O, 14.71; Found C, 80.82; H, 4.55.

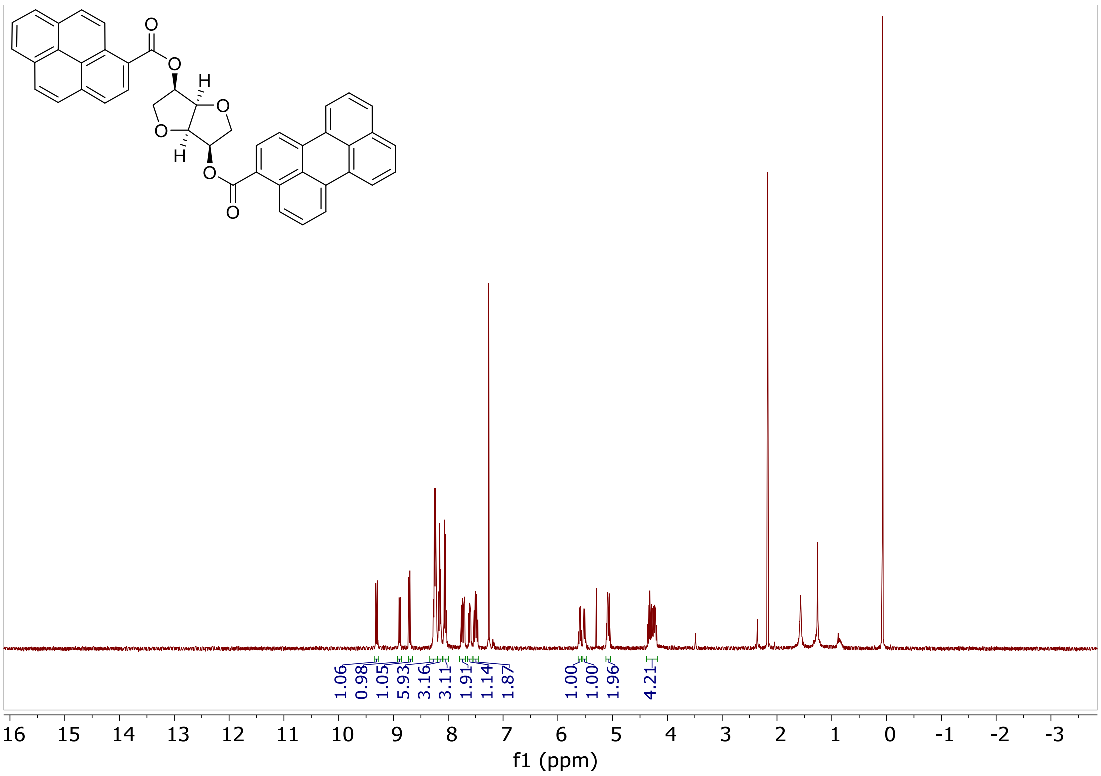

Figure S19. ^{1}H NMR (401 MHz, Chloroform-*d*) spectrum of compound **3e.**

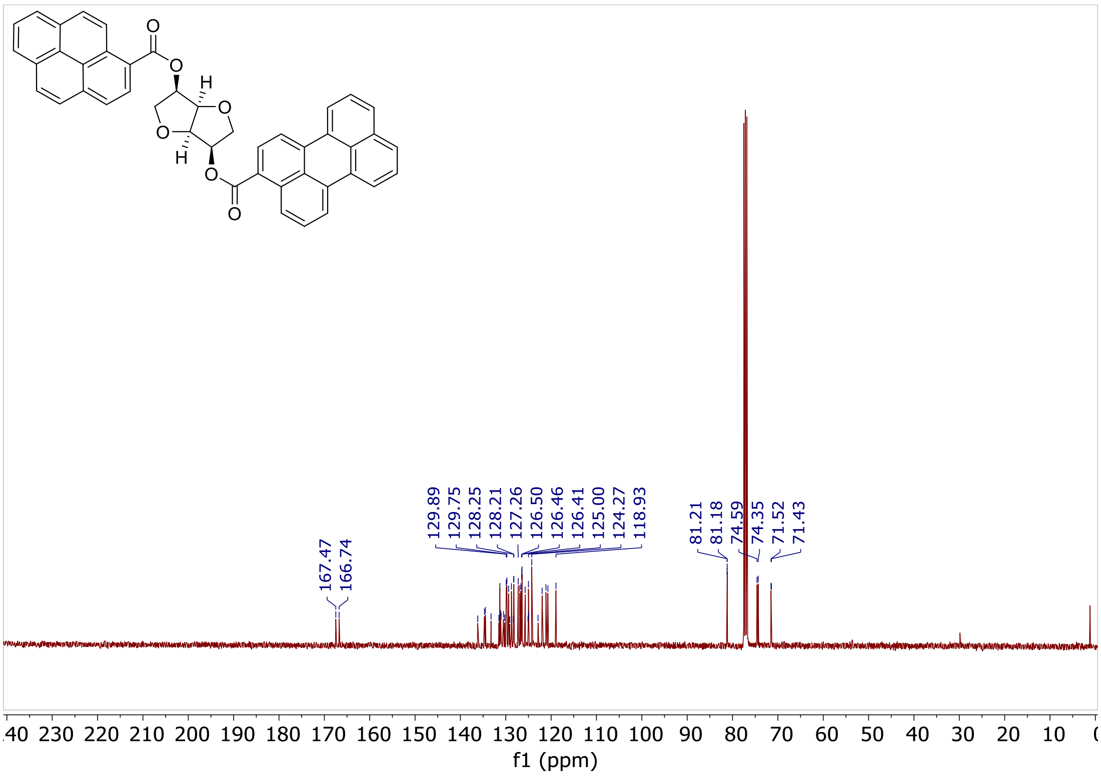

Figure S20. ^{13}C NMR (101 MHz, Chloroform-*d*) spectrum of compound **3e.**

2.12 3-acetylperylene, 6[2]

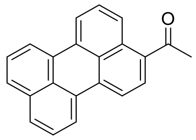

Under an Ar atmosphere, to an orange solution of perylene (1.27 g, 5.05 mmol) in dry chlorobenzene (140 mL) at 40°C, dry acetyl chloride (650 µL, 9.11 mmol) was added. Then, a solution of $AlCl_3$ (1.2070 g, 9.05 mmol) in dry nitromethane (9.5 mL) was slowly added in 35 minutes. The black mixture was heathed at 40°C for additional 15 minutes and then stirred at room-temperature. The reaction was monitored by TLC analysis (Toluene). After 5 h additional acetyl chloride (0.70 mL, 9.81 mmol) and $AlCl_3$ (1.0550 g, 7.91 mmol) were added. The mixture was stirred for additional 15 h at room-temperature and then quenched by addition of a mixture containing 36 g of ice, 80 mL of water and 10 mL of 37% HCl). The pure product, crystallized as an orange solid, was filtered (693.2 mg, 2.35 mmol, 47%). The filtrate was washed with H_2O (2x50 mL). The washes were reunited and extracted with toluene (50 mL), the organic phases were reunited and dried over anhydrous Na_2SO_4. The solvent was removed under reduced pressure to give the pure product as an orange solid (719.3 mg, 2.44 mmol, 48%). The total yield was 95%.

m.p. 238-241°C (*lit.* [2] *245-247°C)*

^{1}H NMR (401 MHz, Chloroform-*d*) δ 8.70 (d, *J* = 8.6 Hz, 1H), 8.30 – 8.21 (m, 3H), 8.17 (d, *J* = 7.9 Hz, 1H), 7.94 (d, *J* = 7.9 Hz, 1H), 7.74 (dd, *J* = 19.3, 8.1 Hz, 2H), 7.61 (t, *J* = 8.0 Hz, 1H), 7.52 (t, *J* = 7.8 Hz, 2H), 2.75 (s, 3H).

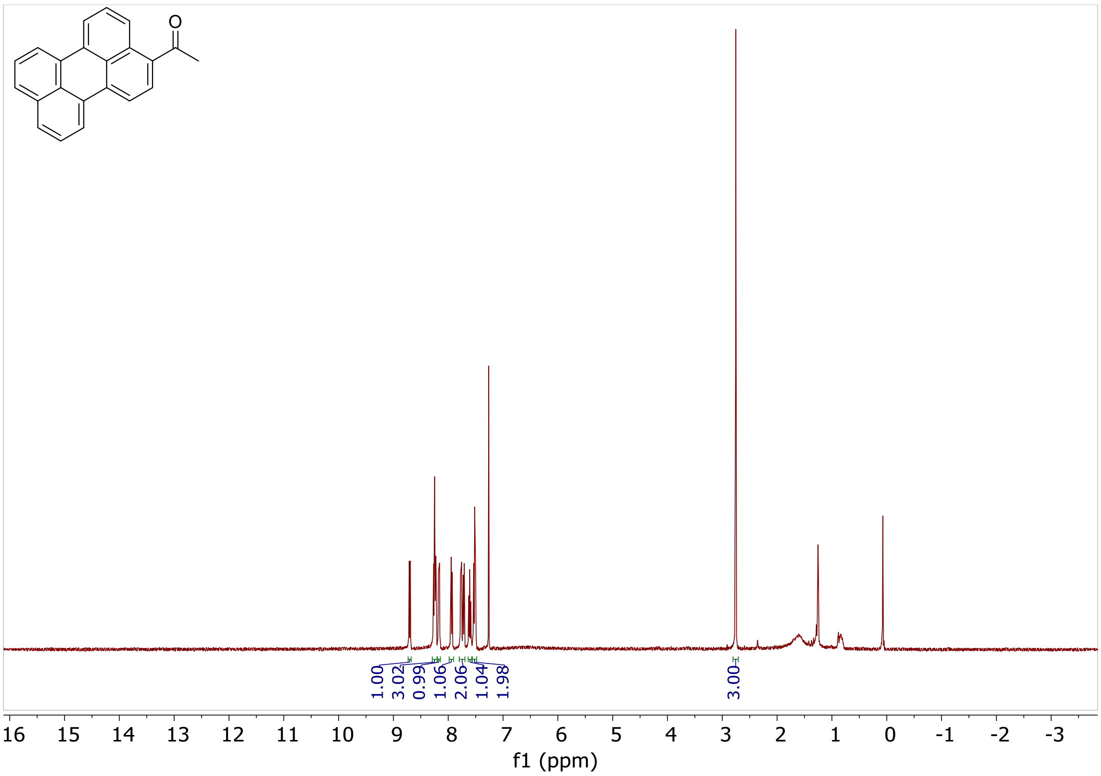

Figure S21. ^{1}H NMR (401 MHz, DMSO-d_6) spectrum of compound **6** .

2.13 3-perylencarboxylic acid, 7[3]

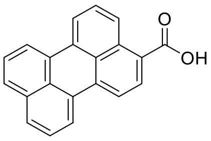

To a pre-cooled solution of NaOH (3.8409 g, 96.0 mmol) in water (71 mL), 1,4-dioxane (71 mL) and Br_2 (1.5 mL, 29.3 mmol) were added. The mixture was stirred for 10 minutes and then **6** (1.4125 g, 4.79 mmol) was slowly added in portions over 20 minutes. The mixture was stirred for additional 3 h at 0°C and then at room temperature, monitoring the reaction by TLC analysis (Toluene). After 15 h further Br_2 (1.5 mL, 29.3 mmol) and NaOH (3.4829 g, 87.07 mmol) were added. After 5 h of additional stirring, the yellow solid was filtered off and washed with water (20 mL), 1,4-dioxane (20 mL), 1M $Na_2S_2O_{3(aq)}$ (10 mL), water (10 mL) and chloroform (20 mL) and dried under vacuum. The initial filtrate was treated with 1M $Na_2S_2O_{3\,(aq)}$ (20 mL) and acidified to pH 3 with a 1M aqueous solution of citric acid. The precipitated solid was filtered and dried under vacuum to yield 247.5 mg (0.84 mmol, 17%) of pure product as an orange solid. The initial golden solid was disperded in water (10 mL) and acified to pH 3 with a 1M aqueous solution of citric acid. The mixture was stirred for 2 h, the solid was filtered and dried under vacuum to yield the pure product as an orange solid (740.1 mg, 2.50 mmol, 52%). The total yield was 69%.

m.p. > 325°C (*lit.* [3] *331-334°C)*

^{1}H NMR (401 MHz, DMSO-d_6) δ 8.80 (d, J = 8.6 Hz, 1H), 8.47 – 8.35 (m, 4H), 8.08 (d, J = 7.9 Hz, 1H), 7.85 (dd, J = 14.5, 8.1 Hz, 2H), 7.68 – 7.50 (m, 3H).

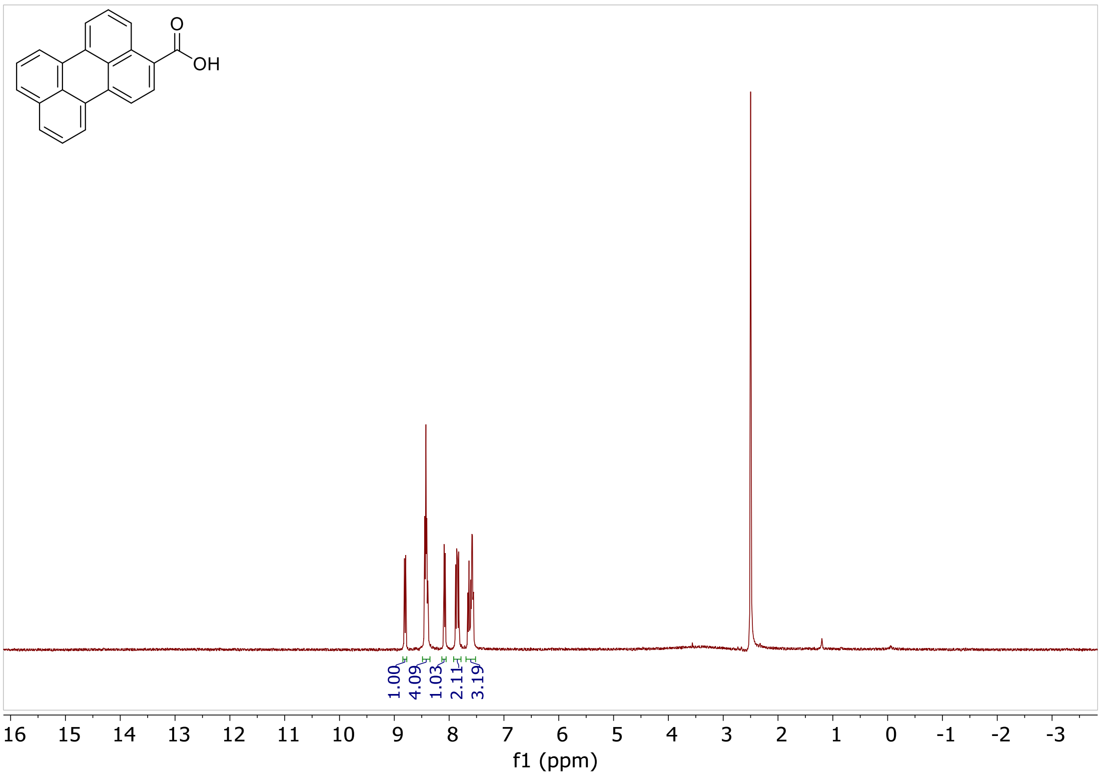

Figure S22. ^{1}H NMR (401 MHz, DMSO-d_6) spectrum of compound **7**.

3 UV-Vis and ECD spectra of compounds 3, 4 and 5.

3.1 (3R,3aR,6R,6aR)-hexahydrofuro[3,2-b]furan-3,6-di-(3-perylenyl)carboxylate, 3c

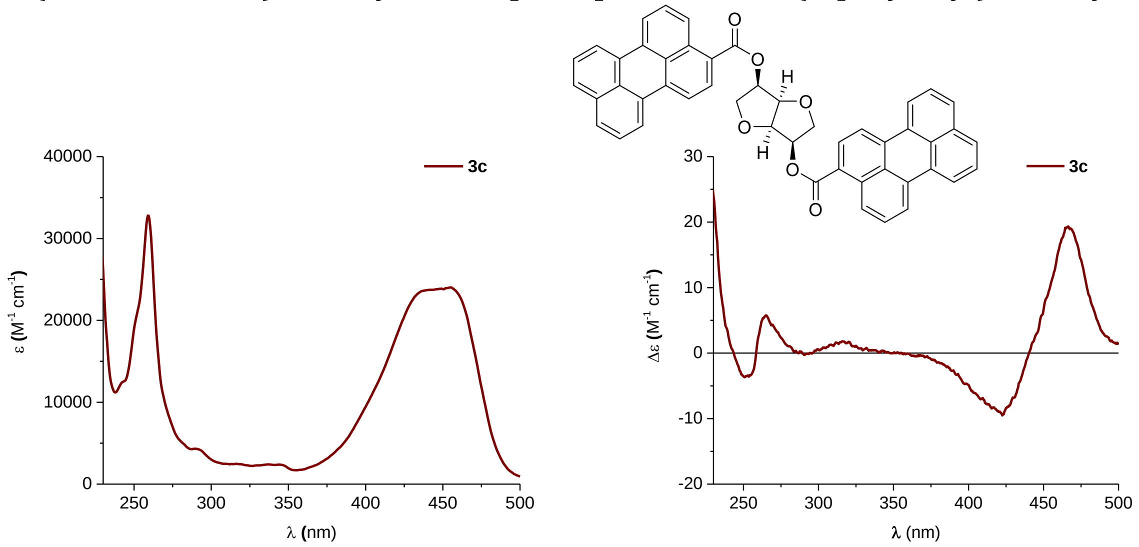

Figure S23. UV-Vis (left side) and ECD (right side) spectra of compound **3c** (c=1.6·10^{-5} M in CH_2Cl_2, 1 cm path length).

3.2 (3R,3aR,6R,6aR)-hexahydrofuro[3,2-b]furan-3-(1-pyrenyl)carboxylate-6-(1-pyrenyl)acetate, 3d

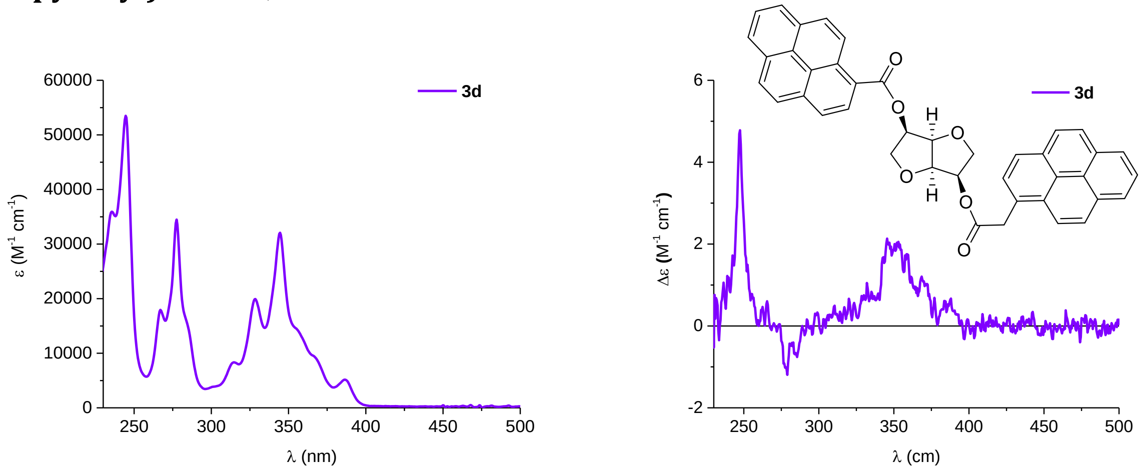

Figure S24. UV-Vis (left side) and ECD (right side) spectra of compound **3d** (c=1.5·10^{-5} M in CH_2Cl_2, 1 cm path length).

3.3 (3R,3aR,6R,6aR)-hexahydrofuro[3,2-b]furan-3-(1-pyrenyl)carboxylate-6-(3-perylenyl)carboxylate, 3e

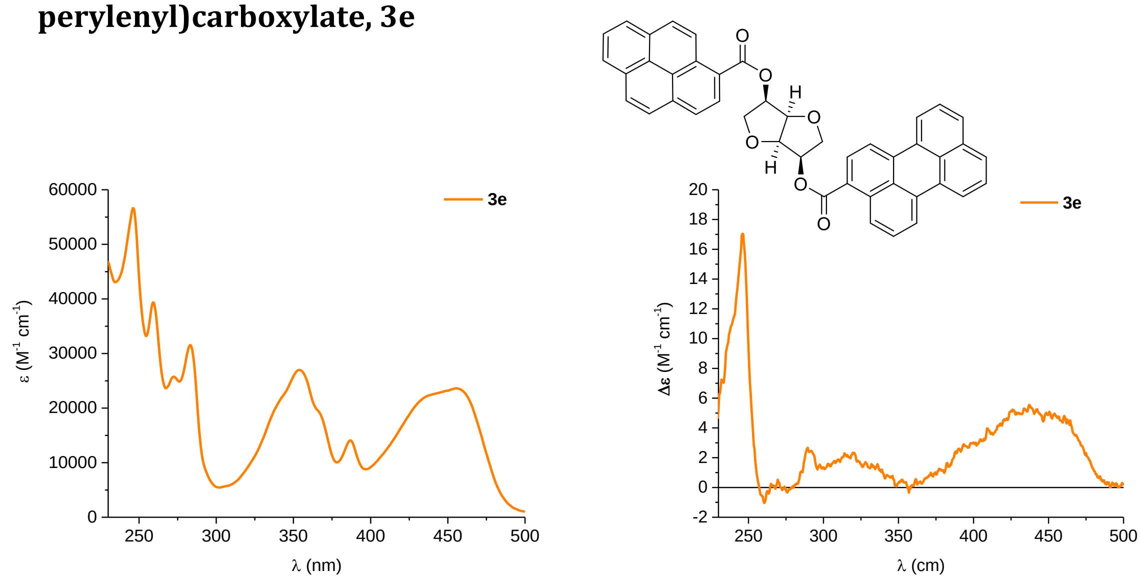

Figure S25. UV-Vis (left side) and ECD (right side) spectra of compound **3e** (c=1.8·10^{-5} M in CH_2Cl_2, 1 cm path length).

3.4 (3R,3aR,6R,6aR)-hexahydrofuro[3,2-b]furan-3-(1-pyrenyl)carboxylate-6-ol, 5a

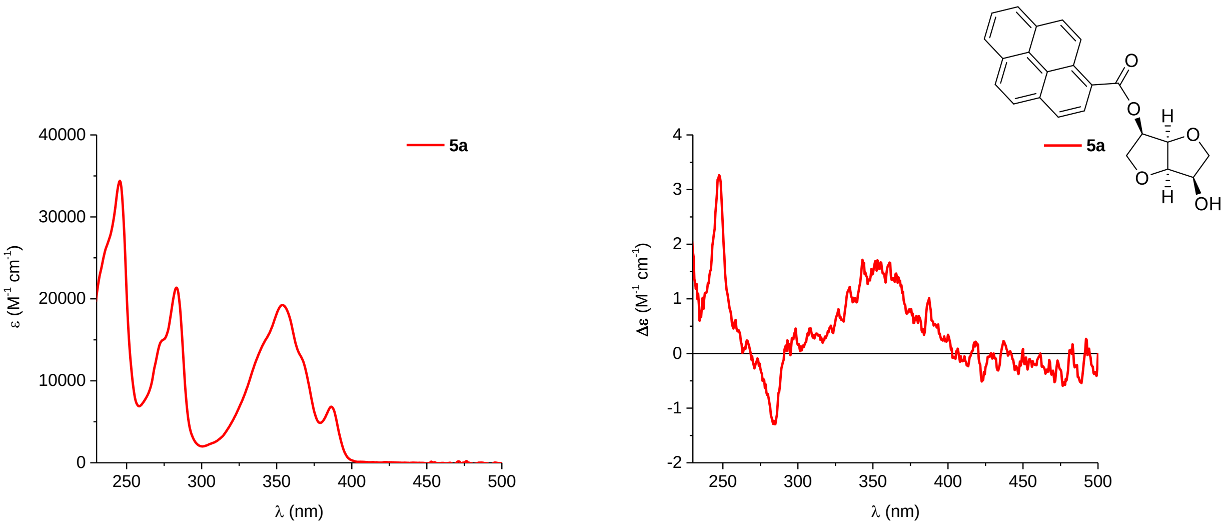

Figure S26. Fluorescence spectra (left side) and circularly polarized luminescence spectra (right side) of compound **5a** (c=1.8·10^{-5} M in CH_2Cl_2, 1 cm path length).

3.5 (3R,3aR,6R,6aR)-hexahydrofuro[3,2-b]furan-3-(1-pyrenyl)acetate-6-ol, 5b

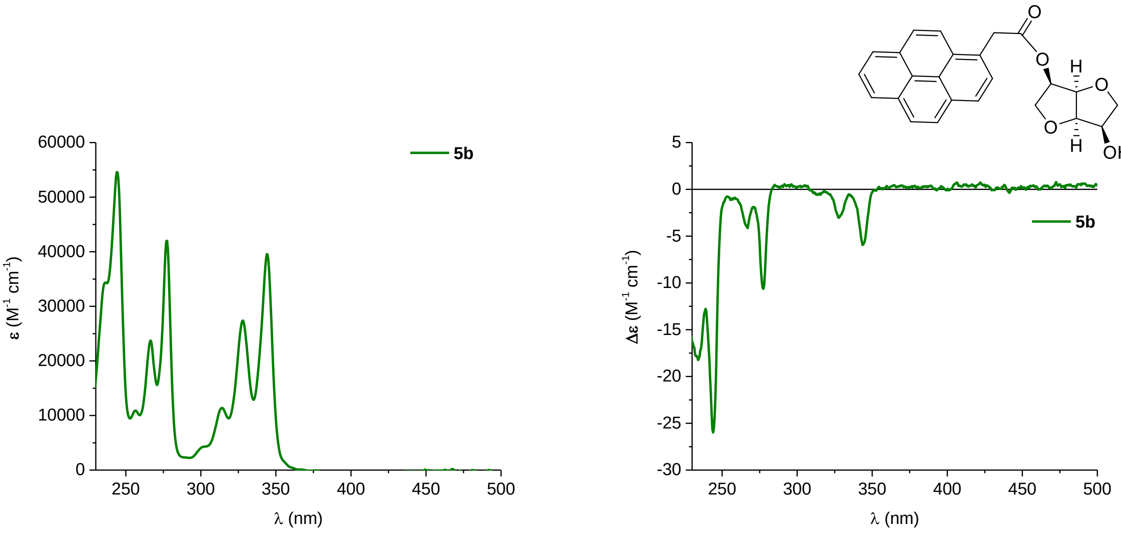

Figure S27. Fluorescence spectra (left side) and circularly polarized luminescence spectra (right side) of compound **5b** (c=1.7·10^{-5} M in CH_2Cl_2, 1 cm path length).

3.6 (3R,3aR,6R,6aR)-hexahydrofuro[3,2-b]furan-3-(3-perylenyl)carboxylate-6-ol, 5c

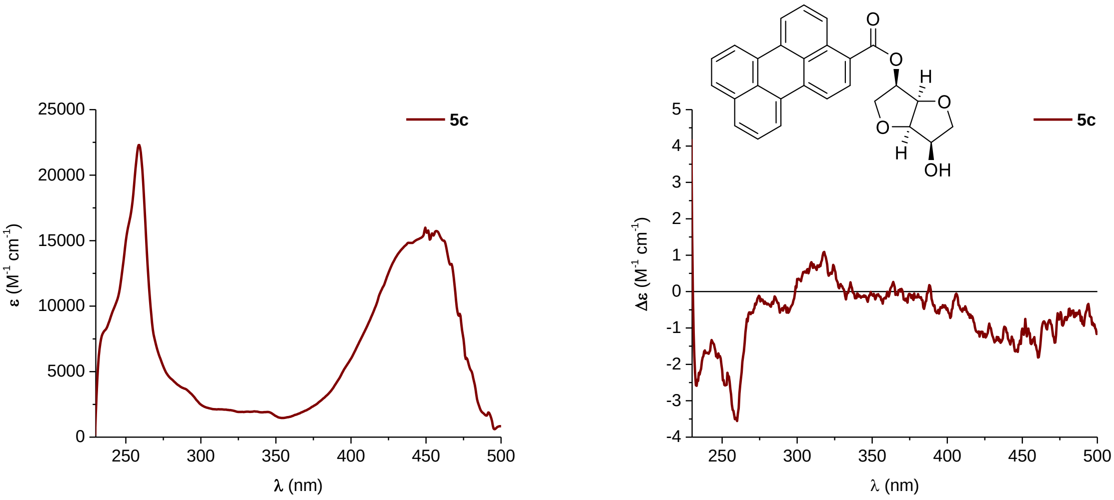

Figure S28. Fluorescence spectra (left side) and circularly polarized luminescence spectra (right side) of compound **5c** (c=1.9·10^{-5} M in CH_2Cl_2, 1 cm path length).

4 Circularly polarized luminescence (CPL) spectra of compounds 3e and 5.

4.1 (3R,3aR,6R,6aR)-hexahydrofuro[3,2-b]furan-3-(1-pyrenyl)carboxylate-6-(3-perylenyl)carboxylate, 3e

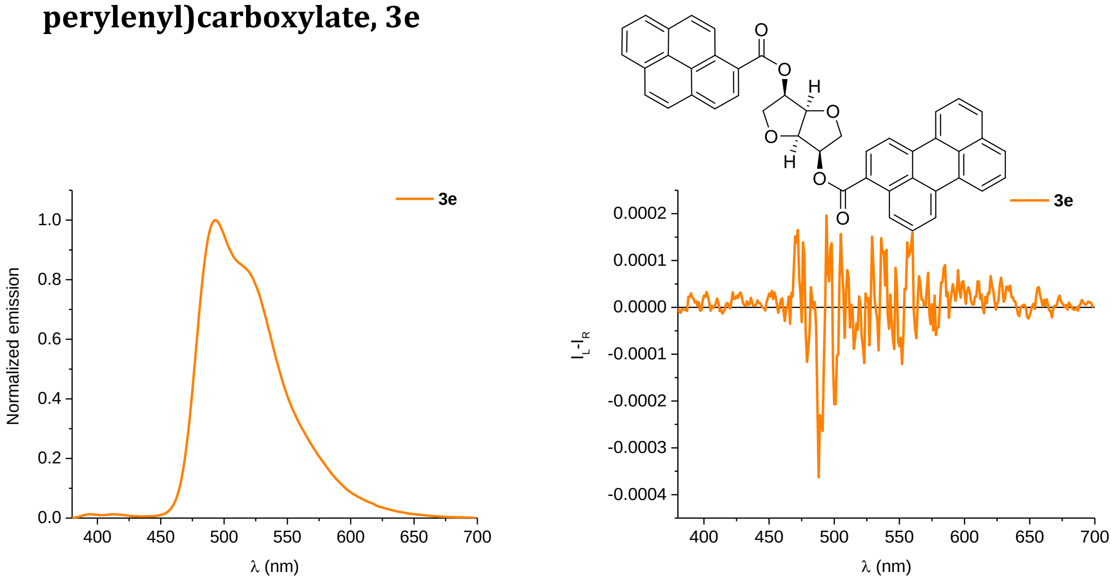

Figure S29. Fluorescence spectra (left side) and circularly polarized luminescence spectra (right side) of compound **3e** (c=1.8·10^{-5} M in CH_2Cl_2, 1 cm path length).

4.2 (3R,3aR,6R,6aR)-hexahydrofuro[3,2-b]furan-3-(1-pyrenyl)carboxylate-6-ol, 5a

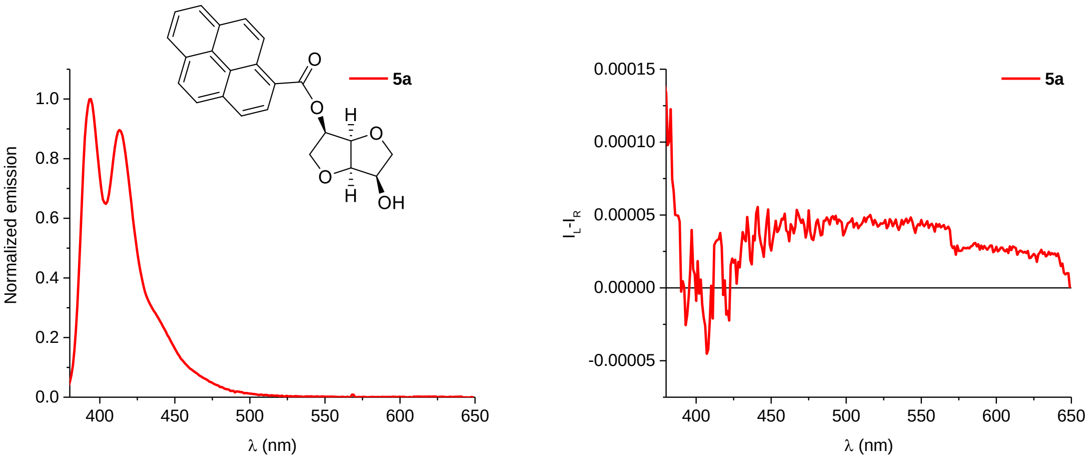

Figure S30. Fluorescence spectra (left side) and circularly polarized luminescence spectra (right side) of compound **5a** (c=1.8·10^{-5} M in CH_2Cl_2, 1 cm path length).

4.3 (3R,3aR,6R,6aR)-hexahydrofuro[3,2-b]furan-3-(1-pyrenyl)acetate-6-ol, 5b

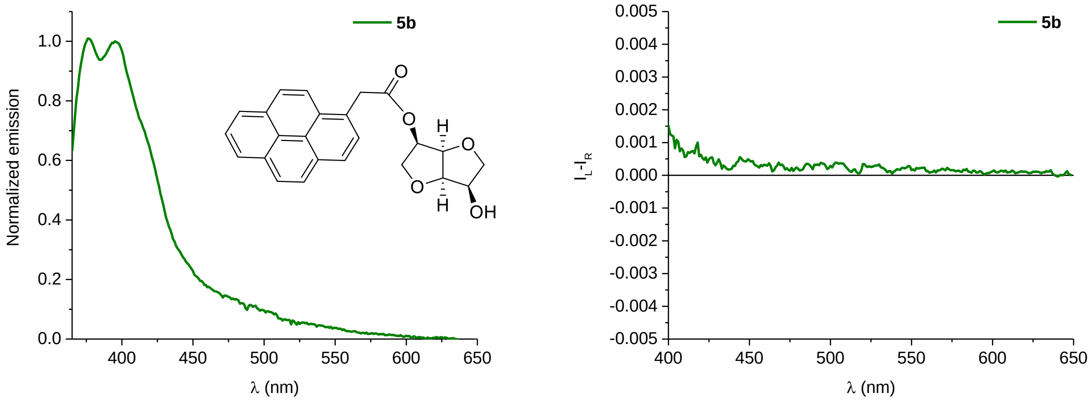

Figure S31. Fluorescence spectra (left side) and circularly polarized luminescence spectra (right side) of compound **5b** (c=1.7·10^{-5} M in CH_2Cl_2, 1 cm path length).

4.4 (3R,3aR,6R,6aR)-hexahydrofuro[3,2-b]furan-3-(3-perylenyl)carboxylate-6-ol, 5c

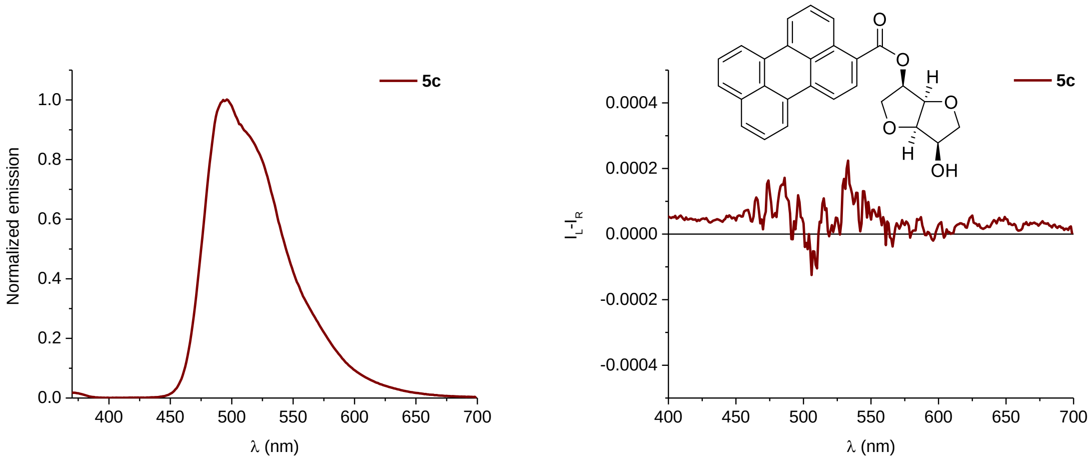

Figure S32. Fluorescence spectra (left side) and circularly polarized luminescence spectra (right side) of compound **5c** (c=1.9·10^{-5} M in CH_2Cl_2, 1 cm path length).

4.5 Superimposition of fluorescence spectra

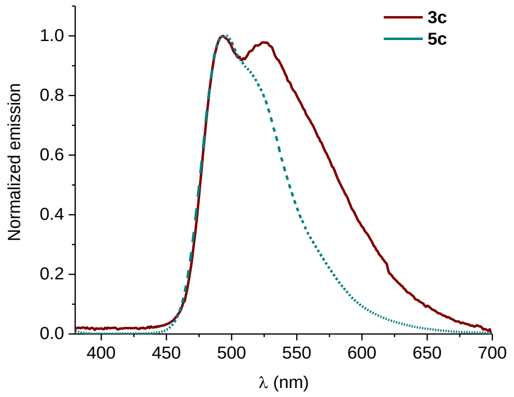

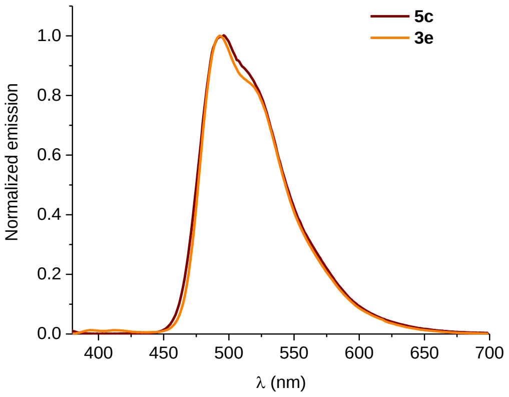

Figure S33. Left side: superimposition of fluorescence spectra of compounds **3c** (brown line, c=1.6·10^{-5} M) and **5c** (green line, c=1.9·10^{-5} M). Right side: superimposition of fluorescence spectra of compounds **5c** (dark red line, c=1.9·10^{-5} M) and **3e** (orange line, c=1.8·10^{-5} M). (CH_2Cl_2, 1 cm path length).

4.6 ^{1}H NMR comparison between monoester 5 and diesters 3

The aromatic siginals of pyrene display no significant chemical shift change moving from reference monoester **5a** to diesters **3a**, **3d** and **3e** (see the expansion of the NMR spectra in **Figure S37**), indicating a lack of strong π-π interaction between the aromatic moieties in the ground state. The same trend could be observed for 1-pyrenylacetate unit aromatic signals in derivatives **5b**, **3b**, **3d** (see **Figure S38**) and for perylene unit aromatic signals in derivative **3e** (see **Figure S39**). For derivative **3d**, possessing two perylene units, a change in the shape of ^{1}H NMR spectra could be observed with respect to reference monoester **5c** (see **Figure S40**). This evidence suggests a π-π interaction between the aromatic units in the ground state.

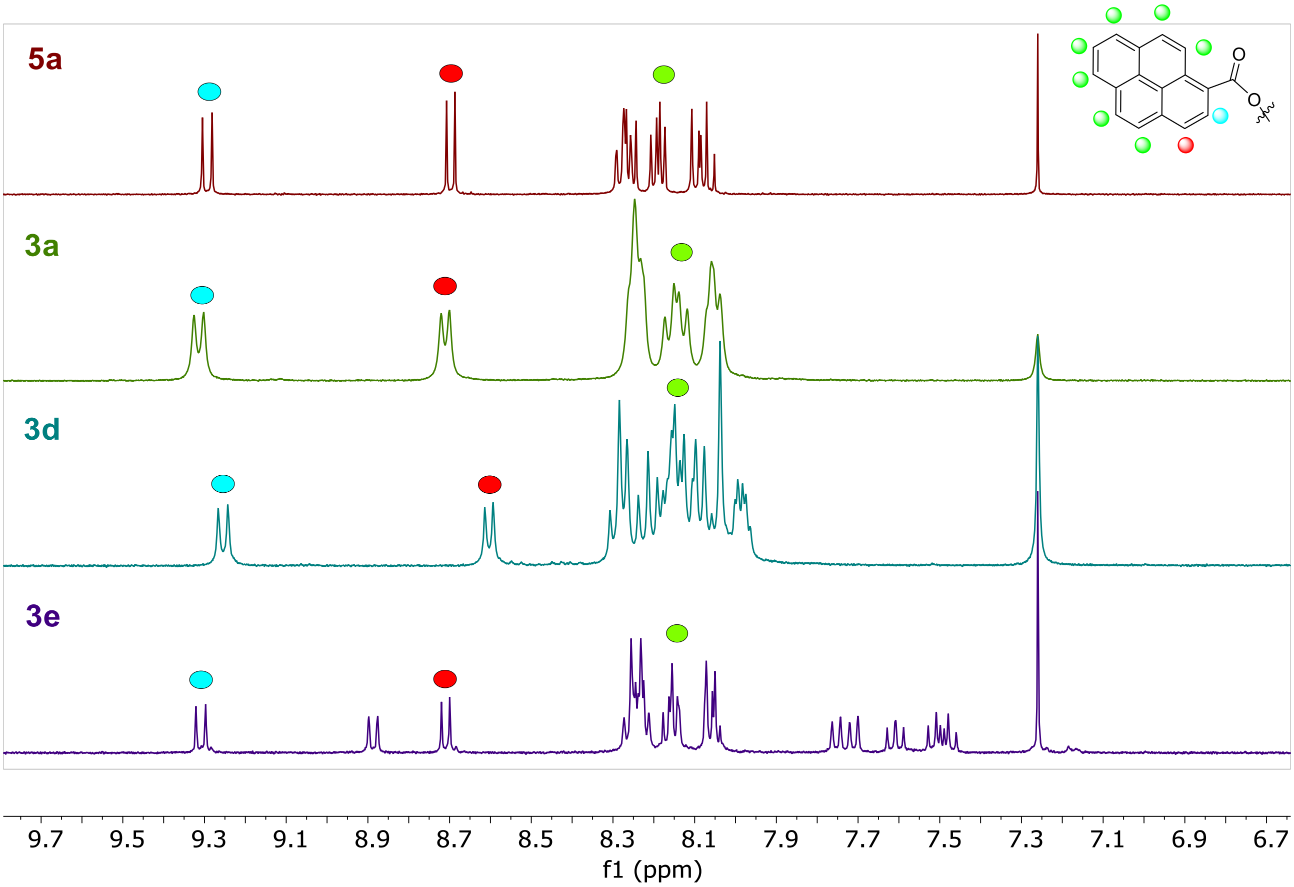

Figure S34. Expansion of the NMR spectra (401 MHz, Chloroform-*d*) of compounds **5a**, **3a**, **3d**, **3e**, the dots represent the hydrogen of interest (see structure above).

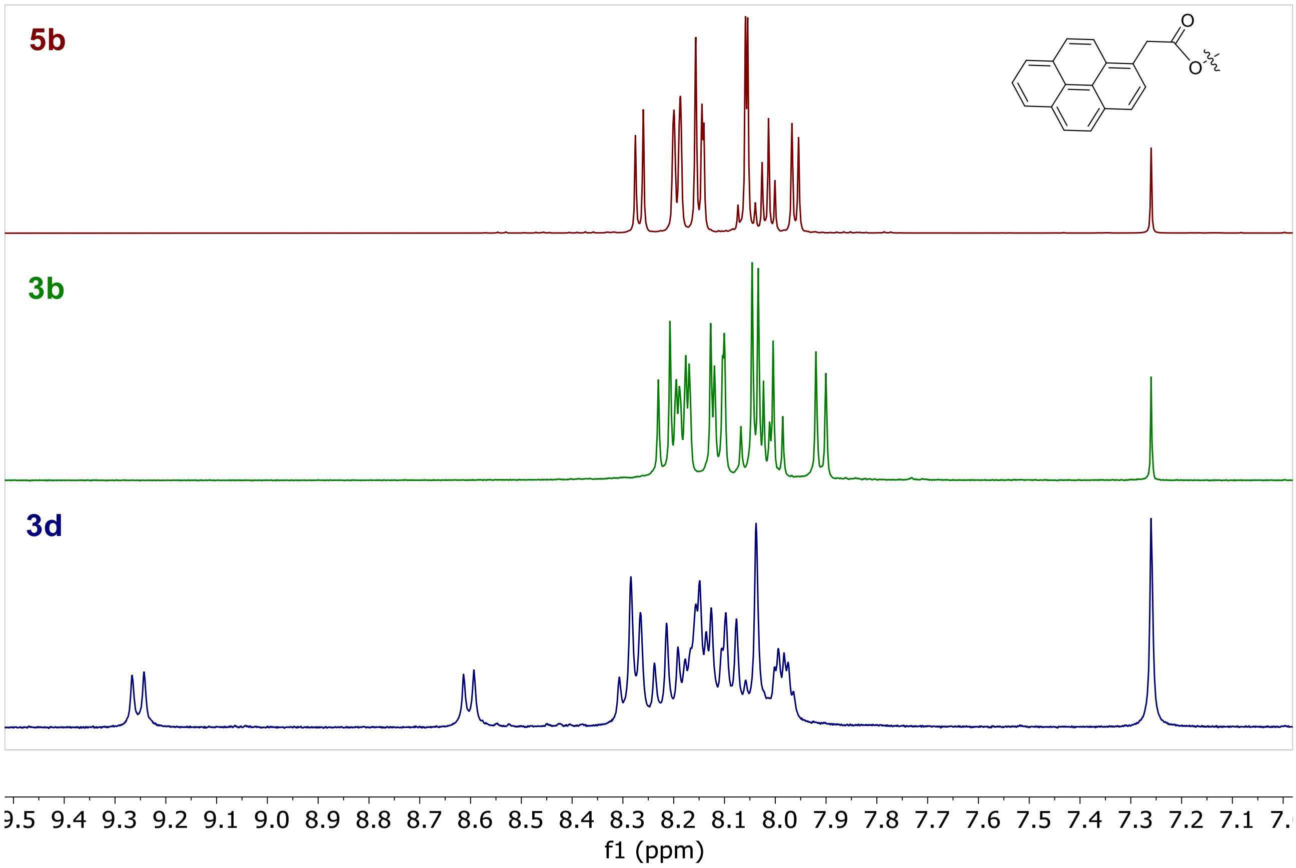

Figure S35. Expansion of the NMR spectra (401 MHz, Chloroform-*d*) of compounds **5b**, **3b**, **3d**.

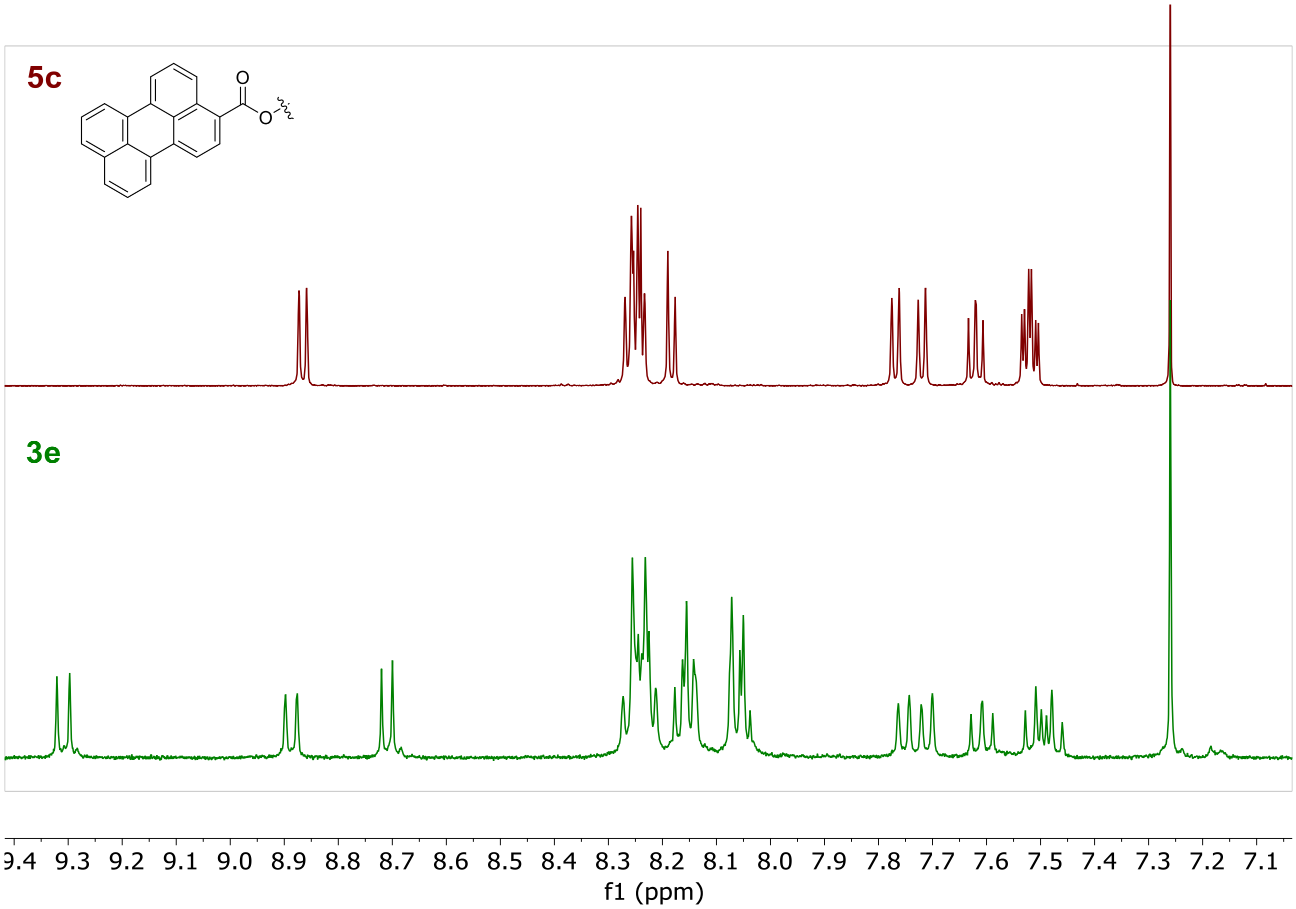

Figure S36. Expansion of the NMR (401 MHz, Chloroform-*d*) spectra of compounds **5c**, **3e.**

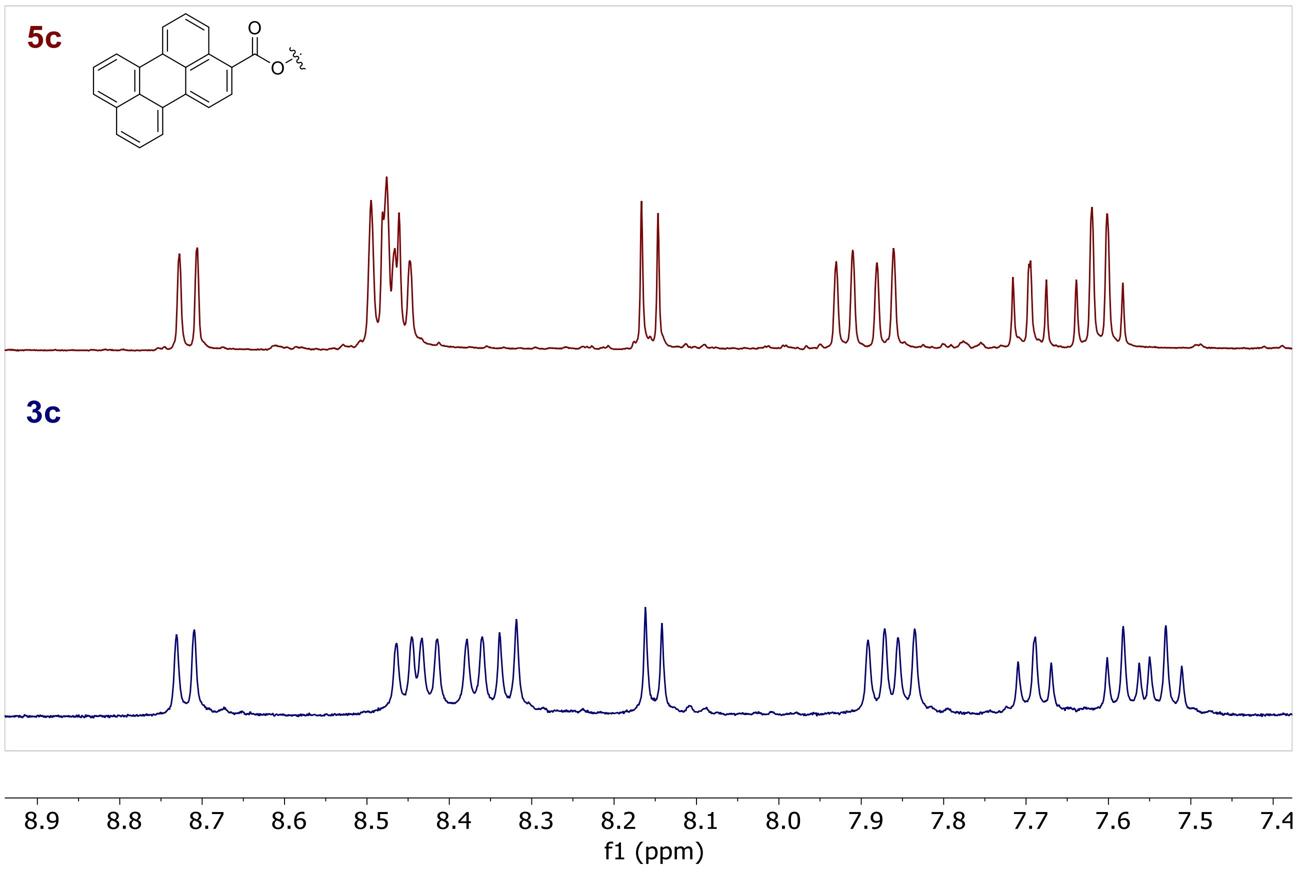

Figure S37. Expansion of the NMR (401 MHz, DMSO-*d*$_6$.) spectra of compounds **5c**, **3c**.

5 Computational details

5.1 Compound 3a

Conformational searches and preliminary DFT calculations were run with Spartan'18 (Wavefunction, Inc., Irvine CA, 2018), with standard parameters and convergence criteria. The molecular structure of compound **3a** was constructed in Spartan'18 GUI and relaxed with Merck Molecular force field (MMFF). A conformational search was run using the Monte Carlo algorithm, and MMFF, by varying the rotatable bonds (C3–O, C6–O, (O=)C–pyrenyl) and the puckering of the bicyclic system. All structures thus obtained were screened by single-point calculations at B3LYP-D3/6-31G(d) level, keeping all conformers with relative energies within 10 kcal/mol, which were then optimized at the same level of theory, in vacuo. This procedure resulted in 6 different conformers with relative energies within 10 kcal/mol.

Final DFT geometry optmizations were run with Gaussian 16[4] using standard grids and convergence criteria. The set of conformers obtained as described above was optimized with two functionals (B3LYP and BH&HLYP),[5,6] either without or with a dispersion correction (B3LYP-D3, BH&HLYP-D2);[7,8] in all casis, def2-SVP basis set[9,10] was employed, in vacuo. The results of ground-state (GS) geometry optmizations are summarized in **Table S1**. The structures calculated at B3LYP/def2-SVP and B3LYP-D3/def2-SVP level are shown in **Figures S38** and **S39**, respectively. As expected, dispersion corrections favor conformers first with face-to-face π-π stacked pyrene rings, and then with face-to-edge π-H stacked rings, over non-stacked conformers. The opposite occurs without dispersion correction. It is noteworthy that, starting from the same set of preoptimized structures, the four combinations converge toward very different sets of optimized structures.

Table S1. Low-energy conformers calculated for **3a** using four different functionals and def2-SVP basis set, in vacuo.

**Conf.**	**B3LYP**		**B3LYP-D3**		**BH&HLYP**		**BH&HLYP-D2**	
	Energy[a]	Structure type[b]	Energy[a]	Structure type[b]	Energy[a]	Structure type[b]	Energy[a]	Structure type[b]
GS#1	+2.62	LS	+0.36 [c]	S	+2.28	LS	+0.65	S
GS#2	+3.43	S	0	S	+2.53	S	0	S
GS#3	0	N	+0.36 [c]	S	+0.02	N	+9.75	H
GS#4	+0.57	H	+4.45	S	+0.32	H	+7.12	S
GS#5	+0.17	N	+10.63	N	0	N	+12.01	N
GS#6	+1.35	H	+5.72	S	+0.90	H	+0.65	S

[a] Relative internal energy in kcal/mol from the lowest energy minimum optimized at the same level of calculation

[b] Legend: S, parallel π-π stacked; LS, loosely (non-parallel) π-π stacked; H, π-H T-stacked; N, non-stacked

[c] Duplicate structure

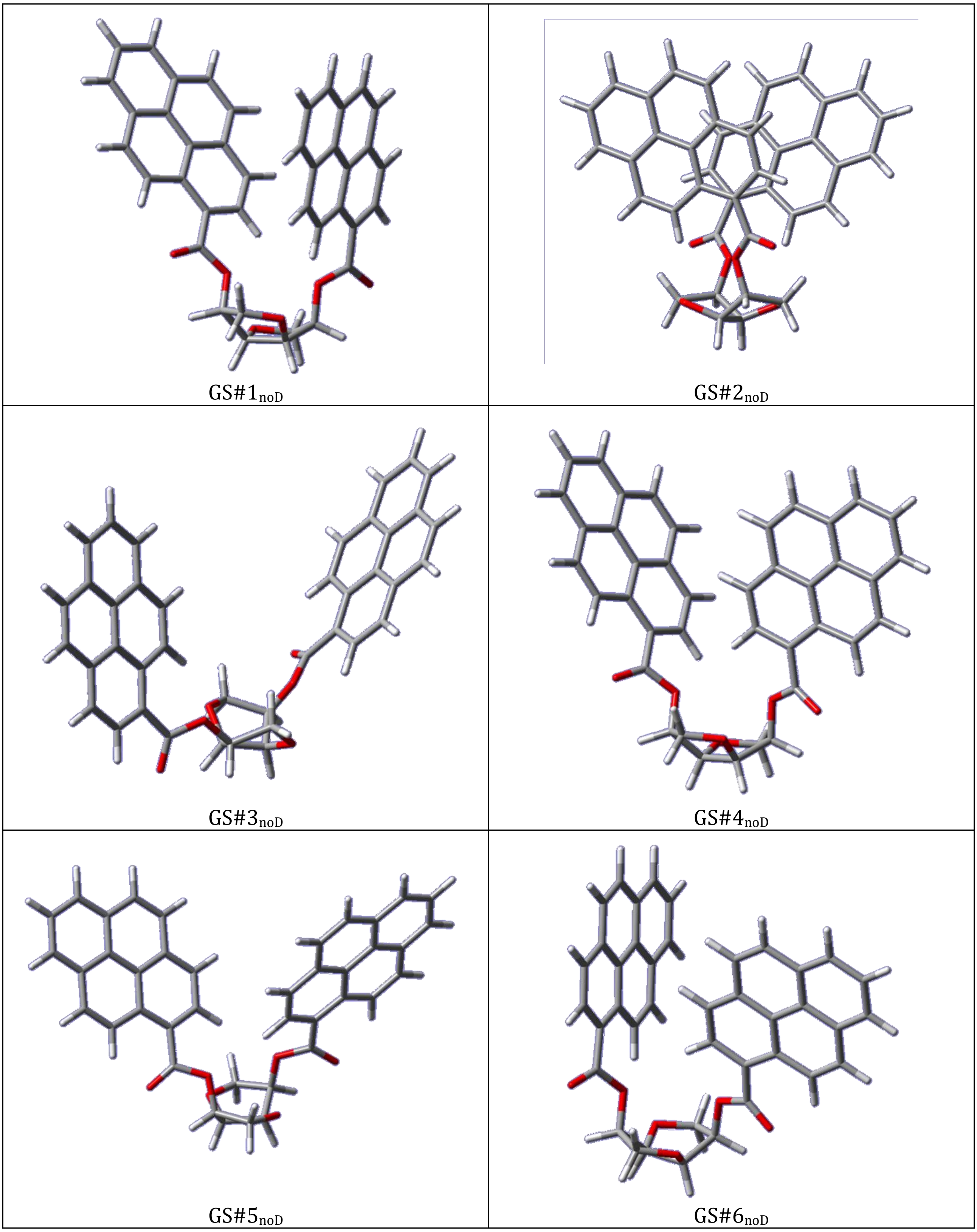

Figure S38. Low-energy conformers calculated for **3a** at B3LYP/def2-SVP level, in vacuo; energies reported in **Table S1.** The subscript "noD" indicates no dispersion used in the geometry optimizations.

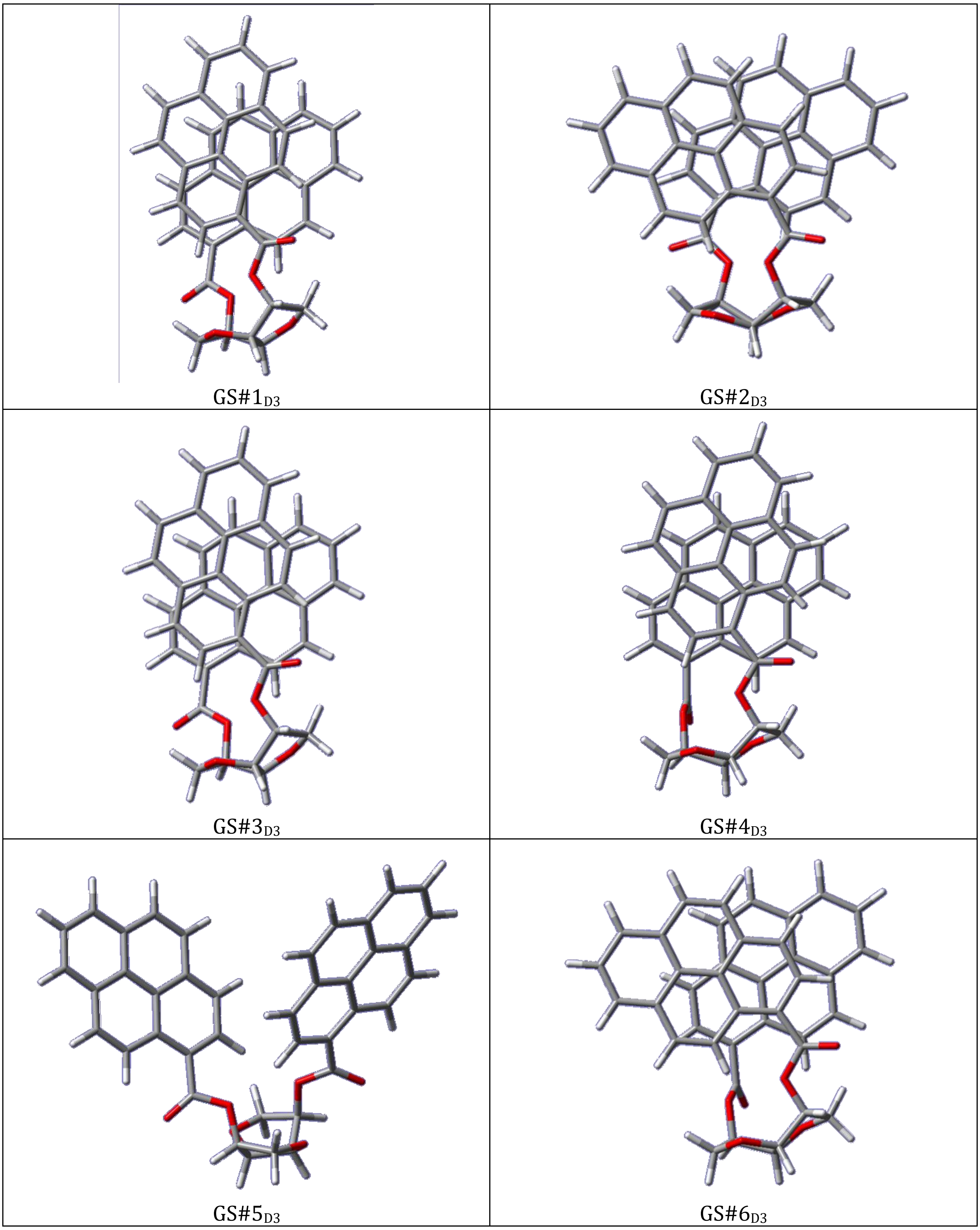

Figure S39. Low-energy conformers calculated for **3a** at B3LYP-D3/def2-SVP level, in vacuo; energies reported in **Table S1**. The subscript "D3" indicates D3 dispersion used in the geometry optimizations.

TD-DFT ECD calculations were run with Gaussian 16,[4] with default grids and convergence criteria. The lowest-energy geometries optmized at B3LYP-D3/def2-SVP level (GS#1_{D3} and GS#2_{D3}) and at B3LYP/def2-SVP level (GS#3_{noD}-GS#6_{noD}) were used as input structures. ECD calculations were run using B3LYP, BH&HLYP and CAM-B3LYP functionals, def2-SVP basis set, in vacuo, including 36 roots. In the long-wavelength region, the three functionals behaved similarly, apart from a wavelength shift (BH&HLYP and CAM-B3LYP-calculated transitions were blue-shifted by ≈40-50 nm with respect to B3LYP). Weighted-average ECD spectra calculated with the six combinations are shown in Figure **S40**. Regardless of the type of conformer structure (as classified in **Table S1**), all structures consistently led to moderate or strong exciton couplets in the long–wavelength region, which in some cases cancel each other in the average spectra. These latter are anyway very far from the experimental ECD spectrum of **3a** (**Figure 3E** in the main text). Since most of the low-energy conformers display interacting pyrene rings (either by π-π or π-H stacking), which is at odds with NMR data, we believe that the failure of ECD calculations may be attributed to the fact that our conformational ensemble is biased toward stacked geometries, while the real conformational situation features a much more flexible arrangement between the two chomophores. A second possible reason for the failure is the well-known difficulty of DFT in predicting the correct energy ordering of pyrene transitions,[11] although the correct order should be recovered by using CAM-B3LYP.[12]

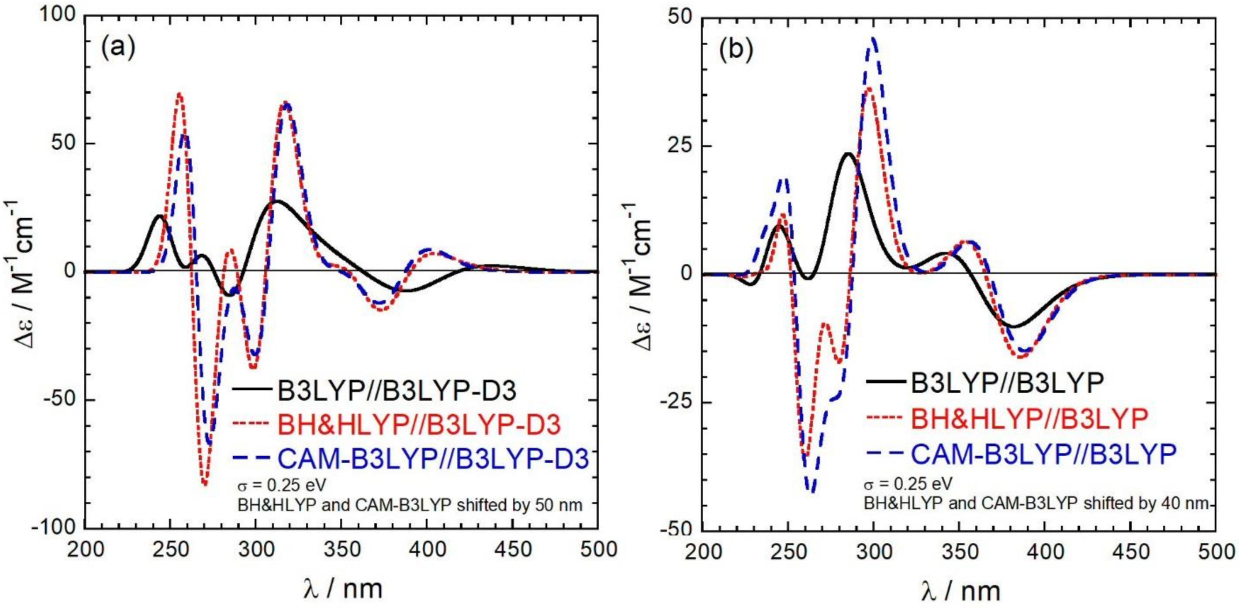

Figure S40. ECD calculated at B3LYP/def2-SVP, BH&HLYP/def2-SVP and CAM-B3LYP/def2-SVP level as weighted averages on low-energy conformers optimized at (a) D3-B3LYP/def2-SVP and (b) B3LYP/def2-SVP level, in vacuo. Spectra plotted with SpecDis v 1.51[13] from dipole-length rotational strengths, plotting parameters shown in the inset.

Excited-state geometry optmizations, also leading to emission and CPL spectra, were run with ORCA 5.0,[14–16] with default options and grids, and tight SCF convergence criteria. The low-energy geometries optmized at B3LYP-D3/def2-SVP level (GS#1_{D3}, GS#2_{D3} GS#4_{D3}-GS#6_{D3}) were used as starting structures. The first excited state was optimized with B3LYP, B3LYP-D3, BH&HLYP, and BH&HLYP-D2 functionals, using in all cases the def2-SVP basis set (with the resolution of identity approximation for Coulomb integrals and COSX numerical integration for HF exchange),[17] in vacuo, and including 8 roots. The results are summarized in **Table S2**, and selected excited structures are shown in **Figure S41**. Apart from B3LYP, excited-state calculations with all other functionals consistently converged toward a small set of excimer-like structures with short ring-to-ring distances (< 3.5 Å). **Table S3** reports calculated emission data for the lowest-energy excited-state structures ES#1 and ES#2.

Table S2. Low-energy conformers calculated for the first excited state of **3a** using four different functionals and def2-SVP basis set, in vacuo. The starting geometries were the corresponding GS#nD_3 structures shown in **Table S1** and **Figure S39**, optimized at B3LYP-D3/def2-SVP level.

Conf.	B3LYP		B3LYP-D3		BH&HLYP		BH&HLYP-D2	
	Energy[a]	Structure type[b]	Energy[a]	Structure type[b]	Energy[a]	Structure type[b]	Energy[a]	Structure type[b]
ES#1	0 [c]	H	0 [d]	SE	0 [e]	SE	0 [f]	SE
ES#2	+6.21	SD	+3.13	SD	+6.16	SD	+5.34	SD
ES#4	0 [c]	H	0 [d]	SE	0 [e]	SE	0 [f]	SE
ES#5	0 [c]	H	+10.35	H	+8.70	NS	+14.37	H
ES#6	+1.83	H	0 [d]	SE	+9.21	H	+10.21	S

[a] Relative internal energy in kcal/mol from the lowest energy minimum at the same level

[b] Legend: SE, parallel π-π stacked, excimeric (ring-to-ring distance < 3.5 Å); SD, π-π stacked displaced; H, π-H T-stacked; N, non-stacked

[c,d,e,f] Duplicate structure

Table S3. Summary of results from excited-state calculations on **3a** with various functionals and def2-SVP basis set, in vacuo. Relative energies reported in **Table S2**, and optmized structures reported in **Figure S41**.

Conf.	B3LYP			B3LYP-D3			BH&HLYP			BH&HLYP-D2		
	λ_{em}[a]	Stokes shift[b]	g_{lum} 10^{-2}	λ_{em}[a]	Stokes shift[b]	g_{lum} 10^{-2}	λ_{em}[a]	Stokes shift[b]	g_{lum} 10^{-2}	λ_{em}[a]	Stokes shift[b]	g_{lum} 10^{-2}
ES#1	573	146	−0.09	603	176	+12.5	452	112	+8.0	665	352	+15.3
ES#2	522	98	−0.22	542	118	−0.14	388	50	+0.7	554	216	−1.1

[a] In nm

[b] In nm, referred to the excitation wavelength calculated at the same level on the corresponding ground-state structure

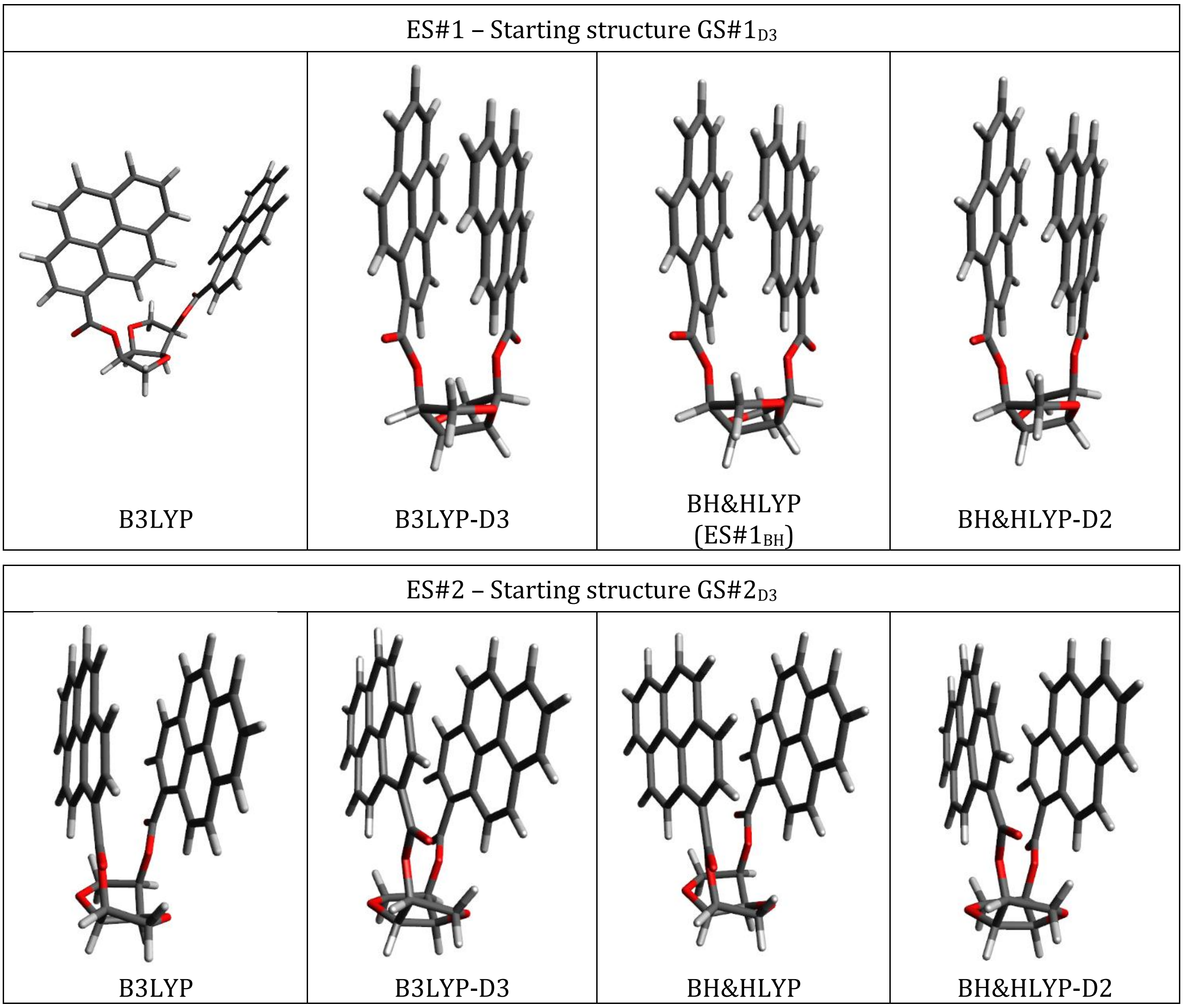

Figure S41. First excited-state geometries for **3a** with various functionals and def2-SVP basis set, in vacuo, starting from two different ground-state geometries ($GS\#1_{D3}$ and $GS\#2_{D3}$) optimized at B3LYP-D3/def2-SVP level; emission data reported in **Table S2**.

From the above calculations, structure ES#1_{BH} optimized at BH&HLYP/def2-SVP level in vacuo looked the most promising. Not only ES#1 was much more stable than ES#2 at all calculation levels, but the results obtained at BH&HLYP/def2-SVP level were also the closest ones to the experimental data for **3a** (Stokes shift and measured g_{lum}); further analysis was therefore run on this structure. First, the excited-state geometry was re-optimized at BH&HLYP/def2-SVP level using CPCM solvent model for dichloromethane.[18] Then, this latter structure (shown in the main text, **Figure 5**) was used for single point calculations, leading to emission and CPL data, with various functionals (B3LYP, BH&HLYP, CAM-B3LYP)[19] and basis sets (def2-SVP and def2-TZVP),[9,10] including CPCM solvent model for dichloromethane (**Table S4**). The inclusion of a conituum solvent model has a modest impact on emission wavelengths but a moderate impact on calculated g_{lum} values. BH&HLYP and CAM-B3LYP functionals performed similarly. The impact of the basis set was also limited, in terms of calculated Stokes shift and g_{lum}. Overall, the agreement with experimental Stokes shift (116 nm) and g_{lum} (+9.6· 10^{-3}) is rather good with both BH&HLYP and CAM-B3LYP functionals, using either of the two basis sets, and including the CPCM solvent model. **Figure S42** shows the frontier molecular orbitals calculated for the lowest-energy excited-state structure. They are clearly associated with an excimer state resulting from the combination of ^1L_b states of the two pyrene rings.

Table S4. Summary of results from excited-state calculations on **3a** with various functionals and basis sets, with CPCM model for dichloromethane, using as input structure the excited-state geometry optimized at BH&HLYP/def2-SVP level with CPCM model for dichloromethane (see **Figure 5**, main text).

**Functional**	**Basis set**	**Environment**	λ_{em}[a]	**Stokes shift**[b]	g_{lum} 10^{-2}
BH&HLYP	Def2-SVP	In vacuo[c]	451.5	111.6	+8.0
BH&HLYP	Def2-SVP	CPCM	452.3	107.0	+3.7
BH&HLYP	Def2-TZVP	CPCM	460.8	109.4	+3.9
B3LYP	Def2-SVP	CPCM	541.0	113.2	+5.1
CAM-B3LYP	Def2-SVP	CPCM	449.2	104.0	+3.6
CAM-B3LYP	Def2-TZVP	CPCM	464.7	112.7	+4.1

[a] In nm

[b] In nm, referred to the excitation wavelength calculated at the same level on the ground-state structure optimized at B3LYP-D3/def2-SVP level with CPCM for dichloromethane

[c] Data from **Table S2**

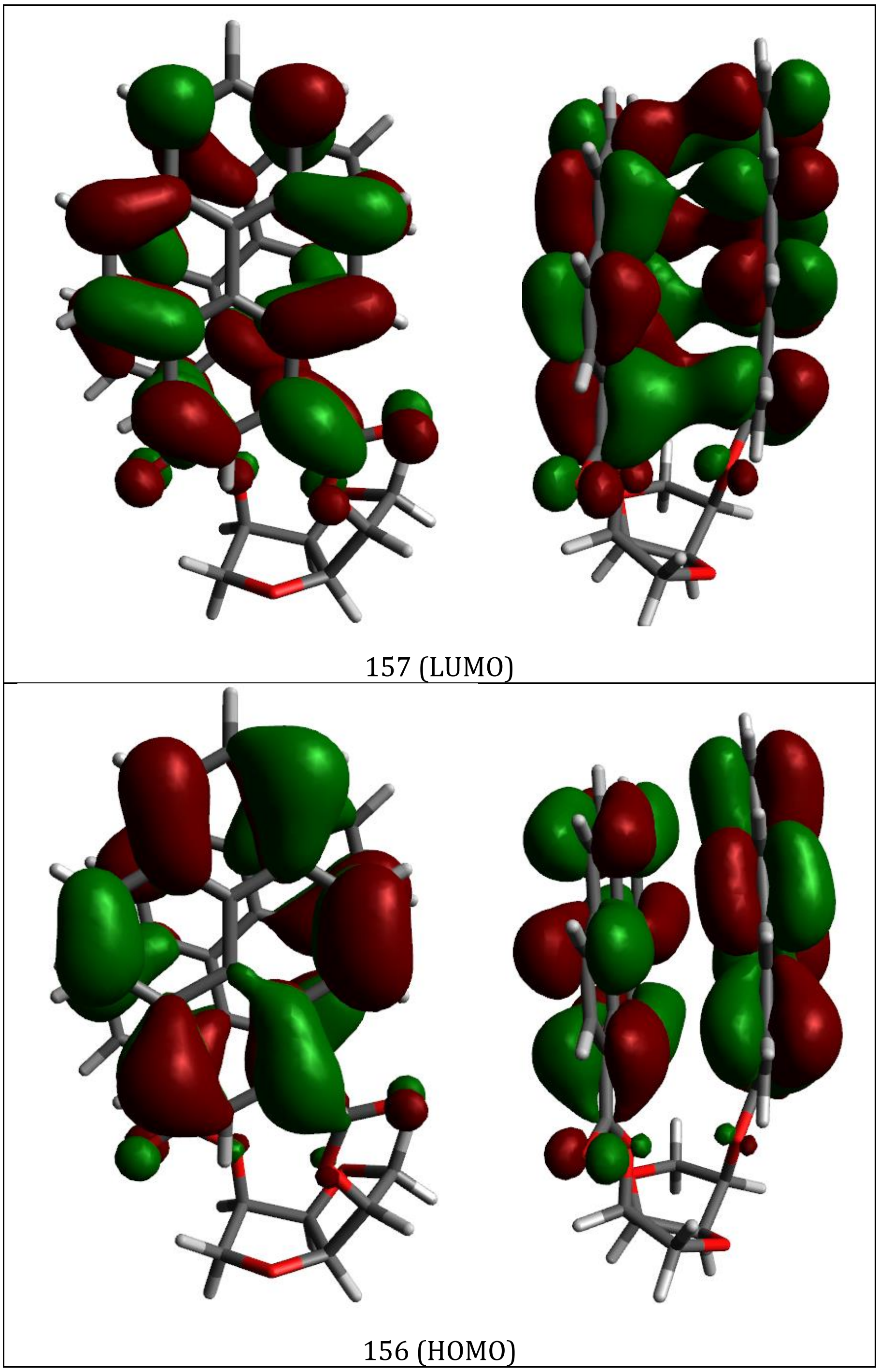

Figure S42. Frontier MOs calculated with def2-SVP basis set for the 1st excited-state structure of **3a**, optimized at BH&HLYP/def2-SVP level with CPCM model for dichloromethane.
Each MO is seen from two different perspectives. Isovalue 0.02.

5.2 Compound 3d

Conformational searches and preliminary DFT calculations were run with Spartan'18 (Wavefunction, Inc., Irvine CA, 2018), with standard parameters and convergence criteria. The molecular structure of compound **3d** was constructed in Spartan'18 GUI and relaxed with Merck Molecular force field (MMFF). A conformational search was run using the Monte Carlo algorithm, and MMFF, by varying the rotatable bonds (C3–O, C6–O, (O=)C–pyrenyl, (O=)C–CH_2, CH_2–pyrenyl,) and the puckering of the bicyclic system. Because of the very large number of MMFF conformers (>300), only those with pyrene-to-pyrene short contacts (< 5Å between the ring centers) were retained. Such structures were screened by single-point calculations at B3LYP-D3/6-31G(d) level, keeping all conformers with relative energies within 10 kcal/mol, which were then optimized at the same level of theory, in vacuo. This procedure resulted in 16 different conformers with relative energies within 10 kcal/mol, which were considered for further calculations.

Final DFT geometry optmizations were run with Gaussian 16[4] using standard grids and convergence criteria. The set of conformers obtained as described above was optimized with B3LYP-D3 functional[5,7] and def2-SVP basis set,[9,10] in vacuo. The results of ground-state (GS) geometry optmizations are summarized in **Table S5** and **Figures S43**.

Table S5. Low-energy conformers calculated for **3d** at B3LYP-D3/ def2-SVP level, in vacuo.

Conf.	Energy[a]	Structure type[b]
GS#01	0 [c]	S
GS#02	+0.23 [d]	S
GS#03	+2.49	LS
GS#04	+1.77	S
GS#05	0 [c]	S
GS#06	+1.30	S
GS#07	+2.13	S
GS#08	+2.97	S
GS#09	+6.30	H
GS#10	+6.02	N
GS#11	+3.09	S
GS#12	+3.22	S
GS#13	+5.81	LS
GS#14	+4.53	N
GS#15	+5.01	S
GS#16	+4.29	S

[a] Relative internal energy in kcal/mol from the lowest energy minimum optimized at the same level of calculation

[b] Legend: S, parallel π-π stacked; LS, loosely (non-parallel) π-π stacked; H, π-H T-stacked; N, non-stacked

[c,d] Duplicate structure

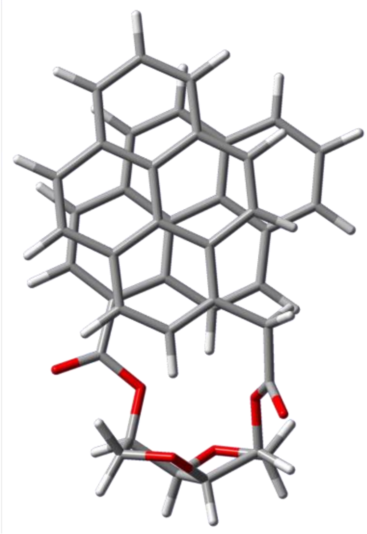<br>GS#01	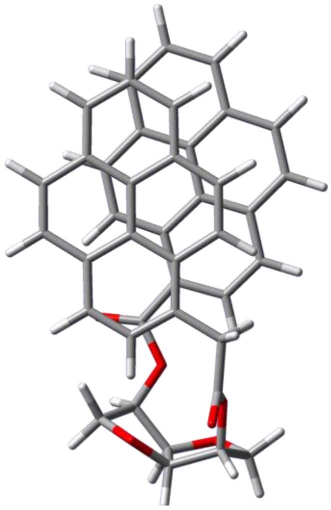<br>GS#02
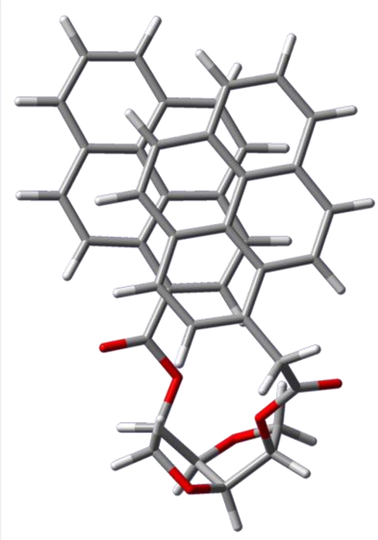<br>GS#03	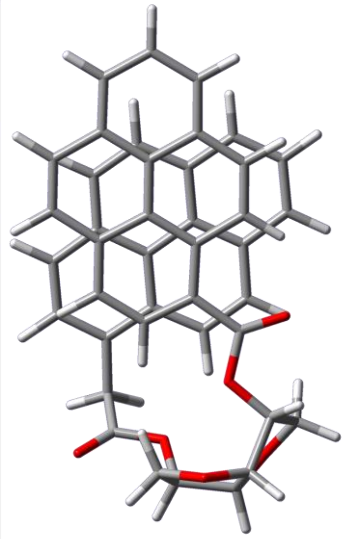<br>GS#04
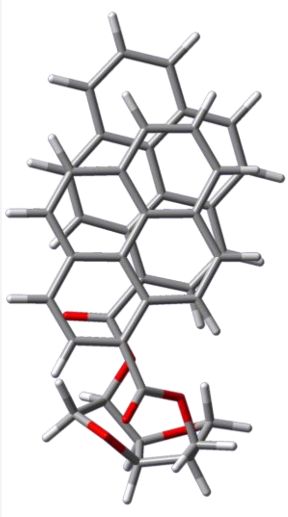<br>GS#06	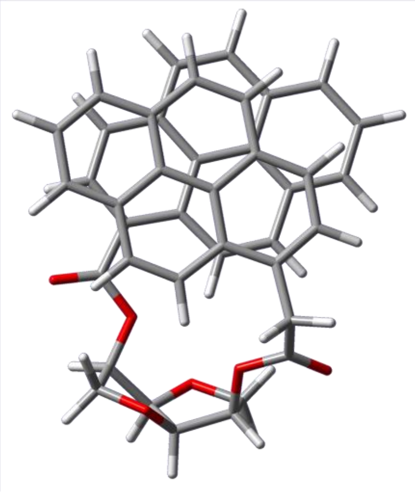<br>GS#07

Figure S43. Lowest-energy conformers calculated for **3d** at B3LYP-D3/def2-SVP level, in vacuo; energies reported in **Table S5**.

Excited-state geometry optmizations, also leading to emission and CPL spectra, were run with ORCA 5.0,[14–16] with default options and grids, and tight convergence criteria for SCF. The low-energy geometries optmized at B3LYP-D3/def2-SVP level (**Table S5**) were used as starting structures. The first excited state was optimized with B3LYP-D3 and BH&HLYP functionals and the def2-SVP basis set (with the resolution of identity approximation for Coulomb integrals and COSX numerical integration for HF exchange),[17] in vacuo, and including 8 roots. The results are summarized in **Table S6** and selected excited structures are shown in **Figure S44.**

The situation for compound **3d** is much more hetereogenoeus than for **3a**; in particular, the two functionals led to different sets of low-energy excited state structures, although they both identify ES#14 as the lowest-energy structure, displaying non-stacked pyrene rings. Molecular orbital (MO) analysis (not shown) assigns the 1st excited state of ES#14 as a charge-transfer state among the two aromatic rings, rather than an excimer. Thus, the present computational procedure does not seem adequate to capture the excimer structure of the more flexible compound **3d**.

Table S6. Low-energy conformers calculated for the first excited state of **3d** using two different functionals and def2-SVP basis set, in vacuo. The starting geometries were the corresponding GS#n structures shown in **Table S5** and **Figure S44**, optimized at B3LYP-D3/def2-SVP level.

**Conf.**	**B3LYP-D3**		**BH&HLYP**	
	Energy[a]	Structure type[b]	Energy[a]	Structure type[b]
ES#01	+9.88	SE	+1.01	SE
ES#02	+6.54	SE	+0.23	SD
ES#03	+5.01	SD	+2.17	SD
ES#04	+4.36	SD	+1.53	SE
ES#06	+6.20	SE	+3.27	SE
ES#07	+11.42	SE	+5.59	SE
ES#08	+9.66	SE	+3.63	SD
ES#09	+6.79	N	+3.68	N
ES#10	+6.90	N	+6.69	H
ES#11	+10.59	SE	+6.08	S
ES#12	+10.53	SE	+6.14	S
ES#13	+12.10	SD	+7.24	H
ES#14	0	N	0	N
ES#15	+10.56	SE	+3.76	N
ES#16	+9.60	S	+3.74	S

[a] Relative internal energy in kcal/mol from the lowest energy minimum at the same level

[b] Legend: S parallel π-π stacked; SE, parallel π-π stacked, excimeric (ring-to-ring distance < 3.5 Å); SD, π-π stacked displaced; H, π-H T-stacked; N, non-stacked

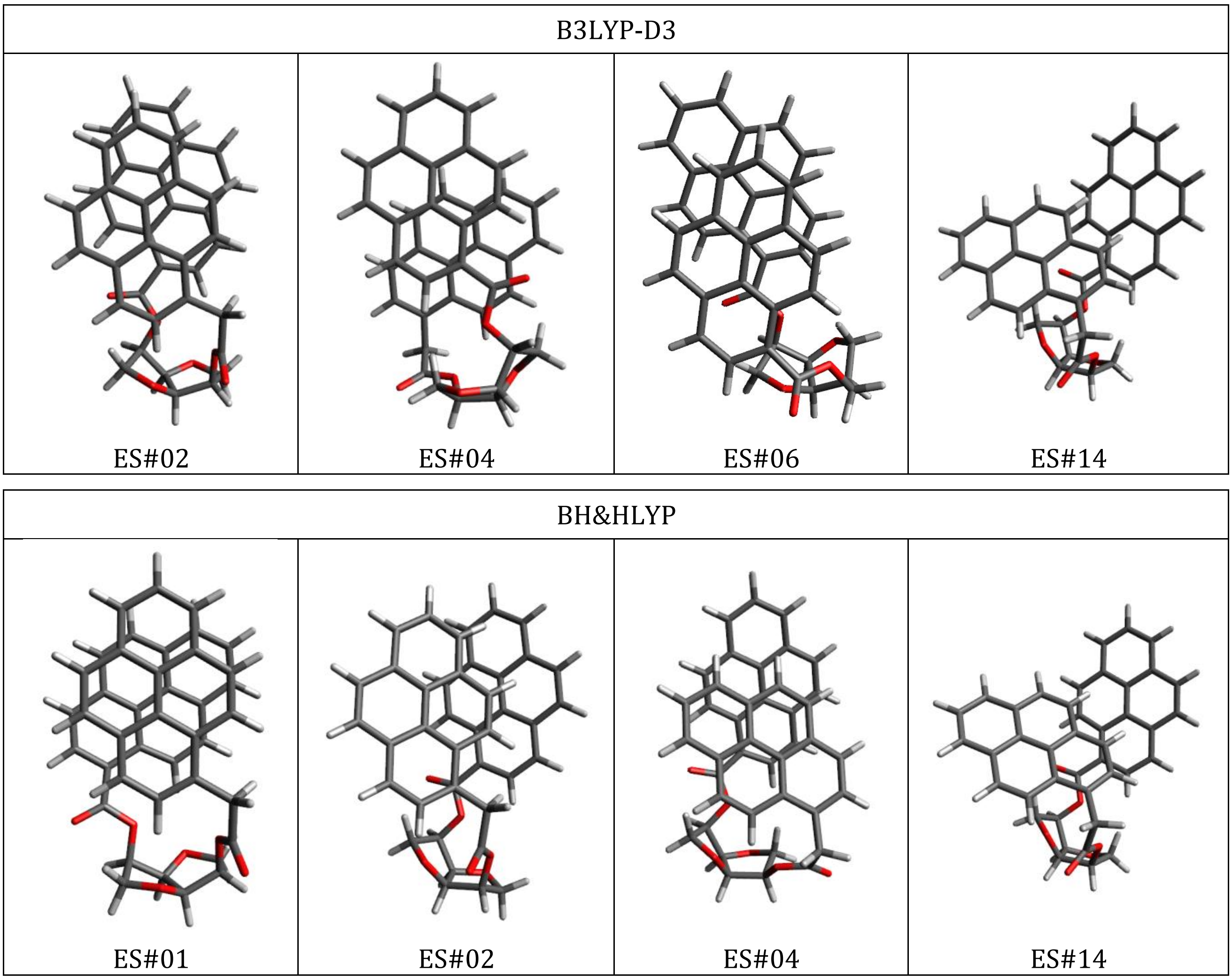

Figure S44. Selected first excited-state geometries for **3d** optimized with two functionals and def2-SVP basis set, in vacuo, starting from ground-state geometries optimized at B3LYP-D3/def2-SVP level. Energies reported in **Table S6**.

5.3 Chromophoric models

The model chromophores methyl pyrene-1-carboxylate (pyr-COOMe) and methyl 2-(pyren-1-yl)acetate (pyr-CH_2COOMe) were constructed in Spartan'18 GUI and relaxed with Merck Molecular force field (MMFF). A conformational search was run using the Monte Carlo algorithm, and MMFF. All structures thus obtained were optimized at B3LYP-D3/6-31G(d) level. This procedure resulted in 2 different conformers for each compound with relative energies within 10 kcal/mol. Only the lowest-energy structure was considered for TD-DFT calculations.

TD-DFT calculations were run with Gaussian 16,[4] with default grids and convergence criteria, using CAM-B3LYP and BH&HLYP functionals, def2-SVP basis set, and including including CPCM solvent model for dichloromethane. **Table S7** and **Figures S45-46** report the calculated parameters and relevant molecular orbitals (MO) for ^1L_b transition. It can be seen that the substitution with COOMe leads to a small red-shift and a more than tenfold increase of the intensity (oscillator strength) for the ^1L_b transition. For pyr-COOMe, the frontier π-type orbitals display sizable delocalization on the carboxylate group.

Table S7. Summary of results from excited-state calculations on pyr-COOMe and pyr-CH_2COOMe with various functionals and def2-SVP basis set, with CPCM for acetonitrile.

Compound	CAM-B3LYP			BH&HLYP		
	λ_{max}[a]	f[b]	CI	λ_{max}[a]	f[b]	CI
Pyr-COOMe	325	0.038	67→69 (0.54)<br>68→70 (0.42)	318	0.0273	67→69 (0.55)<br>68→70 (0.42)
Pyr-CH_2COOMe	321	0.0020	71→73 (0.45)<br>72→74 (0.53)	314	0.0026	71→73 (0.44)<br>72→74 (0.54)

[a] In nm

[b] Oscillator strength (au)

[c] Major contributions from single-electron excitations: occupied MO → virtual MO (coefficient). MOs are shown in **Figures S45-S46**.

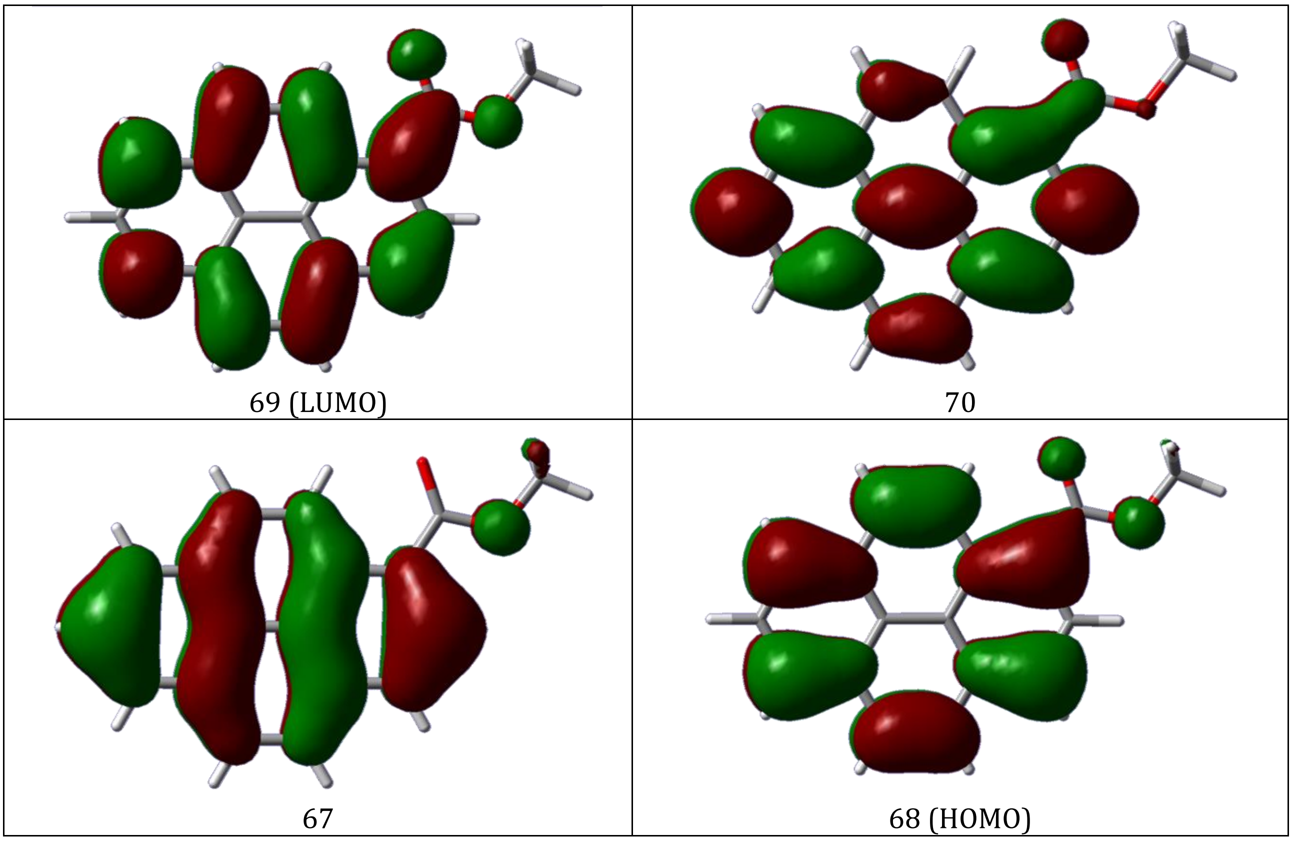

Figure S45. Frontier MOs calculated for pyr-COOMe with def2-SVP basis set (lowest-energy B3LYP-D3/6-31G(d) geometry). Isovalue 0.02.

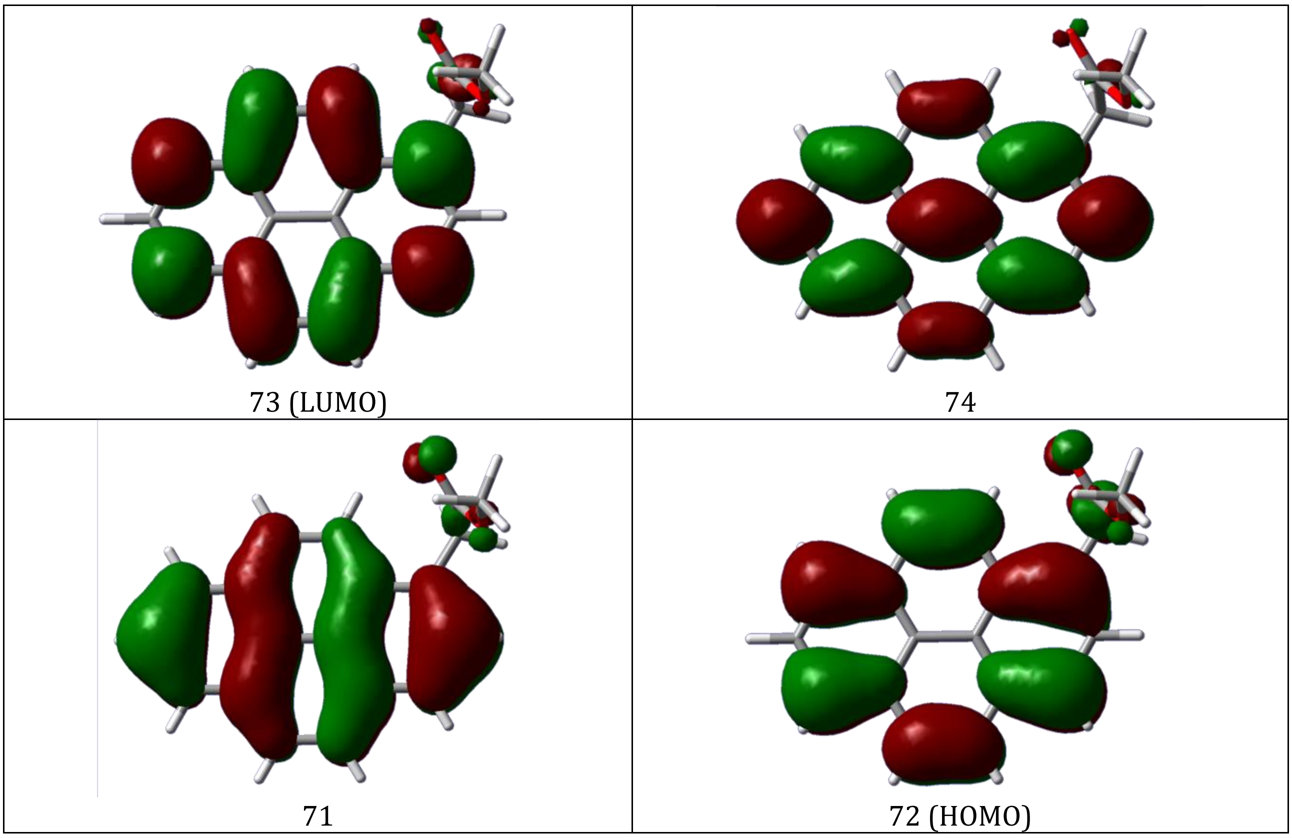

Figure S46. Frontier MOs calculated for pyr-CH_2COOMe with def2-SVP basis set (lowest-energy B3LYP-D3/6-31G(d) geometry). Isovalue 0.02.

6 References

[1] F. Zinna, T. Bruhn, C. A. Guido, J. Ahrens, M. Bröring, L. Di Bari, G. Pescitelli, *Chem. - A Eur. J.* **2016**, *22*, 16089–16098.

[2] A. A. Chistov, S. V. Kutyakov, A. V. Ustinov, I. O. Aparin, A. V. Glybin, I. V. Mikhura, V. A. Korshun, *Tetrahedron Lett.* **2016**, *57*, 1003–1005.

[3] I. V. Astakhova, V. A. Korshun, K. Jahn, J. Kjems, J. Wengel, *Bioconjug. Chem.* **2008**, *19*, 1995–2007.

[4] M. J. Frisch, G. W. Trucks, H. B. Schlegel, G. E. Scuseria, M. A. Robb, J. R. Cheeseman, G. Scalmani, V. Barone, G. A. Petersson, H. Nakatsuji, et al., **2016**.

[5] A. D. Becke, *J. Chem. Phys.* **1993**, *98*, 5648–5652.

[6] A. D. Becke, *J. Chem. Phys.* **1993**, *98*, 1372–1377.

[7] S. Grimme, *J. Comput. Chem.* **2006**, *27*, 1787–1799.

[8] S. Grimme, J. Antony, S. Ehrlich, H. Krieg, *J. Chem. Phys.* **2010**, *132*, 154104.

[9] F. Weigend, *Phys. Chem. Chem. Phys.* **2006**, *8*, 1057–1065.

[10] F. Weigend, R. Ahlrichs, *Phys. Chem. Chem. Phys.* **2005**, *7*, 3297–3305.

[11] M. Parac, S. Grimme, *Chem. Phys.* **2003**, *292*, 11-21.

[12] E. L. Graef, J. B. L. Martins, *J. Mol. Model.* **2019**, *25*, 183.

[13] T. Bruhn, A. Schaumlöffel, Y. Hemberger, G. Pescitelli, *SpecDis version 1.71*, Berlin, Germany, **2017**, http://specdis-software.jimdo.com.

[14] C. Benkhauser-Schunk, B. Wezisla, K. Urbahn, U. Kiehne, J. Daniels, G. Schnakenburg, F. Neese, A. Lützen, *Chempluschem* **2012**, *77*, 396–403.

[15] F. Neese, *WIREs Comput. Mol. Sci.* **2018**, *8*, e1327.

[16] F. Neese, F. Wennmohs, U. Becker, C. Riplinger, *J. Chem. Phys.* **2020**, *152*, 224108.

[17] F. Neese, F. Wennmohs, A. Hansen, U. Becker, *Chem. Phys.* **2009**, *356*, 98–109.

[18] M. Garcia-Ratés, F. Neese, *J. Comput. Chem.* **2019**, *40*, 1816–1828.

[19] T. Yanai, D. P. Tew, N. C. Handy, *Chem. Phys. Lett.* **2004**, *393*, 51–57.
